# Colorectal liver metastasis: molecular mechanism and interventional therapy

**DOI:** 10.1038/s41392-022-00922-2

**Published:** 2022-03-04

**Authors:** Hui Zhou, Zhongtao Liu, Yongxiang Wang, Xiaoyong Wen, Eric H. Amador, Liqin Yuan, Xin Ran, Li Xiong, Yuping Ran, Wei Chen, Yu Wen

**Affiliations:** 1 Department of General Surgery, Second Xiangya Hospital, Central South University, Changsha, 410011 Hunan Province China; 2 Department of Physics, The University of Texas, Arlington, TX 76019 USA; 3 Department of Dermatovenereology, West China Hospital, Sichuan University, Chengdu, Sichuan Province China; 4 Medical Technology Research Centre, Chelmsford Campus, Anglia Ruskin University, Chelmsford, CM1 1SQ UK

**Keywords:** Cancer, Metastasis

## Abstract

Colorectal cancer (CRC) is one of the most frequently occurring malignancy tumors with a high morbidity additionally, CRC patients may develop liver metastasis, which is the major cause of death. Despite significant advances in diagnostic and therapeutic techniques, the survival rate of colorectal liver metastasis (CRLM) patients remains very low. CRLM, as a complex cascade reaction process involving multiple factors and procedures, has complex and diverse molecular mechanisms. In this review, we summarize the mechanisms/pathophysiology, diagnosis, treatment of CRLM. We also focus on an overview of the recent advances in understanding the molecular basis of CRLM with a special emphasis on tumor microenvironment and promise of newer targeted therapies for CRLM, further improving the prognosis of CRLM patients.

## Introduction

Colorectal cancer (CRC) is one of the three most common cancers and the fourth most common cause of cancer deaths worldwide.^1^ In 2020, 1.9 million cases were newly diagnosed with CRC and 935,000 cases with CRC past away.^2^ The global incidence of CRC has been rising with annual increases of 3.2%, beginning with 783,000 cases in 1999 and increasing to 1.8 million in 2020^2–7^; this trend is likely to continue. Accordingly, the global CRC burden is increasing mainly owing to the growth of human development index—The incidence rates in developing countries is a quarter of that in developed countries.^2^

Metastasis of CRC remains a major problem after curative treatment and is the critical cause of CRC-related death.^8^ The liver is the most common organ of distant metastasis in CRC.^9^ Liver metastasis of CRC may be associated with the following factors: the portal vein system directly connecting the colorectal and liver, which is associated with abundant blood supply; location and histological type of primary tumor.^10–12^ Curative resection and chemotherapy are the standard methods treatment in patients with colorectal liver metastasis (CRLM),^13^ However, due to factors such as the location and size of the tumor, unresectable disease, presence of extrahepatic disease, or patients’ comorbidities, surgery is only applicable in 10–20% of cases, with a 5-year survival rate as low as 30%.^14,15^ Furthermore, those who are not eligible for surgery have an even worse prognosis. Although, significant progress in the development of new chemotherapeutic drugs has been made, CRLM patients that receive fluorouracil and platinum chemotherapy initially, will eventually develop chemotherapy resistance due to inherent or acquired resistance. For CRLM, it is necessary to find more effective targeted therapies. Understanding of the molecular mechanisms underlined in this process could accelerate the achievement of this goal.

A small subset of CRC cells acquires a capacity to evade from the primary CRC, in part by morphological changes such as epithelial-to-mesenchymal transition (EMT), migration through the extracellular matrix (ECM), and invasion into the neighboring tissues, intravasation, survival in the circulation, extravasation and finally colonization to distant liver forming more aggressive secondary CRLM.

## Epidemiology

Nearly 50% of patients with CRC will develop liver metastases during the disease.^16^ The incidence of stage IV left colon cancer liver metastases is high.^17,18^ However, once liver metastasis occurred in right colon cancer, the range of liver metastasis was wider than that in left colon cancer.^19^ The probability of liver metastasis in CRC is also associated with gender. Males are more likely to be a risk factor of CRC, with higher illness burden and earlier onset age^2,4,20^ and 25–50% of these patients develop liver metastasis^21,22^, of which ~30% are diagnosed with CRC at the same time. Notably, ethnicity was likely to be correlated with the incidence of development of CRLM. Research in the United States alone reported that non-Hispanic black patients have the highest incidence of liver metastasis, followed by American Indian/Alaska Native, non-Hispanic white, Hispanic and Asian/Pacific Islanders.^23^ That is, the variations in genes might contribute to the CRLM. Prior works have suggested that BRAF, KRAS, NRAS, PI3KCA, TP53, NRAS, CDK12, EBF1 might be risk genes of CRLM.^24–27^ Furthermore, the types of gene mutations were also likely to be correlated with the prognosis^27^—the highest cure rates appeared in patients with NOTCH1 and PIK3C2B mutations, and the lowest in those with SMAD3 mutations.

## Mechanisms/pathophysiology

**Fig. 1:**
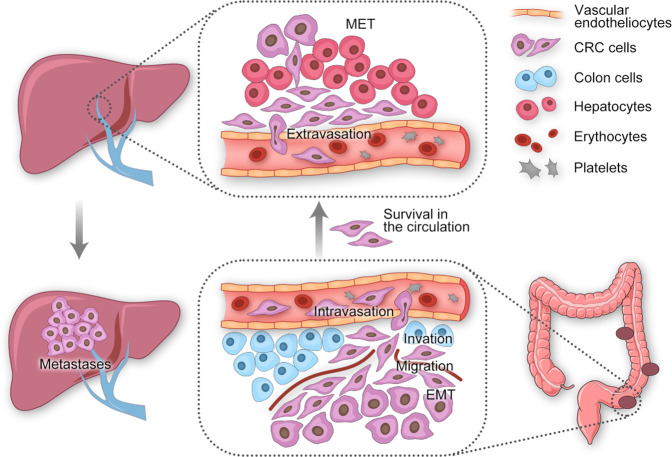
Schematic showing the liver metastasis cascade of CRC

In 1889, Paget proposed the concepts of “seed” (tumor cells) and “soil” (specific organs) for tumor metastasis.^28^ The liver metastatic cascade of CRC is a complex multi-factor and multi-step biological process (Fig. 1), wherein a small subset of CRC cells acquire a capacity to evade from the primary CRC, in part by morphological changes such as EMT, migration through the ECM, invasion into the neighboring tissues, intravasation, survival in the circulation, extravasation and finally, colonization to distant liver forming more aggressive secondary CRLM.^8,29^ On one hand, this process is related to genome abnormalities in the tumor cells themselves, including activation of proto-oncogenes and inactivation of tumor suppressor genes. The occurrence and development of CRLM is especially complex and involves many molecular mechanisms, including non-coding RNAs (ncRNAs), Notch pathway, TGFβ signaling, Tyrosine kinase c-MET signaling, phosphatase of regenerating liver (PRL3), tumor-associated calcium signal transducer 2(Trop-2), L1 cell adhesion molecule (L1CAM), S100 family proteins S100A4 and S100A8 and other pathways (Table 1). It is also closely related to the tumor microenvironment (TME), which mainly involves various immune cells (macrophages, T cells, B cell etc.), cytokines, chemokines and exsomes.^30–32^ The interaction of internal and external environment jointly initiates and drives the occurrence of CRLM.

**Table 1:** Chemokine ligand–receptor interactions between different cells further complicate the signal transduction in CRLM

Chemokine signaling	Ligand	Receptor	Function	Refs.
CCL9-CCL15	CRC	CD34(+) Gr-1(−) iMCs	Liver metastasis	^66^
CCL15-CCR1	CRC	CD11b(+), CD33(+),HLA-DR(−)MDSCs	Liver metastasis	^49,66^
CCL2-CCR2	CRC	CD11b(+)Gr-1(+) iMCs	Liver metastasis	^67,68^
CXCL1-CXCR2	CRC	CXCR2 + microvascular endothelial cells	Tumor angiogenesis	^69,70^
CXCL1-CXCR2	CRC	CXCR2 + macrophages	Liver metastasis	^71^
CXCL1-CXCR2	CRC	CXCR2 + neutrophil	Liver metastasis	^71^
CXCL1-CXCR2	TAMs	CXCR2 + MDSCs	Liver metastasis	^72^
CCL2-CCR2	CRC	TAMs	Liver metastasis	^37^
CXCL8-CXCR2	TAMs	CXCR2 + neutrophil	Liver metastasis	^75^

### Immune cells

Immune microenvironment plays a pivotal role in CRLM.^33^ The liver is an important immune organ of the human body. If its immune killing ability is weakened, it can promote the survival and growth of CRC metastases. Therefore, a suppressive immunologic microenvironment plays tumor-promoting role of CRLM, which is related to tumor-associated-macrophages (TAMs) and regulatory T cells (Treg). However, TAMs maintains the immunosuppressive environment by expressing checkpoint ligand programmed death ligand 1 (PDL1), PDL2 and other inhibitory receptors^34^, and activates Treg cells by secreting IL-10 and TGFβ.^35^ TAMs also release a plethora of ECM remodeling factors (plasminogen activation system, matrix metalloproteinases (MMPs), and kallikrein-related peptidases) and a diverse array of proteolytic enzymes such as MMPs that degrades the ECM proteins. These factors, in turn, enhance migration of tumor cells.^36^ In addition, when targeting the CCL2/CCR2 chemokine axis, TAMs infiltration at the metastatic site is reduced and metastatic CRC (mCRC) is sensitized to tumor T cells.^37^

Meanwhile, Treg cells inhibit aberrant immune response against self-antigens and antitumor immune response.^38^ The ability to inhibit adaptive antitumor immune responses via Treg is associated with clinical outcomes of CRLM.^39^ The intratumoral Treg could inhibit MMPs expression and activity with IL-17 producing T cells involved which reduces the probability of postoperative metastasis of CRC.^40^ Inhibition of Treg activity may be a therapeutic approach to improve antitumor immunity in the future.^39^

The tumor-associated neutrophils (TANs) may also promote tumor growth and metastasis through a variety of mechanisms.^41^ Hyperlipidemia can promote neutrophil infiltration, thereby increasing the metastasis of CRC.^42^ In the early phase of CRC cell dissemination, neutrophils expressed CCR1 exclusively and MMP9 preferentially, which contribute to the early expansion of cancer.^43^ TANs produce large amounts of lysine oxidase-like 4 protein during CRLM resistance anti-angiogenic therapy.^44^ TANs could be recruited by loss of SMAD4 promoting chemokine CCL15 expression via the CCL15-CCR1 axis.^45^ In addition, neutrophil extracellular traps (NET) trigger the release of HMGB1 and promote the migration and invasion of cancer cells during stress response.^41^ NETs could also directly trap CRC cells in liver boosted tumorous proliferation and invasion capacity, which is because of the heightened expression of tumorous interleukin (IL)-8 which is triggered by NETs.^46^ Interestingly, overproduced IL-8 in turn activated neutrophils towards NETs formation, thus forming a positive loop enhanced CRC liver metastasis. Therefore, eliminating NET may reduce risks of tumor relapse after surgical stress. Recently, Xia et al.^47^ reported neutrophil infiltration and NETs formation were inhibited in tumor tissues with Adeno‐associated virus‐mediated DNase I liver gene transfer treatment. NET-associated carcinoembryonic Ag cell adhesion molecule 1 (CEACAM1) as an essential element for inducing CRC metastatic phenotype, which prompted CEACAM1 as a potential therapeutic target for the prevention of CRC metastasis.

Myeloid-derived suppressor cells (MDSC) are another key factor that regulates the immune response under many pathological conditions, and immune suppressive activity is an intrinsic feature of MDSC. Neutrophils or monocytes that has undergone maturation is not able to transform into potent immune suppressive cells in vitro when simply activating MDSC with hazard associated molecular patterns (DAMPs) and pathogen-associated molecular patterns or pro-inflammatory cytokines.^48^ CCR1(+) MDSC are recruited to the microenvironment of disseminated CRC cells by loss of SMAD4 promoting chemokine CCL15 expression via the CCL15-CCR1 axis, and produce metalloproteinases MMP9 and MMP2 to enhance metastatic colonization.^43,49^ S1PR1–activator of transcription 3 (STAT3) upregulation in CRC cells promotes IL-6, which could activate S1PR1–STAT3 in MDSC in the liver, leading to premetastatic niche formation before CRC cell arrival.^50^

There has been some progress regarding the immune status of CRLM and the effect of tumor immunological characteristics on clinical outcome in CRLM patients. The density of macrophages in CRC is also closely related to patient prognosis.^51^ The CRLM T cell number is an independent correlate of long-term survival following liver resection, in particular, CD8 + T‐cell infiltration in liver metastasis is associated with better prognosis.^52–54^ Moreover, high frequencies of NK and T cells in response to chemotherapy predict OS in CRLM patients.^55^

### Cytokines

Cytokines IL-6 active IL-6 receptor (IL-6R), which induce STAT3 to bind to MIR34A inhibit miR-34A expression, thereby promoting EMT-mediated invasion and metastasis of CRC.^56^ Growth differentiation factor 15 in inflammatory microenvironment induces CRC metastasis by regulating EMT genes by activating c-Fos, which may be a new direction for CRLM treatment in the future.^57^ Similarly, cytokines IL-33 modulates the TME to potently induce the occurrence of liver metastasis.^58^ In addition, the levels of arachidonic acid (AA) and eicosapentaenoic acid (EPA) promoted the development of an inflammatory microenvironment and AA/EPA ratio was elevated levels in patients with metastatic CRC.^59^

### Chemokines

Chemokines, as an important component of TME, play an important role in the entry of tumor cells into the TME.^60^ For instance, chemokines are critical for the tumor-stroma interaction, which promote tumor metastasis via chemokines signaling. Chemokines could also act as a bridge between the microenvironment outside the tumor and the tumor itself, and their cognate receptors are expressed by both tumor and stromal cells.^61^ In recent years, it has been reported that chemokine ligands and receptors (such as CXCL5, CCL3, CCL4, CXCL2, CXCL3, CXCL8, CCL3L3, CCL4L2, CCL18) have been significantly dysregulated, which implies a potential intercellular communication in the immune microenvironment of CRC. Chemokine ligand–receptor interactions between different cells further complicate the signal transduction in CRLM (Table 1).

As a member of the CXC chemokine family, CXCL5 is the ligand of CXCR2 and is not only derived from primary tumor cells but is also secreted by immune cells in the TME.^62^

The CXCL5/CXCR2 biological axis promoted tumor angiogenesis by activating the AKT/NF-κB/FOXD1/VEGF-A pathway.^63^ Moreover, Zhao et al.^64^ found the elevated expression of CXCL5 in CRC induced cell migration by the ERK/Elk-1/Snail signaling pathway and promoted cell invasion through the AKT/GSK3β/β-catenin/MMP7 signaling pathway. They also found that high expression of CXCL5 was a favorable factor to promote the metastasis of CRC cells to the liver in nude mice intrasplenic injection model.

The earlier study confirmed that CCR1+ immature myeloid cells (iMCs) could migrate toward CRC-derived CCL9 and accumulate at tumor invasion front in mice model.^65^ Later studies demonstrated CRC cells secrete CC-chemokine ligands CCL9 and CCL15, respectively, and recruit CD34(+) Gr-1(−) iMCs to the metastatic liver.^66^ Additionally, CRC-derived CCL2 and Myeloid-derived S100A8/A9 enhanced CD11b(+)Gr-1(+) iMCs recruitment and increased tumor burden via CCL2/CCR2.^67,68^ Also, CRC-derived CXCL1 increased migration of CXCR2+ microvascular endothelial cells in vitro and mediated tumor angiogenesis by binding with CXCR2 receptor on endothelial cells in xenograft CRC models.^69,70^ Moreover, CXCL1 is found to recruit CXCR2+ neutrophil and macrophages from the bone marrow to premetastatic liver in CRC nude mice.^71^ It was also reported that CCL15 secreted by CRC attracted CCR1(+) MDSCs to the premetastatic niche via the CCL15-CCR1 axis, creating conditions conducive to metastasis.^49^ TAMs also recruited CXCR2-positive MDSCs to promote liver metastases via producing CXCL1.^72^ High expression of CCL4 in CRC could induce the infiltration of TAMs and specifically a pro-tumor macrophage profile (CD163 + cells).^73^ CCL2/CCR2 chemokine axis could also facilitate TAMs accumulation in liver metastasis and maintain immunosuppressive TME. In addition, CXCL8 and its receptor (CXCL8-R) promote liver metastasis by promoting angiogenesis and inducing EMT in CRC cells.^74^ Elevated serum CXCL8 levels are associated with poor prognosis in patients with CRC.^45,75^

In clinical CRC specimens, CX3CR1, the chemoattractant cytokine CX3CL1 receptor, was expressed on TAMs and in a microenvironment lacking CX3CR1, the liver metastasis of CRC cells was significantly inhibited.^76^ However, the high expression of CX3CR1 promoted the increase of T cell invasion in tumor tissue, and thus inhibited tumor growth.^77^ Low-molecular-weight heparin, a common drug for venous thromboembolism, inhibited the formation of liver metastasis of CRC by disrupting the interaction of CXCR4 and CXCL12.^78^ Stromal cell-derived factor 1alpha activated CXCR4-expressing CRC cells, then resulted in a significant increase of cell migration.^79^ Moreover, hepatic stellate cells (HSCs) play important role in liver metastasis of CRC cells by the action of SDF-1/CXCR4 axis.^79^

### The emerging roles of exosomes in CRLM

**Fig. 2:**
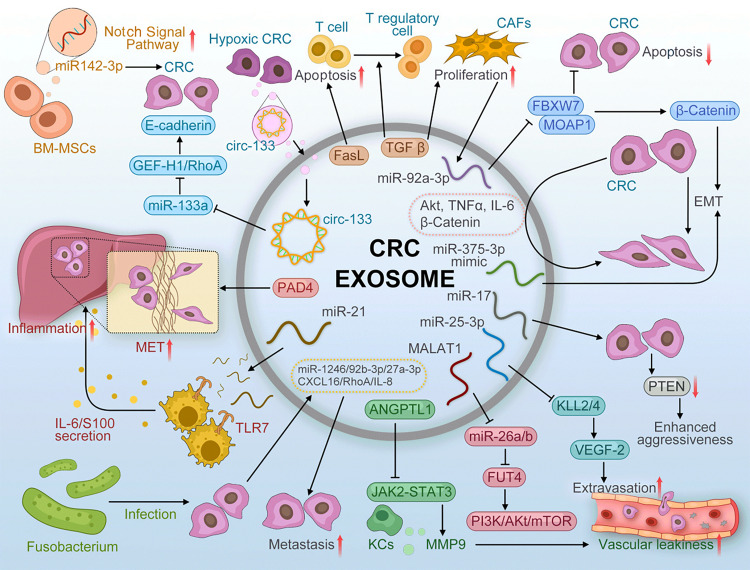
CRC-derived exosomes content and mechanisms for CRLM

**Fig. 3:**
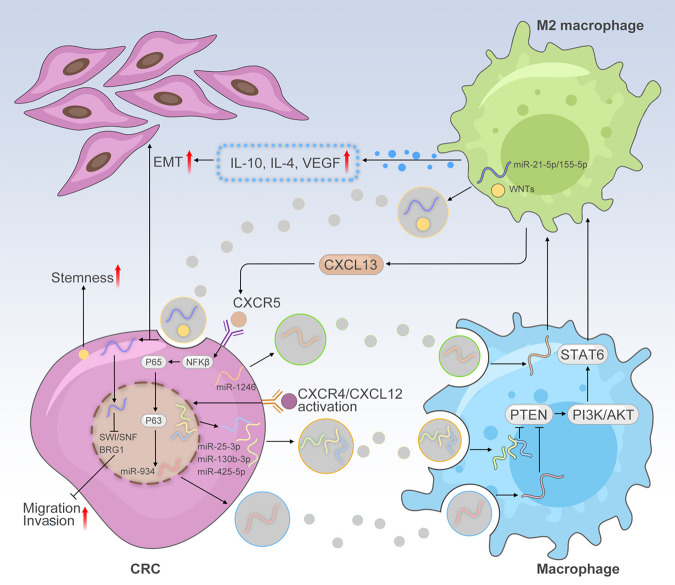
Schematic illustration the exosomal interaction between CRC cells and TAMs that reveals the molecular mechanism of CRLM

Like chemokine, exosomes also built crosstalk between various cells. Exosomes are lipid bilayer vesicles with a diameter of 30–100 nm.^80^ Exosomes are very widespread and are found in almost all body fluids.^81^ Cancer patients have more circulating exosomes than non-cancer patients.^82^ Exosomes can also mediate colonic epithelial-stromal interactions involved in the regulation of tumor growth and metastatic invasion.^83^ Exosomes play a key role in building a supportive microenvironment in the metastatic organs, namely the premetastatic niches (PMNs), which include vascular leakiness, inflammation, and immunosuppression.^84^ We review the role of exosomes in CRLM development, and the clinical application of exosomes (Figs. 2 and 3).

CRC-derived exosomes contain various biomolecules such as protein (PAD4, FasL, TGFβ, angiopoietin-like protein 1 (ANGPTL1), AKt, tumor necrosis factor-α (TNF-α), β-catenin), RNA (miR-246, miR-21, miR-25-3p, miR-375-3p, miR-92b-3p, miR-27a-3p, miR-17, MALAT1). These biomolecules establish PMNs in liver and induce proliferation, inflammation, EMT, invasion, migration and EMT, thereby promoting CRC metastasis.

The Complicated network of the exosomal interactions among the CRC cells and TAMs promotes metastasis. CRC induces macrophages to M2 macrophages in TME via secreting exosomal miRNA (miR-934, miR-25-3p, miR-130-3p, miR-425-5p and miR-1246). M2 macrophages also promoted CRC EMT and maintained stemness by exosomal miR-21-5p and WNTs. Moreover, CXCR5, IL-10, IL-4 and VEGF were also the medium of communication between CRC cells and TAMs.

#### The substances in exosomes

The substance in exosomes includes proteins, miRNAs, lncRNAs and mRNAs, which can spread in the circulatory system and play an important role in CRLM. ANGPTL1, a tumor suppressor, was decreased in CRC tissue.^85^ Exosomal ANGPTL1 decrease MMP9 production in Kupffer cell by suppressing the JAK2-STAT3 signaling pathway.^86^ Tumor cells can transfer EMT inducers, such as IL-6, Akt and TNF-α, through exosomes to induce EMT in neighboring tumor cells.^87^

In addition to protein, miRNAs are also a common substance in exosomes. Circulating exosomal miRNA can be used to assist in the diagnosis or prognosis evaluation of CRC patients. Circulating miRNAs are mainly derived from exosomes miRNA.^88^ Therefore, the analysis of circulating exosomal miRNAs can promote the early metaphase diagnosis rate of CRLM and intervention in the metastatic process. Zeng et al.^89^ found that exosomal miR-25-3p secreted by CRC cells was transferred to vascular endothelial cells and promoted CRC metastasis by targeting Kruppel-like factor 2 and Krüppel-like factor 4 (KLF4) to increase vascular permeability and promote angiogenesis in animal models. The clinical data further showed that miR-25-3p from circulating CRC-derived exosomal miR-25-3p can be used as a biomarker to predict metastasis. Similarly, CRC-derived exosomes packed with miR-21-5p created a liver pro-inflammatory phenotype and liver metastasis of CRC via the miR-21-Toll like receptor 7-IL-6 axis.^90^ Metastasis-associated miR-106b-3p from serum exosomes targeted deleted in liver cancer-1.^91^ Elisabetta et al.^92^ reported that exosomal miR-210 can perhaps be reckoned as an EMT signal promoter which maintain the local cancer-growth milieu and influence the adhesion and migration of CRC cells. Moreover, Matsumura et al.^93^ showed that six exosomal miRNAs (miR-19a, miR-19b, miR-4437, miR-23a, miR-320a and miR-92a) were related to the liver metastasis. Interestingly, miR-375 mimicThe tumor-derived exosome containing miR-375 mimic that could inhibit the EMT process.^94^ According to Fu et al.^95^ the high expression of miR-17-5p and miR-92a-3p were consistent with the tumorigenesis and metastasis of CRC by exploring serum exosomal miRNAs levels of normal controls and CRC patients. Interestingly, exosomal miR-1246/92b-3p/27a-3p derived by fusobacterium nucleatum-infected CRC cells facilitate uninfected cells metastasis.^96^ Recently, Zhang et al.^97^ reported the secretion of CRC exosome miR-1255b-5p was decreased under hypoxia, thereby promoting human telomerase reverse transcriptase inhibition to enhance EMT and telomerase activity.

Moreover, exosomal miRNAs are secreted by cancer-associated fibroblasts (CAFs), then they are transferred to CRC cells.^98^ Mechanistically, miR-92a-3p inhibits mitochondrial apoptosis by activating the Wnt/β-catenin pathway and inhibiting FBXW7 and MOAP1, thereby enhancing stemness, EMT, metastasis and 5-FU/L-OHP resistance of CRC cells.^98^ Reducing exosomal miR-92a-3p may contribute to the prediction and treatment of CRLM. CRC cells can also induce CAF via exosomal tumor growth factor-β (TGF-β).^99,100^ Moreover, exosomes miR-142-3p secreted by bone marrow-derived mesenchymal stem/stromal cells inhibited Numb expression in CRC cells, thereby increasing the population of CSCs.^101^

MiRNAs in exosomes isolated from CRLM are different from the profile generated from an orthotopic cecum tumor model and naïve colon tissues because higher levels of tumor suppressor miRNAs are encapsulated in the exosomes in more advanced disease.^102^ The level of oncogenic miR-21 was much higher in the primary colon tumor tissue and metastatic colon tumor in the liver than in their exosomes. However, the level of tumor suppressive miR-18a and miR-193a in the exosomes is enhanced.^102^ The serum levels of exosomal miR-200c and miR-141 were proved to predict CRC patients with poor prognosis.^103^ The monitoring of CRC metastasis was monitored in real time based on miR-139-3p content in the plasma of CRC patients.^104^ Similarly, in CRC patients with LM, the serum exosomal miR-122 can serve potentially as both a novel diagnostic and prognostic biomarker.^105^ A recent study provides a new concept that miR-25-3p, miR-130b-3p, miR-425-5p, miR-193a, let-7g,miR-106b-3pand miR-934 contained in exosomes could participate in progression and metastasis of CRC.^91,106–108^ Thus, all the evidences point out that exosome-mediated promotion of tumor progression, and understanding these mechanism-based circulating exosomal miRNAs will reveal new avenues for future diagnosis and treatment.

Furthermore, the exosome-derived lncRNAs could play essential roles in tumorigenesis by regulating the TME.^109^ Exosomal lncRNAs BCAR4 could be potential candidates to detect CRC.^110^ Exosomal MALAT1 regulated fucosyltransferase 4 (FUT4) expression by sponging miR-26a/26b to promote CRC progress.^111^ CRC cells secreted exosome cirC-133 into relatively normoxic CRC cells under hypoxic conditions. Then circ-133 adsorbed miR-133a to target GEF-H1/RhoA, as a result of reducing the distribution of E-cadherin on the membrane.^112^ Guo et al.^96^ reported fusobacterium infection may stimulate CRC cells to generate CXCL16/RhoA/IL-8 exosomes that are delivered to uninfected cells to promote prometastatic behaviors.

#### The exosomal interaction between CRC cells and TAMs

There is also a noticeable interaction between CRC cells and TAMs via exosomes.^113^ Exosomes secreted by M2 macrophages promoted CRC migration by exosomal transfer of miR-21-5p and miR-155-5p.^114^ Mechanistically, these miRNAs mentioned above targeted the core component of the Switch/sucrose non-fermentable complex BRG1. Moreover, M2 macrophages -derived exosomes could induce CRC stem cell activity because of containing WNT.^115^ CRC cell-derived exosomal miR-934 induced M2 macrophage polarization via downregulating PTEN expression and activating the PI3K/AKT signaling pathway.^107^ Polarized M2 macrophages secreted CXCL13, then activated a CXCL13/CXCR5/NFκB/p65/miR-934 positive feedback loop to induce PMN formation in CRC cells. Exosomal miRNAs miR-25-3p, miR-130b-3p, miR-425-5p secreted by CRC cells via activation of the CXCL12/CXCR4 axis, could be transferred to macrophages which can then target PTEN and shift the macrophage towards M2 phenotype.^106^ Additionally, mutant p53 CRC could reprogram macrophages into M2 macrophages by transferring exosomal miR-1246.^116^

#### The exosomal interaction between CRC cells and other types of immune cells

CRC-exosomes containing transferring TGF-β can activate TGF-β/Smad signaling and inactivate SAPK signaling which induces phenotypic alteration of T cells to Treg-like cells.^117–119^ Delivery of Fas-ligand-containing CRC cell exosomes to T cells can induce cell apoptosis.^120^ Citrullination generated by peptidylarginine deiminase 4 (PAD4) derived from CRC cells as a driver of liver metastases.^121^ In another study, it was reported that mutated KRAS is transferred to recipient cells through exosomes and induces increased IL-8 production, recruiting more neutrophils and further aggravating CRC.^122^

### Key molecules and signaling pathway driving liver metastasis

Various signaling pathways and factors may be involved in the process of CRLM, including hepatocyte growth factor/c‐Met (HGF/c-Met) signaling pathway, phosphatase of regenerating liver (PRL3), Notch pathway, TGFβ signaling, Tyrosine kinase c-MET signaling, tumor-associated calcium signal transducer 2 (Trop-2), L1 cell adhesion molecule (L1CAM), metastasis-related gene 1 (MACC1), S100 family proteins and other pathways. There are multiple intersections between these molecular mechanisms driving CRLM.

#### HGF/c-Met signaling pathway

**Fig. 4:**
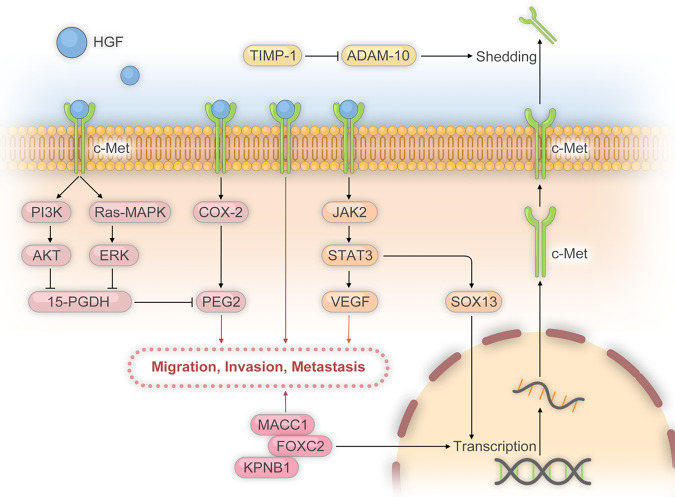
HGF/c‐MET signaling pathways and its role in cellular activity

HGF/c-Met signaling pathway promotes metastasis of cancer cells by regulating a diverse downstream prometastatic effector molecules, then overactivated phosphatidylinositol-3-kinase (PI3K) and mitogen-activated protein kinase (MAPK) signaling (Fig. 4).^123–125^ Met, a receptor of hepatocyte growth factor (HGF), has a positive correlation with tumor stages of CRC liver metastasis. High expression of circulating HGF and aberrant activity of cMet were detected in patients with CRC.^126^ Inhibition of HGF/C-MET signaling pathway could decrease the proliferation and invasion of CRLM.^127^ By assessing the patient tissue samples, Yao et al.^128^ reported higher levels of c-Met expression (mRNA and protein) in CRLM than primary CRC by assessing the patient tissue samples. HGF/c-Met could regulate urokinase plasminogen activator (uPA) and uPAR in vitro CRC migration and invasion models.^129^ Ectopic TIMP1 expression provoked prometastatic microenvironment in the liver by inducing the HGF/c-Met signaling as well as uPA expression.^130,131^ Inhibition of ADAM-10 was in principle able to prevent shedding of c-Met, which may be one explanation for the increase of cell-associated c-Met in livers with elevated TIMP-1.^130^ Indeed, in livers of uPA-ablated mice upregulated TIMP-1 expression did not trigger HGF/c-Met signaling.^131^ Moreover, elevated levels of TIMP-1 in the TME could induce metastasis by enhancing HIF-1α-dependent HGF-signaling.^132^

In addition, SOX13 is induced by HGF through JAK2/STAT3 signaling and then upregulated SOX13 transactivates the expression of c-MET by directly binding to its promoters, which contributes to c-MET overexpression in CRC.^133^ This feedback loop induces SOX13-mediated CRC migration, invasion, and metastasis. Similar to SOX13, Forkhead box protein C2 (FOXC2) was directly associated with c-MET promoter to increase the transcriptional activity of MET.^134^ Blocking KPNB1 (a novel gene) expression showed a significant inhibitory role in metastasis both in vitro and in vivo through interacting with MET proto-oncogene. COX-2/PGE(2) pathway as an important mediator of HGF/Met signaling is closely associated with the survival, proliferation and invasion of CRC cells.^135^ HGF/Met signaling is an important regulator of the COX-2/PGE 2 pathway in CRC cells, stimulating PGE 2 synthesis via COX-2 upregulation and inhibiting PGE 2 degradation via Ras-MAPK/ERK while PI3K/AKT signaling mediate HGF-driven 15-PGDH downregulation.^136^ In fact, IL-6/IL-6R, HGF/c-Met, STAT3, VEGF cytokinetic pathway is a major mechanism of the pro-oncogenic effect induced by hepatic RFA.^137^

The association between C-Met and CRC was even stronger due to c-MET being identified as a transcriptional target of colon cancer MACC1.^138^ MACC1 promotes proliferation, invasion, and HGF-induced scattering of CRC cells in cell culture and tumor growth and metastasis in mouse models. In advanced metastatic CRC patients, MACC1 and c-Met were both upregulated.^138^ MACC1, a new detectable biomarker in cancer, is also an independent prognostic factor for the recurrence after liver resection of CRC metastasis.^139^

HGF/c-Met signaling promoted metastasis of cancer cells by regulating a diverse downstream prometastatic effector molecules, via Ras-MAPK/ERK, PI3K/AKT signaling, JAK2/STAT3 signaling. MACC1, KPNB1 and FOXC2 could transactivate the expression of c-MET.

#### CRLM and PRL3

**Fig. 5:**
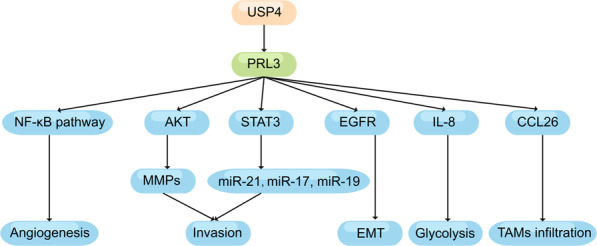
The roles of PRL3 in cellular activity

PRL3 has received widespread attention as a potential cause for metastasis (Fig. 5).^140^ Since PRL3 transcripts are overexpressed in CRC metastases found in the liver, and PRL3 expression cannot be detected in non-metastatic primary tumors and normal colorectal epithelium. PRL3-induced enhancement of EMT dependent on EGFR activation and PRL3 promoted cell invasion and upregulated MMPS by activating AKT in vitro and in vivo.^141–144^ The levels of PRL-3 mRNA expression can be used as biomarkers for increased risk of liver metastasis.^145^ The PRL-3 expression did not represent a direct causative mechanism of liver metastasis, but modulated multiple signaling pathways, including PI3K/AKT and MAPK/ERK in various cancer cells. It was reported liver metastasis by PRL-3 is mediated through lymph node metastasis and elevated tumor markers (CEA and CA19-9) in the serum.^146^ Overexpression of PRL-3 can also promote the proliferation and invasiveness of CRC cells, mainly by activating STAT3 to increase the expression of miR-17, miR-19a and miR-21.^147^ There is evidence that ubiquitin-specific protease 4 drives CRC invasion and metastasis via binding with and deubiquitinating PRL-3 to stabilize PRL-3.^148^ Moreover, PRL-3 improved IL-8 secretion in CRC cells and mediated enhancement of glycolysis, which in turn contributed to the promotion of cancer metastasis.^149^ PRL-3 also promoted CRC invasion and metastasis by upregulating chemokine ligand 26 (CCL26) to induce cancer metastasis.^150^ Recently, it was shown that PRL-3 promoted cell metastasis via activating MAPK pathways in TAMs to initiate EMT, and the NF-κB pathway activated by PRL-3 contributed to angiogenesis in CRC cells.^151^

PRL3 promoted metastasis of cancer cells by regulating a diverse downstream prometastatic effector molecule, via NF-κB pathway, AKT, STAT3, EGFR, IL-8 and CCL26.

#### CRLM and Notch pathway

**Fig. 6:**
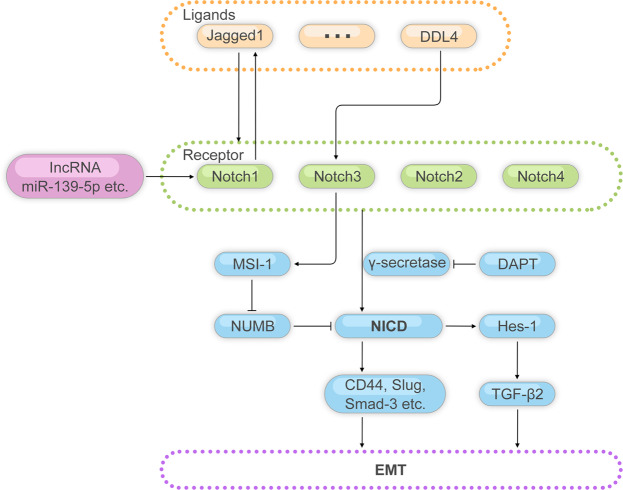
Schematic illustration of Notch pathway regulatory mechanisms

Furthermore, there are other signaling pathways that are related to CRLM. The Notch pathway plays a positive role in CRLM and inhibition of Notch signaling interferes with CRLM.^122^ The human Notch system includes four Notch receptors (Notch1-4) and five Notch ligands (Jagged1-2, DLL1, DLL3, DLL4).^152^ Activation of the Notch pathway is related to the poor prognosis of CRC. Notch1 gene copy number amplification may indicate a decrease in patient survival.^153^ The positive expression of Notch3 protein is an unfavorable prognostic factor of disease-free survival (DFS) and overall survival (OS).^154^ However, the functions of notch2 and notch4 are opposite to them, their overexpression can block the proliferation, invasion or migration of cancer cells.^155,156^ Meanwhile, multiple Notch signaling pathway related mechanisms play a significant role in metastatic CRC(Fig. 6)

The enhanced aggressiveness of colorectal tumors caused by Notch signal activation may be related to EMT. Retroviruses are used to construct colorectal tumor cells that can stably express Notch-1 intracellular domain (NICD-1) in the cytoplasm, and the Notch ligand Jagged-1 and CD44, Slug, Smad-3 and other EMT-related proteins are found to be upregulated in these CRC cells.^157^ Further treatment of the cells with γ-secretase inhibitor DAPT can effectively inhibit this process. Interestingly, NICD-1 produced by retroviruses is not affected by DAPT, which suggests that this may be caused by a more complicated mechanism-after the activated Notch1 receptor releases NICD, the expression of the ligand Jagged1 is upregulated. Animal experiments have shown that when Notch/TGF-β2 signaling is inhibited, it is accompanied by a decrease in neutrophils in the target organs of metastasis, as well as the gradual accumulation of CD3+, CD4+ and CD8+ T cells, resulting in a significant reduction in metastasis.^158^ In addition, in the EMT-related literature of other types of cancer, it has also been reported that hey-1, an important downstream target gene of the Notch signaling pathway, can regulate the TGF-β/Smad signaling pathway, and the TGF-β-dependent signaling pathway is closely related to the induction of EMT phenotypes.^159^ This indicates that the crosstalk of these two signaling pathways may play a more essential role in the process of colorectal tumor metastasis.

Song et al.^160^ confirmed that autocrine motor factor receptor (AMFR) and notch1 are the direct target genes of miR-139-5p in colorectal tumors, upregulation of miR-139-5p promotes the expression of AMFR and NOTCH1 to enhance the migration and metastasis of CRC. In addition, knockdown of Notch ligand Jagged1 can lead to a decrease in Notch signal transduction activity of CRC cells, thereby reducing the migration of CRC cells. Moreover, in Jagged1 silenced nude mouse xenograft tumors, tumor metastasis markers MMP-2 and MM-9 are also significantly downregulated.^161^ Ligand DLL4 can upregulate MUSASHI-1 (MSI-1) through a Notch3-mediated mechanism, while MSI-1 inhibits the translation of Notch signaling negative regulator NUMB, and then reduces the degradation of NICD in tumor cells, and promotes the activation of Notch signaling pathway.^162^ In addition, Amino-terminal Enhancer of Split (AES) can inhibit the metastasis of colorectal tumors by lowering Notch signal.^163^ In the past, there was little knowledge about the regulation of AES expression. The latest research found that it may be affected by the human Casein kinase 1δ/ε (CK1δ/ε), meanwhile CK1δ/ε is closely related to Wnt signal and Hedgehog signal in the occurrence and development of CRC.^164^

Ectopic expression of tRNA-derived fragments (tRF)/miR-1280 reduced cell proliferation and metastasis by directly inhibiting the Notch signaling via targeting JAG2, leading to the decreased activity of Notch pathway components and Notch signaling.^165^ Moreover, the intensity of Notch1 expression was related to depth of invasion, tumor node metastasis (TNM) staging and lymph node metastasis of CRC.^166^ Inhibition of tumor-derived Tumor-Derived Laminin α5 (LAMA5) activated the Notch pathway in tumor endothelial cells, thereby reducing branching angiogenesis.^167^

MSI-1, MUSASHI-1; CK1δ/ε, Casein kinase 1δ/ε; NICD, Notch intracellular domain; EMT, epithelial-to-mesenchymal transition.

#### CRLM and TGFβ signaling

The TGF-β superfamily signaling contains more than 30 members, among which the more important ones are TGF-β, Activins, Nodal, Bone Morphogenetic Proteins, etc.^168^ In mice with progressive liver metastatic disease, blockade of TGFβ signaling rendered tumors susceptible to anti-programmed cell death 1 (PD1) therapy.^169^ TGF-β1 could also downregulate the E-cadherin expression and increase the Vimentin expression, inducing EMT to promote the invasion and migration of CRC.^170^ Moreover, CRC-derived CXCR4 activated HSCs to release SDF-1, resulting in TGF-β1 secretion in CRC cells to promote liver metastasis of CRC.^171^ Therefore, TGF-β1 signaling blockage may be an effective clinical strategy for CRLM.

**Fig. 7:**
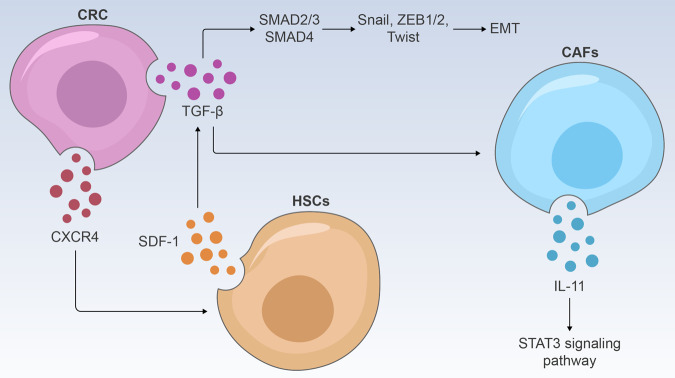
Schematic illustration of TGF-β pathway regulatory mechanisms

TGF-β plays a dual role in the tumorigenesis process as illustrated in Fig. 7. In the early stage of tumorigenesis, TGF-β plays a role in inhibiting tumors. However, in the later stages, TGF-β can promote EMT and is closely related to tumor invasion and metastasis.^172^ In CRC, CMS4 type has a higher degree of TGF-β activation.^173^ Studies have shown that TGF-β1 can promote the occurrence of EMT by activating a variety of transcriptional regulators including SNAI1/2, Twist and ZEB1/2, etc.^174^ Increased expression of TGF-β1 can increase the metastasis of CRC cells, leading to the secretion of IL-11 by CAFs to trigger the STAT3 signaling pathway.^175^

Equally important, SMAD4 is also an essential molecule in the TGF-β signaling pathway. It has been reported that up to 62% of CRC patients with liver metastases have SMDA4 downregulation^176^, and the loss of SMAD4 has been shown to cause abnormal activation of STAT3 in pancreatic cancer cells, which then makes the expression of E-cadherin decrease, N-cadherin and vimentin increase, leading to the occurrence of EMT.^177^ By constructing gene-virus CD55-Smad4 and overexpressing SMAD4, Xiao et al. confirmed that the metastasis and tumor cell stemness of CRC cells can be effectively inhibited by activating the Wnt/β-catenin signaling pathway.^178^ In addition, there are a significant amount of studies confirming that some ncRNAs, affect the invasion and metastasis of CRC by regulating the TGF-β/Smad axis. For instance, LINC00941 promotes CRC metastasis via activation of the TGF-β/SMAD2/3 axis^179^, and lncRNA ANRIL promotes CRC metastasis through activating the let-7a/TGF- β 1/Smad Axis.^180^

CXCR4 secreted by CRC cells promoted SDF-1 secretion by HSCs. SDF-1 in turn acted on CRC and promoted its secretion of TGF-β. TGF-β can not only promote the secretion of IL-11 by CAFs to activate the STAT3 signaling pathway, but also further regulated gene transcription through SMAD in CRC cells and promoted the occurrence of EMT.

#### Other molecular targets and pathways involved in CRLM

L1CAM is a marker and mediator of metastasis initiating cells, which are required for orthotopic carcinoma propagation, liver metastatic colonization and chemoresistance.^181,182^ L1CAM is induced after loss of epithelial integrity, it then promotes tumor growth and metastasis.^183^ The mechanism that epithelial cells enter into an L1CAM high phenotypic state is epithelial dissociation causes transcriptional downregulation of REST, in turn reducing REST occupancy of an L1CAM intronic enhancer and promoting L1CAM expression. Research and development of L1CAM inhibitory molecules-as a treatment of diffuse residual disease and metastatic disease is of great significance.^183^ The scattered Lgr5 low and L1CAM high cells in the primary tumor can trigger the distant metastasis of CRC, but will dynamically emerge from the Lgr5 high primary tumor when the epithelial integrity is lost during the self-destructive tumor infiltration process, which is a distant metastatic organ necessary for the survival and re-growth of diffuse cancer cells. Future studies may elucidate the close relationship between wound healing and metastasis.

Akt2 is one of the three subtypes in the Akt family, as it promotes cell movement/invasion.^184,185^ Akt2 is highly expressed in stage IV CRCs and liver metastases, meanwhile, loss of PTEN and overexpression of Akt2 synergistically promote metastasis.^186^ The inducible loss of Akt2 in CRC cells strongly upregulates metastasis Suppressor 1 (MTSS1) at the messenger RNA and protein levels.^187^ MTSS1 is a new gene regulated by Akt2, where the inhibition of MTSS1 is a key step for Akt2 to promote metastasis in CRC cells. Moreover, Elevated eukaryotic translation initiation factor 4A (EIF4A) in CRC patients was associated with poorer survival rate, poor response to oxaliplatin and more distant metastasis.^188^ The EIF4A inhibitor silvestrol and oxaliplatin have a synergistic effect, and the combined use of the two may represent a new treatment strategy for patients with CRLM. The Trop-2 expression is another necessary for tumorigenesis and invasiveness of CRC cells.^189^ Interestingly, ZFP57, an embryonic stem cell-specific transcription factor has been shown to promote liver metastasis of CRC.^190^ Tang et al.^191^ found phosphoprotein enriched in astrocytes-15 kDa (PEA15) was highly expressed in CRLM patients compared to non-metastatic. PEA15 promote CRC proliferation and the abilities of invasion and migration though activating the ERK/MAPK signaling pathway. PEA15 and ZFP57 may be a potential therapy biomarker for CRLM.

There are various epidermal growth factor-like domain protein 6 (MEGF6) in CRC, which induce EMT via transforming growth factor β (TGFβ)/SMAD signaling pathway to promote the transfer of CRC.^192^ Through the analysis of expression profile microarray data, it is concluded that apolipoprotein E (ApoE) expression in normal mucosal tissues, primary CRC and CRLM increased sequentially.^193^ The overexpression of ApoE is related to the progression of CRC, especially for stage II and simultaneous liver metastases, resulting in a poor prognosis for CRC patients. Moreover, lgG Fc binding protein RNA and protein were significantly downregulated in metastatic lesions, which were associated with the prognosis of CRLM.^194^ In addition, two members of the S100 gene family, S100A6 and S100A4, are thought to be involved in the invasion and metastasis of cancer. When cancer cells form a glandular structure again in the center of metastatic nodules, the expression level of S100A6 decreases.^195,196^ Knockdown of S100A4 restricts metastasis formation in a xenografted mouse model of CRC.^197^ Moreover, S100A8, another member of the S100 family is correlated with TIMP-1-induced PMN in liver.^198^

A new member of the F-box protein family, FBX8, containing F-box and Sec 7 domain, can ubiquitinate and degrade HIF-1α such as CDK4 and C-Myc, downregulating their ability for promotion of angiogenesis, cycle progression and cell proliferation, respectively, thus regulating the CRC liver metastasis dormancy.^199^ Moreover, Thrombospondin-1 (THBS1) depletion inhibited migration and invasion of CRC cells through attenuating EMT.^200^ Upregulated THBS1 may be significantly correlated with CRLM, which requires further study.

Hypermethylated B4GALT1 was detected in the mCRC case group and had adverse prognostic effects on CRC.^201^ Glyco-genes *B4GALT1* may act as an additional novel biomarker for CRLM. Moreover, Ferritin Light Chain (FTL) as an oncogene leads to CRC cell resistance against 5-FU treatment and promoted CRC metastasis by competing with lncRNA Linc00467 for miR-133b binding site.^202^ The expression of defensin β 4 A (*DEFB4A*) was significantly upregulated and experimentally proved that DEFB4A gene knockdown proved that DEF4BA promoted cell migration.^203^ Sialyltransferase ST6GAL1 can increase the stability of ICAM-1 through sialylation, thereby inhibiting the transfer characteristics of CRC.^204^

Under hypoxic conditions, loss of the tumor suppressor p53 (encoded by TP53) provides cancer cells with a selective advantage, hypoxia which causes resistance to therapy and promotes metastasis in CRC.^205,206^ Recently, Li et al. reported the hypoxia inducible factor 1 alpha subunit (HIF1A) directly repressed the miRNA-34a in p53-defective CRC cells under hypoxia.^207^ Conversely, p53 increases expression of miRNA-34a in CRC cells without hypoxia. Inhibition of Protein phosphatase 1 regulatory inhibitor subunit 11 (PPP1R11) by miRNA-34a prevented activation of STAT3 and inhibited the hypoxia-induced EMT and metastasis. Similarly, upregulated PPP1R11 was associated with TP53 mutations and metastasis to the liver. Targeting this pathway may represent a therapeutic opportunity for managing metastatic disease. The NF-κB/p65 signaling pathway has a critical role in the mediation of GNA13 in CRC which was indicated by a decrease in GNA13-induced migration, invasion, and in addition, CXCL chemokine level increases after the inhibition of NF-κB/p65 signaling pathway with an inhibitor.^70^ The novel quinazoline derivative MJ-56 interfered with the NF-κB signaling via impairing PI3K/AKT activation and subsequently reduced the NF-κB-mediated transcription of MMPs.^208^

Chu et al.^209^ reported that mutant KRAS transcriptionally activates IGF-IR gene expression through Y-box-binding protein (YB)-1 upregulation via a novel MEK-Sp1-DNMT1-miR-137 pathway in CRC cells. Moreover, suppression of the expression of YB-1 and IGF-IR via inhibition of MEK hampers KRAS-driven CRLM in animal model studies. KRAS-driven MEK-Sp1-DNMT1-miR-137-YB-1-IGF-IR signaling pathway, which might provide a mechanistic rationale for the use of a MEK inhibitor as an adjuvant, in combination with standard of care, to prevent the recurrence of CRLM in KRAS mutant CRC patients after receiving liver resection, however, further investigation is needed. Analysis of the TCGA database confirmed the upregulation of retinoblastoma binding protein 4 (RBBP4) in CRC tissues, and the overexpression of RBBP4 is related to nerve infiltration and poor chemotherapy effects.^210^

### NcRNAs in CRLM

NcRNAs include long non-coding RNA (lncRNAs), microRNAs (miRNAs) and circular RNAs (circRNAs), which are emerging as the master regulators of cancer. The dysregulated expression NcRNAs have coding-independent functions in the progression of CRLM. In this section, we discuss some lncRNA and their role in CRLM (Tables 2, 3), circRNAs in CRLM (Table 4), miRNAs in the regulation of cascade of CRLM (Table 5).

**Table 2:** LncRNA in CRLM

LncRNA	Role	Function	Refs.
CLMAT3	Promote	Metastasis	^217^
SNHG15	Promote	Metastasis	^218^
TPT1-AS1	Promote	Angiogenesis, metastasis	^216^
ENSG00000274093.1	Promote	EMT	^215^
HOTAIR	Promote	Migration, invasion, EMT	^214^
LALC	Promote	Metastasis	^211^
LOC441461	Promote	Cell growth and motility	^424^
SATB2-AS1	Suppress	Cell growth and metastasis	^219^
MIR22HG	Suppress	Metastasis, EMT	^220^

**Table 3:** LncRNA/miRNA/mRNA ceRNA network in CRLM

LncRNA	Role	Function	Shared miRNA	Competitor mRNA	Refs.
H19	Promote	EMT progression	miR-138, miR-200a	Vimentin, ZEBl, ZEB2	^425^
TP73-AS1	Promote	Proliferation, migration, and invasion	miR-194	TGFα	^227^
MIR4435-2HG	Promote	Proliferation, metastasis	miR-28-5p	YAP1	^224^
UICLM	Promote	Metastasis	miR-215	ZEB2	^221^
RP11-757G1.5	Promote	Proliferation, metastasis	miR-139-5p	YAP1	^217^
ZEB1-AS1	Promote	Cell growth and metastasis	miR-455-3p	PAK2	^230^
FARSA-AS1	Promote	Stemness, metastasis	miR-18b-5p, miR-28-5p	SOX9, FARSA	^231^
HSD17B11-1:1	Promote	Proliferation, mobility, and invasion	miR-338-3p	MACC1	^232^
SNHG7	Promote	EMT, metastasis	miR-216b	GALNT1	^426^
SNHG14	Promote	Metastasis	miR-186-5p	EZH2	^427^
DANCR	Promote	Proliferation, metastasis	miR-577	HSP27	^226^
MIR4435-2HG	Promote	Proliferation, metastasis	miR-206	YAP1	^224^
Plncrna-1	Promote	Proliferation, metastasis	miR-204	Wnt/p-catenin	^225^
MALAT1	Promote	Metastasis	miR-106b-5p	SLAIN2	^428^
HOTAIR	Promote	Metastasis	miR-214	ST6GAL1	^223^
GAPLINC	Promote	Migration, invasion	miR-34a	c-MET	^229^
B3GALT5-AS1	Promote	EMT, metastasis	miR-203	ZEB2, SNAI2	^233^
LINC00485	Suppress	Cell growth, metastasis	miR-581	EDEM1	^429^

**Table 4:** CircRNA associated with the development of CRLM

CircRNA	Role	Function	Refs.
Has_circ_0071589	Promote	Cell growth, invasion, and migration	^239^
hsa_circ_0001178	Promote	Invasion, metastasis	^244^
circ_0124554	Promote	Metastasis	^237^
hsa_circ_102049	Promote	Migration, invasion, metastasis	^243^
Hsa_circ_000984	Promote	Metastasis	^238^
hsa_circRNA_102209	Promote	Cell growth, metastasis	^430^
CircRNA_0001178	Promote	Metastasis	^244^
CircRNA_0000826	Promote	Metastasis	^249^
Circ-NSD2	Promote	Metastasis	^241^
circAPLP2	Promote	Proliferation, metastasis	^240^
CircPPP1R12A	Promote	Proliferation, migration and invasion	^235^
CircCCDC66	Promote	Cell growth, metastasis	^242^
CircRNA NSUN2	Promote	Metastasis	^236^
circ_0115744	Promote	Metastasis	^431^
CircITGA7	Suppress	Proliferation, metastasis	^247^
CircRNA-FNDC3B	Suppress	Metastasis, invasion, and angiogenesis	^246^
Hsa_circ_0009361	Suppress	Cell growth, metastasis	^245^

**Table 5:** MiRNAs in CRLM

MiRNA	Role	Function	Molecular target	Refs.
MiR-15b	Promote	Invasion, metastasis	MTSS1, Klotho	^274^
MiR-30b	Promote	EMT, metastasis	SIX1	^255^
MiR-19	Promote	Invasion, metastasis	TG2, STAT3	^147,268^
MiR-17	Promote	Metastasis	STAT3	^147^
MiR-21	Promote	Invasion, intravasation, metastasis	STAT3, Pdcd4	^147^
MiR-103/107	Promote	Colonization, metastasis	DAPK, KLF4	^302^
MiR-181a	Promote	EMT	WIF-1	^254^
MiR-885-5p	Promote	Migration, invasion	Cpeb 2	^277^
MiR-20a-5p	Promote	Invasion, metastasis	Smade	^272^
MiR-429	Promote	Apoptosis	SOX2	^253^
MiR-298	Promote	Invasion	PTEN	^273^
Let-7	Promote	EMT, metastasis	HMGA2	^263^
MiR-21	Promote	Migration, invasion	Pdcd4	^269^
MiR-497	Promote	Metastasis	Fra-1, VEGF-A	^281^
MiR-10b	Promote	EMT, metastasis	KLF4	^266^
MiR-10a	Suppress	EMT, metastasis	MMPI4, ACTG1	^265^
MiR-200	Suppress	EMT	ZEB1/2, ETS1, FLT1	^252^
MiR-212	Suppress	EMT, metastasis	MnSOD	^257^
MiR-30a	Suppress	EMT	TM4SF1, VEGF, E-cadherin	^256^
MiR-31	Suppress	Metastasis	E-selectin	^301^
MiR-26a	Suppress	Metastasis	FUT4	^289^
MiR-26b	Suppress	Invasion, metastasis	FUT4	^289^
MiR-551a	Suppress	Colonization, metastasis	–	^303^
MiR-483	Suppress	Colonization, metastasis	–	^303^
MiR-195	Suppress	Angiogenesis	VEGF	^432^
MiR-99b-5p	Suppress	Migration	mTOR	^285^
MiR-29	Suppress	Migration, invasion	MMP2	^433^
MiR-214	Suppress	Migration, invasion	FGFR1	^287^
MiR-30e-5p	Suppress	Proliferation, metastasis	ITGA6, ITGB1	^290^
MiR-196b-5p	Suppress	Migration, invasion	HOXB7, GALNT5	^292^
MiR-34a	Suppress	Migration, invasion, EMT	IL-6R	^56^
MiR-365a-3p	Suppress	Migration, invasion, EMT	ADAM10	^295^
MiR-487b	Suppress	Metastasis	LRP6	^296^
MiR-186-5p	Suppress	EMT	ZEBl	^259^
MiR-15a/16-1	Suppress	EMT	AP4	^262^
MiR-132	Suppress	Invasion	ANO1	^298^
MiR-17-5p	Suppress	EMT	vimentin	^260^
MiR-146a	Suppress	Metastasis	c-Met	^299^
MiR-143-3p	Suppress	Metastasis	ITGA6 and ASAP3	^297^
MiR-328-3p	Suppress	Metastasis	Girdin	^294^

#### LncRNAs in CRLM

Recently, Zhang et al.^211^ demonstrated that lncRNA LALC recruited DNA methyltransferases (DNMTs) to the LZTS1 promoter by coupling with EZH2 and then modified the expression of LZTS1 through DNMTs-mediated DNA methylation in CRLM. Methyltransferase like 3 behaves as the ‘writer’ of m6A promoted m6A-methylation of oncogenic lncRNA XIST to inhibit CRC proliferation and invasion.^212^ LncRNA LINC01578 activity was enhanced when nuclear factor kappa B (NF-κB) and Yin Yang 1 (YY1) were directly bound to the LINC01578 promoter.^213^ Meanwhile, upregulated LINC01578 interacted with and recruited EZH2 to NFKBIB promoter and further repressed NFKBIB expression, thereby activating NF-κB signaling. LINC01578 and NF-κB/YY1 formed a positive feedback loop, which promoted CRC metastasis. LncRNA HOTAIR suppressed HNF4α via recruiting SNAIL to promote the migration, invasion and EMT of CRC.^214^ Intriguingly, a novel lncRNA, ENSG00000274093.1 binds to histone deacetylase 2 (HDAC2) and may act as a modular scaffold for the HDAC1/HDAC2 and EZH2 complexes, thereby altering EMT in CRC.^215^ LncRNA TPT1-AS1 induces angiogenesis and metastasis in CRC via theTPT1-AS1/NF90/VEGFA axis.^216^ Additional research have shown that the expression level of LncRNA clmat3 and SNHG15 in CRC with liver metastasis was significantly higher than in those CRC without liver metastasis.^217,218^ Conversely, some lncRNAs play a role of tumor inhibition, for example, low expressed lncRNA SATB2-AS1 inhibited cell metastasis and regulated the immune response of CRC by *cis*-activating SATB2.^219^ Moreover, lncRNA MIR22HG, as a tumor suppressor in CRC, competitively interacted with SMAD2 and modulated the activity of TGFβ pathway, thereby inhibited cell survival and tumor metastasis in vitro and in vivo.^220^

LncRNA UICLM acted as a ceRNA for miR-215 to regulate ZEB2 expression, then induced CRC liver metastasis, which may offer a novel prognostic marker and therapeutic target for this disease.^221^ LncRNA GAPLINC, HOTAIR, RP11-757G1.5, LINC00460, MIR4435-2HG, PlncRNA-1, DANCR and TP73-AS1 were proven to be positively associated with the proliferation and liver metastasis of CRC through the GAPLINC/miR-34a/c-MET axis, HOTAIR/miR-214/ST6GAL1 axis, RP11-757G1.5/miR-139-5p/YAP1 axis, LINC00460/miR-613/sphingosine kinase 1 (SphK1) axis and MIR4435-2HG/miR-206/YAP1 axis, PlncRNA-1/miR-204/Wnt/β-catenin regulatory network, DANCR/miR-577/HSP27 signaling axis and TP73-AS1/miR-194/TGFαsignaling axis, respectively.^222–229^ Furthermore, lncRNA ZEB1-AS1 acted as ceRNA to upregulate p21-activated kinases 2 (PAK2) by sponging miR-455-3p, thus facilitating colon adenocarcinoma cell growth and metastasis.^230^ Zhou et al.^231^ reported that SRY-box transcription factor 9 (SOX9) activated the transcription of lncRNA FARSA-AS1. FARSA-AS1 upregulated SOX9 and FARSA via binding to miR-18b-5p directly. Overall, SOX9-FARSA-AS1-SOX9/FARSA loop participated in cell growth, stemness, and metastasis in CRC. In addition, lncRNA HSD17B11-1:1 acted as a sponge for miR-338-3p to upregulate the expression of MACC1 to promote CRC cell proliferation, mobility and invasion in vitro and in vivo.^232^ B3GALT5‐AS1, is an antisense lncRNA, located in Chr21q22.2, which was first reported in Wang et al. study.^233^ They found B3GALT5‐AS1 was decreased in CRC contrast to normal colonic epithelium. B3GALT5-AS1 directly bound to the promoter of miR-203, repressed miR-203 expression, upregulated miR-203 targets ZEB2 and SNAI2, and induced EMT, suggesting that downregulated B3GALT5‐AS1 is a biomarker of CRC metastasis and, more importantly, activating B3GALT5-AS1/miR-203/EMT axis may be a potential therapeutic strategy for CRLM.^233^ Recently, B3GALT5‐AS1 has been reported to be related to TNM stage and histological differentiation.^234^ There are also other lncRNA that regulated CRLM through ceRNA mode, for example MALAT1, SNHG14, SNHG7, H19, and LINC00485. Taken together, these lncRNA could be a therapeutic target and promising biomarkers for prognosis prediction of CRLM.

#### CircRNAs in CRLM

NSUN2 is a circRNA recently identified upregulated in CRLM and N6-methyladenosine modification of circRNA among which circRNA circPPP1R12A activated the Hippo-YAP signaling pathway to promote the growth and metastasis of CRC^235^. NSUN2 was more likely to enter the cytoplasm, then in the cytoplasm increased the stability of HMGA2 mRNA to induce CRC metastasis progression.^236^ Equally noteworthy, circRNA circ_0124554 inhibited the ubiquitination of AKT to promote the early metastasis particularly for the lymph node-negative CRC patients with synchronous liver metastasis.^237^

CircRNAs serve as a sponge to protect multiple oncogenes from being attacked by miRNAs and among these, global exaltation of circ_0115744 circRNAs circAPLP2, hsa_circ_000984, hsa_circ_0071589 and hsa_circRNA_102209 acted as ceRNA to enhance tumor metastasis via circ_0115744/miR-144/EZH2 axis, circAPLP2/miR-101-3p/Notch1 axis, hsa_circ_000984/miR-106b/CDK6 axis, hsa_circ_0071589/miR-600/EZH2 axis and hsa_circRNA_102209/miR-761/RIN1 axis, respectively.^238–240^ Similarly, circRNA NSD2 promoted metastasis of CRC by sponging miR-199b-5p, which could increase the expression of DDR1 and activate JAG1 signaling.^241^ More interestingly, one circRNA contains various binding sites for different miRNAs reveals the complex role of circRNA in cancer malignancy. CircRNA CCDC66 functions as miRNA sponge to reduce the destruction of MYC mRNA by miRNA-33b and miR-93.^242^ In addition, Zhi et al.^243^ also identified a novel and conserved circRNA hsa_circ_102049 as a promoter of CRC metastasis. Mechanistically, hsa_circ_102049 acted as a sponge of the tumor suppressor miR‐761 and miR‐192‐3p and modified the subcellular localization of the RNA binding protein DGCR8, then regulated the levels of the FRAS1, which synergistically enhanced the adhesion, migration, and invasion abilities of CRC cells. Interestingly, hsa_circ_0001178 sponging miR-382, miR-587 and miR-616 to upregulate ZEB1, which in turn increase hsa_circ_0001178 expression via physically binding to hsa_circ_0001178 promoter region, thereby based on this positive feedback ceRNA axis, consequently facilitated the invasion and metastasis of CRC.^244^

circRNAs also play an inhibition role of CRLM, for example, circRNA Hsa_circ_0009361 acted as an inhibitor for miR-582 and suppressed the CRC cell metastasis.^245^ Similarly, circRNA-FNDC3B negatively and directly regulated miR‐937‐5p to promote the expression level of the tumor‐suppressor TIMP3, thereby blocking the metastasis, invasion and angiogenesis of CRC.^246^ In addition, circITGA7 inhibited the Ras signaling pathway and promoted the transcription of ITGA7, thereby suppressed the proliferation and metastasis of CRC cells.^247^

In the latest studies, circRNA_0001178, circRNA_0000826 and circRNA hsa_circ_0000826 induced by the hypoxia in CRC were dramatically upregulated in CRLM tissues, which can be a potential biomarker of CRC liver metastasis.^248,249^ Validation studies of specific molecular mechanisms are needed to identify optimal miRNAs and marker panels that can be used in clinical care.

#### MiRNAs in CRLM

Recent studies have shown that miRNAs were involved in the context of EMT in CRC. It has been reported that miR-200 family (miR-200a, miR-200b, miR-200c, miR-141, and miR-429) are recognized as regulators of the epithelial phenotype through repression of ZEB1 and ZEB2 mRNA translation^250–252^ The miR-200c levels in primary CRC without liver metastasis is lower than the metastasis with primary tumor tissues, which highlight a crucial role for miR-200c in CRC metastasis.^251^ The overexpression of miR-429 could play an oncogenic role in the cellular processes of CRC by targeting SOX2.^253^

Additionally, miR-181a and miR-30b were highly expressed in CRLM by promoting EMT through inhibiting Wnt inhibitory factor-1 (WIF-1) and inhibiting the SIX1 gene, respectively.^254,255^ Moreover, miR-30a is an important regulator of transmembrane-4-L-six-family protein (TM4SF1), VEGF, and E-cadherin for CRC cell motility and EMT.^256^ Manganese superoxide dismutase (MnSOD) was required for downregulation of epithelial markers and upregulation of mesenchymal markers in CRC cells, indicating that it promoted the EMT, which was reduced by overexpression of miR-212.^257^ Methylated miR‐34c‐5p significantly suppressed the metastasis of CRC cells via directly modulating the SATB2.^258^ Recently, the cytokine IL-6 was found to activate the oncogenic STAT3 transcription factor, which directly repressed the MIR34A gene, while miR-34a directly regulated IL-6R. As a result, the IL-6R/STAT3/miR-34a loop promoted CRC invasion and metastasis.^56^ MiR-186-5p affected metastasis and EMT process of CRC cell by inhibition of ZEB1, while miR-17-5p regulated EMT by targeting vimentin.^259,260^

Furthermore miR-34a/SNAIL loop and miR-200/ZEB1/2 loops, miR-15a/16-1/AP4 feedback loop is also found in primary CRC. The tumor-suppressive miR-15a and miR-16-1, which targeted AP4 3′-UTR, and inhibited CRC cell migration and invasion.^261,262^ Overexpression of miRNA let-7 promoted EMT via targeting HMGA2^263^, while Lin28 could inhibit let-7 in conjunction with OCT4, SOX2, and KLF4.^264^ MiR-10a suppressed CRC metastasis by regulating the EMT via targeting matrix metalloproteinase 14 and actin gamma 1 (ACTG1).^265^ Unlike miR-10a, upregulated miR-10b in metastatic CRC tissues and cell lines inhibited E-cadherin expression and enhanced cyclin D1, which were partly abrogated after targeting KLF4.^266^

The expression of miR-21 was higher in tumor tissue than in adjacent normal tissue of 156 CRC patients by TaqMan MicroRNA assays.^267^ It is reported that transglutaminase 2 (TG2) expression was observed in CRC primary tumors but lost in liver metastases and TG2 inhibited by miR-19 could affect the invasive ability of CRC cells.^268^ Moreover, Zhang et al.^147^ demonstrated that the overexpression of PRL-3 in CRC cells induced the expression of miR-21, miR-17 and miR-19a by activating signal transducer and STAT3. A positive correlation was observed between PRL-3 and these miRNAs in matched primary colon cancer tissues and metastatic lesions.^147^ MiR-21 could significantly reduce Pdcd4-protein amounts and increased invasion.^269^ Additionally, Feiersinger et al.^270^ have identified that the expression of miR -21 was significantly lower in liver metastases as compared to the primary CRC. The overexpression of miR-200 and miR-141 inhibited apoptosis and induced migration.^271^ These indicated that miR-21 might be involved in the initiation and miR-200 and miR-141 exacerbated liver metastasis of CRC.

Interestingly, highly expressed miRNA-20a-5p and miR-298 were positively correlated with CRLM by suppressing drosophila mothers against decapentaplegic protein4 (smad4) expression and targeting PTEN, respectively.^272,273^ Inhibition of miR-15b significantly decreased colony formation ability, invasion, and migration of HCT116 cells in vitro and liver metastasis of HCT116 tumors in vivo via increasing metastasis suppressor-1 (MTSS1) and Klotho protein expression.^274,275^ Upregulation of miR-885-5p had strong tumor-promoting effects through by targeting cytoplasmic polyadenylation element binding protein 2 (cpeb2), von Willebrand factor and insulin-like growth factor binding protein 5, and its potential role in promoting cell migration, invasion and liver metastasis.^276,277^ The expression of miR-224 increased consistently with tumor burden and the steady state of microsatellites, and can enhance CRC metastasis in vitro and in vivo.^278^

For long, it was known that the patients with CRC have demonstrated a significant evaluated expression of serum miR-497.^279^ In the mouse model, Qiu et al.^280^ found that a combination of miR-497 and bufalin had a synergistic effect on the inhibition of CRC metastasis. They also found that miR-497 targeted the expression of vascular endothelial growth factor-A (VEGF-A).^281^ Notably, miR-497 exerted its oncogenic function by targeting fos-related antigen-1 (Fra-1).^282^ Recently, AGAP2-AS1 regulated fibroblast growth factor receptor 1 (FGFR1) expression by sponging miR-497 in the migration and invasion of CRC cells.^283^

However, liver metastasis cells of CRC also downregulate the expression of miR-133a, miR-17-5p, miR-99b-5p, miR-214, miR-26, miR-30e-5p and miR-328-3p that suppress cell migration and invasion. MiR-133a, as a tumor suppressor, inhibited cell proliferation, invasion, and migration by targeting oncogenic EIF4A1.^284^ MiR-99b-5p was differently expressed in primary CRC and liver metastasis and functioned as a tumor-suppressive miRNA to affect cell migration by targeting mTOR in metastatic CRC.^285^ In an orthotopic mouse model of CRC, Ding et al.^286^ found that APOBEC3G enhanced CRC cell migration and invasion via inhibition of miR-29-mediated suppression of MMP2. Downregulation of miR-214 promoted proliferation, migration, and invasion in CRC cell lines via increasing level of FGFR1, which can lead to the occurrence of liver metastasis.^287,288^ Li et al.^289^ reported that tumor-suppressive miR-26a and miR-26b inhibited the target gene FUT4 expression, resulting in migratory behavior of CRC. Laudato et al.^290^ found that miR-30e-5p was a novel effector of P53-induced suppression of migration, invasion, and metastasis by directly targeting both integrin alpha‐6 (ITGA6) and integrin beta‐1 (ITGB1). In addition, miR-30b-5p functioned as a metastasis suppressor by targeting Rap1b, a Ras family small GTPase that regulates cell adhesion and mobility.^291^

A recent study has shown that low miR-196b-5p expression is significantly associated with metastases and poor outcomes, further proving that miR-196b-5p inhibition led to significantly increased CRC cell migration/invasion and metastases formation in mice via the interaction with HOXB7 and GALNT5.^292^ A contemporary study by Luo et al. has demonstrated that miR-432-5p functions as a tumor suppressor to inhibit cell migration and invasion by negatively regulating CXCL5 expression.^293^ Interestingly, miR-328-3p may inhibit proliferation and metastasis of CRC cells via suppressing Girdin expression and associated PI3K/Akt signaling pathway^294^ MiR-365a-3p to inhibit the metastasis of CRC cells by negatively regulating ADAM10 and inactivating the JAK/STAT signaling pathway.^295^ Moreover, miR-487b directly targeted LRP6, a receptor for WNT/β-catenin signaling to inhibit liver metastasis.^296^ MiR-143-3p, miR-132 and miR-146a significantly abolished the development of liver metastases by directly targeting ITGA6/ASAP3, anoctamin 1 (ANO1) and c-Met, respectively.^297–299^ In addition to control the intravasation, miRNAs also targeted genes; for example, miR-21 regulated Pdcd4 which induced intravasation and metastasis.^269^ A positive significant correlation between expression of miR-126 and epidermal growth factor–like domain 7 (EGFL7) were seen in liver metastases, which supported miR-126 may act as a regulator in angiogenesis and intravasation process.^300^ Further experiments are needed to explore more detailed forms of connection between miR-126 and EGFL7. Low expression of miR-26b was significantly associated with the invasiveness and metastasis of CRC cells. Hansen et al.^301^ reported that miR-31-mediated repression of E-selectin impaired the metastatic potential of CRC cells.

One study showed that miR-103/107 potentiated the colonization of CRC cells at a metastatic site by targeting the known metastasis suppressors death-associated protein kinase (DAPK) and KLF4 in CRC cells.^302^ Similarly, in mice models of CRC, the overexpression of miR-103/107 enhanced local invasion and liver metastasis effects. Another report revealed that miR-483 and miR-551a inhibited liver colonization and metastasis.^303^ Metastatic foci in new organs promote angiogenesis due to increased demand for oxygen and nutrients. Therefore, angiogenesis has become a necessary condition for the survival of metastatic foci.

### Cancer stem cells (CSCs) in the progression to CRLM

CSCs as seed cells for tumorigenesis can initiate and sustain the growth of tumors. CSCs can be identified by specific markers, including CD133, LGR5,CD44, ALDH1, CD24, CD166, CD29, CD26, and CD51.^304,305^ CRC-CSCs can increase the ability of distant metastasis and colonization.^306,307^ It is thus possible that targeting CSCs may have widespread clinical implications.^308^ Among the epithelial cells that form the surface of the intestine, crypt-based columnar cells (Lgr5+ cells) expressing the receptor protein Lgr5 function as stem cells during the maintenance of intestinal homeostasis, and they are also the starting cells for CRC.^309^ The intestinal epithelium has significant self-renewal ability, and the rapidly proliferating Lgr5+ cells are usually responsible for the daily production of all types of intestinal epithelial cells.^309^ Lgr5− cells can drive wound healing by first transdifferentiating into a stem-like Lgr5+ state.^310^ The selective elimination of LGR5+ cells leads to temporary tumor regression, and other cells exhibit compensatory proliferation, then the reappearance of LGR5+CSCs drives tumor regeneration.^311^ It has been demonstrated that selective Lgr5+ cell ablation restricts primary CRC growth, but does not result in tumor regression, because proliferative Lgr5− cells could continuously attempt to replenish the Lgr5+ CSCs pool, leading to rapid re-initiation of tumor growth upon treatment cessation.^312^ Moreover, Fumagalli et al. provided direct evidence most disseminated CRC cells in circulation were Lgr5- and formed liver metastases in which Lgr5+ CSCs appeared.^313^ Cell state plasticity is very important to promote CRC metastasis. The signaling pathways responsible for the reproduction of Lgr5+ cells may yield exciting new strategies for treating CRLM. Wang et al.^314^ reported prostaglandin E2 induced CSCs and enhanced liver metastasis by activating NF-κB via EP4-MAPK and EP4-PI3K-Akt pathways.

DNA methylation is considered to be a potential epigenetic mechanism to maintain CSCs, and the loss of DNMT can reduce the occurrence of tumors by limiting the CSC pool.^315^ Li et al.^316^ found that the DNMT inhibitor 5-Aza-2'-deoxycytidine (5-AzaDC) significantly reduced the abundance of colorectal CSC in vitro and inhibited the growth of liver metastatic tumors in vivo. They also found that 5-AzaDC inhibits the expression of active β-catenin and downregulates the Wnt signaling pathway. A new study points out the mechanism exerted by methylation on endothelin system genes expression can be compromised in CRLM.^317^ Notably, CSCs are critical for the formation and maintenance of liver metastasis. Together, our data highlights distinct CSC dependencies for primary versus metastatic tumor growth and suggest that targeting CSCs may be one direction of treatment for CRLM.

### Role of circulating tumor cells (CTCs) in CRLM

Patient-derived CTCs has been shown to bear all the functional attributes of CSCs.^318^ The markers expressed by CTCs are similar to the cancer niche, which are conducive to liver metastasis.^319,320^ CD133+CD44 + CD54 + cellular subpopulation of CTCs has a prognostic value in CRLM, especially in the survival of CRLM who did not receive surgical treatment for metastasis.^321^ Patient-derived CTCs lines are tumorigenic in subcutaneous xenografts and are also able to colonize the liver after intrasplenic injection.^318^ Drug test by in vitro culture of CTCs may facilitate access to personalized medicine. TAMs regulated JAK2/STAT3 signaling pathway by secreting IL6, thereby inhibiting miR-506-3p expression and promoting FoxQ1 expression. CTC cells then produced CCL2 to recruit more TAMs. TAMs and CTC both interacted to promote the occurrence of metastasis.^322^ These findings suggest targeting strategies against CTC clusters may be effective in the treatment of liver metastases.

### Metabolic factors in CRLM

Tumors are also a metabolic disease, and metabolic changes are closely related to every process of tumor metastasis. The process of transfer requires a large amount of energy supply, and some enzymes and molecules that affect energy metabolism also act as gas stations in the process of transfer. In the extracellular space, creatine kinase brain (CKB) used ATP-catalyzed phosphorylation of the metabolite creatine to form phosphocreatine, which can produce large amounts of ATP into CRC cells as an energy reserve to maintain the energy requirements of CRC cells during anoxia during metastasis.^323^ CKB promoted the development of liver metastasis, and targeted inhibition of its activity was also the direction of future treatment of CRLM. In addition to phosphocreatine generating energy for cancer cells, the fatty acid oxidation (FAO) pathway is also an important energy source within cancer cells. In detached CRC cells, under the action of carnitine palmitoyl transferase 1, FAO was greatly activated, thus increasing the ability of cells to metastasize.^324^ CRC cells implanted in the liver promoted the metabolism of fructose by upregulating enzyme aldolase B (ALDOB), which provided energy for cell growth in liver metastases.^325^ This particular metabolic pathway change was found only in liver metastases and not in other sites of metastasis or primary tumors. All in all, the metabolic pathways of tumors adapt to the environment during metastasis, and there are still many problems to be studied.

## Diagnosis

CRLM should be identified in patients with a confirmed diagnosis of CRC. Among them, ~10–15% of patients have synchronous liver metastases—the liver metastasis is diagnosed at the same time with CRC.^10^ The main content of our diagnostic section is centered on synchronous liver metastases, and the assessing approaches of metachronous liver metastases are similar to the former and executed during the follow-up period.

Biomarker testing should be performed routinely for diagnosis of the synchronous liver metastasis. Serum cancer embryo antigen (CEA) could be the routine choice for screening, and carbohydrate antigen 19-9 (CA19-9) might be a supplementary indicator without an evaluated CEA value.^326^ Detection of CEA and CA19-9 should also be executed at intervals after resection. RAS is also a biomarker predicting the efficacy of anti-epidermal growth factor receptor (EGFR) therapy, which determines the choice of treatment strategy. Thus, RAS testing should be executed in all patients with CRC, and include NRAS exons 2, 3, 4 and KRAS exons 2, 3, 4 at least.^327^ BRAF testing also could help to select the therapy option and predict the prognosis. In addition to serum tumor marker examination and pathological staging evaluation, imaging examinations such as liver ultrasound and abdominal contrast-enhanced CT should be routinely performed to screen and diagnose liver metastases.^328^

Magnetic resonance imaging (MRI) with liver specific contrast should be used prior to surgery when the liver metastases are resectable. Moreover, MMR/microsatellite instability (MSI) testing, UGT1A1 detection of UGT1A1 protein, human epidermal growth factor receptor 2 detection are recommended to provide the basis for clinical decisions on post line treatment in advanced patients. The PET-CT examination is not used as a regular recommendation and may be applied as appropriate when the condition is required. Needle biopsies of liver metastases are rarely required.^329^

After radical CRC, patients should follow up regularly to understand whether the occurrence of metachronous liver metastases. During CRC surgery, routine exploration of the liver must be performed to further exclude the possibility of liver metastases. Suspicious liver nodules found during the operation can also be biopsied.^329^ Tumor markers such as serum CEA, CA19-9 and, liver ultrasound and chest/abdominal/pelvic enhancement CT scan should be routinely examined to conduct screenings and diagnose liver metastases. Patients with high suspicion in ultrasound or CT imaging should not be diagnosed with liver MRI, and recommended consistency of imaging methods during follow-up. PET-CT scan is not normally recommended. Electronic colonoscopy should be performed within 1 year after surgery.^330^

## Treatment

### Surgery

Surgery has become the standard of care for patients with CRLM. When CRLM may be limited to a few liver metastatic foci, surgical resection is the first choice. Patients with liver metastases that cannot be resected initially should also receive surgical treatment when they are transformed into resectable lesions after treatment.^331^ For these patients who are suitable for complete surgical resection, they are often accompanied by lymph node infiltration and dissemination of occult micro metastases. Therefore, it is necessary to cooperate with adjuvant radiotherapy and chemotherapy and targeted therapy.

### Radiation therapy

In patients with CRLM, radiation therapy of liver metastasis remains controversial, due to the tolerated dose of whole liver radiation being much lower than the lethal dose required by tumor cells. The use of image guidance technology can make radiotherapy more precise. For patients with liver metastases from CRC with normal liver function, conventional radiotherapy techniques can be used to treat liver metastases.^332,333^

### Ablation therapy

In cases where liver metastases cannot be surgically removed, appropriate ablation treatments, such as radiofrequency ablation, microwave ablation, and cryotherapy, should be selected on the basis of systemic chemotherapy based on their location, treatment goals, treatment-related complications, and the patient’s own conditions to strengthen control of local lesions.^334^ However, care should be taken to avoid extrahepatic heat damage and incomplete ablation when performing ablation treatment.^331^

### Systemic therapy

Patients with CRLM usually receive chemotherapy after surgical resection. It recommends FOLFOX (5‐fluorouracil [5‐FU] + oxaliplatin + leucovorin [LV]), FOLFIRI (5‐FU + irinotecan+ LV), XELOX (capecitabine + oxaliplatin), infusional 5‐FU/LV or capecitabine, or FOLFOXIRI (5‐FU + oxaliplatin + irinotecan + LV) for patients with mCRC who are suitable for intensive therapy according to the current National Comprehensive Cancer Network guidelines.^335^ Research data in recent decades has shown that patients with metastatic CRC receive systematic chemotherapy, and their OS duration is extended to nearly 20 months.^335,336^ Currently, patients with initially unresectable CRLM has received hepatic arterial infusion chemotherapy in combination with systemic chemotherapy, which is not widely used.^337^

### Anti-angiogenesis therapy

Tumor increases energy supply through angiogenesis during metastasis, and anti-angiogenesis therapy has become an important therapeutic strategy for CRC. It is of great significance to explore the angiogenesis mechanism of CRLM. The targeted agents for anti-angiogenesis therapy under clinical trials in CRLM are summarized in Table 6. Bevacizumab and Cetuximab are molecularly targeted drugs that have been developed specifically for endothelial growth factor receptor (EGFR). Clinical trials have proven that anti-EGFR monoclonal antibodies panitumumab and cetuximab can effectively inhibit metastatic CRC.^338^ Saltz et al.^339^ evaluated the efficacy of adding bevacizumab to XELOX or FOLFOX-4 in 1401 CRLM patients. The median duration of PFS was 9.4 months in the bevacizumab group and 8.0 months in the placebo group (*P* = 0.0023). Moreover, cetuximab added to FOLFOX-4 compared with FOLFOX-4 alone, cetuximab was associated with a clinically significant increase in overall response (61% vs. 37%; *P* compared with FOLFOX-4 alone, especially In KRAS wild-type tumors, it can reach a level of 0.011), which reduces the risk of disease progression (hazard ratio = 0.57; *P* = 0.0163).^340^ Patients with RAS wt mCRC do not respond to anti-EGFR antibodies, probably due to ineffective inhibition of oncogenic RAS signaling.^341^ A recent randomized study conducted by Qin et al.^342^ confirmed cetuximab in combination with FOLFOX as an effective standard-of-care first-line treatment regimen for patients with RAS wt mCRC. However, the NORDIC-VII multicenter phase III trial suggested that cetuximab did not add significant benefit to the fluorouracil, leucovorin, and oxaliplatin (Nordic FLOX) regimen in first-line treatment of mCRC.^343^

**Table 6:** Targeted agents for anti-angiogenesis therapy under clinical trials

NCT numbers	Phase	Therapeutic agents	Condition	Result
NCT01001377	Phase 3 (*N* = 1010)	Panitumumab vs Cetuximab	mCRC (KRAS-WT)	Median OS 10.4 m VS 10 m
NCT01228734	Phase 3 (*N* = 393)	Cetuximab + FOLFOX-4 vs FOLFOX-4	mCRC (RAS-WT) First line	PFS 9.2 m vs 7.4 m Median OS 20.7 m vs 17.8 m
NCT00265850	Phase 3 (2334)	Cetuximab + mFOLFOX6 vs bevacizumab + mFOLFOX6	mCRC (KRAS-WT)	PFS 10.5 m vs 10.5 m Median OS 30 m vs 29 m
NCT00006479	Phase 3 (364)	Perioperative chemotherapy with FOLFOX4 vs surgery alone	CRLM	Median OS 61.3 m vs 54.3 m 5-year OS 51.2% vs 47.8%
NCT22944367	Phase 3 (257)	Cetuximab + chemotherapy vs chemotherapy alone	CRLM	Median PFS 15.5 m vs 22.2 m Median OS 55.4 m vs 81 m

*DFS* disease-free survival, *OS* overall survival, *FOLFOX4* folinic acid + fluorouracil + oxaliplatin, *mFOLFOX6* leucovorin + fluorouracil + oxaliplatin

It was confirmed by clinical trials that dual chemotherapy (fluoropyrimidine plus oxaliplatin or irinotecan) with targeted drugs that add anti-EGFR antibodies can further increase the response rate to about 60%.^339,344,345^ Bevacizumab plus XELOX (CapeOX) chemotherapy has no negative impact on intrahepatic immune cells in resectable CRLM patients.^346^ Although the survival rate is improved by FOLFOX treatment, chemotherapy still has many disadvantages, such as the destruction of the human immune system, loss of appetite, weight loss, hair loss and so on after chemotherapy. In a recent study, nano codelivery of oxaliplatin and folinic acid (Nano-Folox/5-Fu) with 5-fluorouracil significantly promoted the blood circulation and tumor accumulation of drugs in the orthotopic CRC mouse model.^347^ Nano-Folox/5-Fu with 5-fluorouracil can not only provide anti-cancer cell toxicity, but also induce immunogenic cell death. In addition, the combination of anti-PDL1 monoclonal antibody with Nano-Folox/5-FU significantly reduced liver metastasis in mice. These results suggest that the combination strategy based on Nano Folox has some therapeutic significance in CRLM. Moreover, gene set enrichment analysis discovered that the mTOR pathway was activated in patients undergoing oxaliplatin based therapy, which suggested it could be used in combination with cytotoxic chemotherapy and targeted drugs, and may have good therapeutic effects.^348^ For patients with initially resectable CRLM, chemotherapy can improve progression-free survival (PFS) but cannot improve OS^349^, and further addition of EGFR-targeted antibodies is not beneficial.^350^ However, in patients with unresectable CRLM, the use of bevacizumab may further increase the proportion of patients eligible for surgical resection.^351^ For patients whose CRLM is initially unsatisfactory or unresectable, targeted anti-angiogenesis drugs plus chemotherapy can also produce a higher remission rate and improve resectable.^352^

TSU68[(Z)-5-[(1,2-dihydro-2-oxo-3H-indol-3-ylidene)methyl]-2,4-dimethyl-1H-pyrrole-3-propanoic acid; SU6668] is a potent anti-angiogenic agent. In some preclinical models, TSU68 has previously been shown to have a powerful effect in preventing liver metastasis of CRC.^353,354^ Inhibiting CXCL1 expression in the premetastatic liver was the detailed mechanisms by which TSU68 suppresses tumor metastases.^71^ Anti-angiogenic agents may modulate the PMN in target organs.

### Immunotherapy

In addition to anti-angiogenesis therapy, immunotherapy becomes an attractive therapeutic option as more progress has been made in the exploration of immune checkpoint in several cancer types, mainly including DNA mismatch repair defects(dMMR)/high microsatellite instability (MSI-H) CRC and high tumor infiltrating lymphocyte (TIL) tumors. By contrast, immune checkpoint monotherapy has not demonstrated significant clinical success in patients with strong mismatch repair capacity (pMMR)/low microsatellite instability (MSI-L) CRC. Immune checkpoint drugs target the inhibitory receptors present on T cells, such as PD1, LAG3 and cytotoxic T-lymphocyte associated antigen 4 (CTLA-4).^355^ These cells mainly exist at the tumor-stroma interface, while pMMR/MSI-L CRC shows a conventional morphology without obvious infiltrating lymphocyte TIL.^356^

PD1, a novel member of the immunoglobulin gene superfamily, has two functional ligands: PDL1 and programmed cell death ligand 2(PDL2).^357,358^ PD1/PDL1 signaling pathway can inhibit the activation of T effector cell while inhibiting the production of interferon gamma and the release of interleukin (IL)-2 and other inflammatory cytokines, thereby enabling cancer cells to escape from the host’s antitumor immunosurveillance.^359,360^ Masugi et al.^361^ reported that tumor PD1 expression was inversely associated with MSI-H in CRC. However, Wyss et al.^362^ reported stromal PDL1 might function as a prognostic marker in CRC patients independent of microsatellite-stable (MSS) or with MSI-L. Masugi et al.^361^ found that stromal PDL1 expression was only present in 5% CRC patients, but the studies by Wyss et al.^362^ and Taube et al.^363^ displayed a stromal PDL1 expression rate of 60.9% and 50%, respectively. However, epithelial PDL1 staining is less common in CRC than in other solid tumors, such as melanoma.^364^ While there is an inequality of PDL1 expression between primary tumors and metastases in melanoma patients,^365^ PDL1 expression in primary colon tumors largely corresponds to matched liver metastases.^362^ Another study revealed the gene networks of EMT, angiogenesis, immune-suppression and T cell exhaustion are the key events closely associated with CRC metastasis and intrinsic anti-PD1 resistance.^366^ If anti-PDL1 treatment for metastatic colon cancer is needed, it may not be necessary to analyze PDL1 in liver metastases. The PDL1 and CRLM research is highly debated, therefore PD1/PDL1 signaling pathway may be closely related to CRLM and further exploration is needed.

A small number of patients with MSI-H CRC were sensitive to immune checkpoint blockade with antibodies to PD1/PDL1.^367,368^ The incidence of dMMR in CRLM is also low.^369^ Recently, a study has found that tumor mutational burden (TMB) is a predictor of response to immune checkpoint inhibitors in the MSI-H population. Patients with high TMB may respond particularly well to immune checkpoint inhibitors, and these patients can be further selected to receive anti-PD1 monotherapy rather than combination therapy (such as nivolumab) as first-line treatment.^370^ Nevertheless, in recent years, there have been new advances in immunotherapy for CRLM. Next, we summarized the latest progress in the clinical development of immune checkpoint blockade therapy for patients with CRLM.

CTLA-4 is predominantly expressed on T cells and inhibits the activation and response of T cells.^371^ In a MSS highly aggressive orthotopic mouse model of CRC, dual inhibition of CTLA-4 and PDL1 resulted in the arrest of tumor growth and entirely blocked liver metastasis. Nevertheless, inhibition of CTLA-4 or PDL1 alone only moderately reduced metastatic diffusion of CRC cells.^372^ Furthermore, pMMR CRLM differs immunologically from primary CRC in terms of immune infiltration.^373^ Zhou et al.^374^ focus on TIL regulated by the inhibitory receptor-ligand pathway regulates MMR-proficient CRLM. They found that blocking LAG3 enhances tumor-infiltrating T-cell responses of MMR-proficient CRLM, and may thus be a new target of immunotherapy for CRLM.

Unlike primary CRC, tumor-infiltrating regulatory T cells (Ti-Treg) in liver metastases effectively inhibit tumor-specific T cell responses by expressing high levels of Treg-related molecule glucocorticoid-induced tumor necrosis factor receptor (GITR) and CTLA-4.^375,376^ It has demonstrated that treating CRLM by inhibiting the inhibitory receptor CTLA4 with antibodies and activating the stimulant receptor GITR with natural ligand of GITR can reduce Ti-Treg-mediated inhibition, thereby restoring effector T cell proliferation and cytokine formation.^377^ More importantly, compared with any single treatment, the low-dose combination therapy of the two molecules showed a stronger ability to restore T cell function. Based on gene ontology and Kyoto Encyclopedia of Genes and Genomes pathway analyses, Liu et al.^378^ identified some immune genes *CCL20, CCL24* and *CD70* that were associated with CRLM, but needs further experimental validation.

Furthermore, the relationship of gut microbiome and immune system is nowadays rapidly changing and expanding. Host factors, including age, obesity and gut microbiome greatly affect the effectiveness of immunotherapy.^379,380^ Meanwhile, gut microbiome is closely linked to the development and progression of CRC. Lipopolysaccharide (LPS), an important product of intestinal Gram-negative microbiota, was also found to participate in the whole process of CRLM. Systemic inflammation caused by elevated LPS blood levels in the intestinal cavity and portal vein in CRC patients could increase the liver recruitment of cancer cells.^381^ LPS enhanced CRLM by stimulating Toll-like receptor 4 signaling and increasing β1 integrin-mediated cell adhesion.^382^ LPS was observed to promote the migration capacity of CRC cells by activating the SDF-1α/CXCR4 axis and EMT.^381^ These results revealed trapping LPS may prevent CRLM. Recently, Song et al.^194^ reported nanotechnology-based trapping LPS in the orthotopic CRC tumor could promote T-cell infiltration into tumors and promote enhanced immunotherapy.

Antartina, an antitumor agent isolated from Deschampsia antarctica Desv, potently inhibited tumor growth and liver metastases in an immunocompetent colorectal carcinoma mice model.^383^ Antartina induced a potent specific cytotoxic T-cell response against CRC and a long-lasting antitumor immunity. Antartina can induce antitumor immunity against CRLM. Based on the results of one phase III multicenter trial, Keytruda is superior to chemotherapy in the first-line treatment of MSI-H or dMMR CRC, resulting in longer PFS and fewer treatment-related adverse events.^384^

### Other potential therapies

Current chemotherapy for CRLM is still not desirable owing to off-target effect. Therefore, it is necessary to develop new methods to improve target effect or even replace the existing CRLM chemotherapy. Zhao et al.^385^ found α5β1 integrin receptor expression on metastatic cells was higher than that of the original cells from orthotopic tumors. They synthesized RPM-conjugated, α5β1-targeted micelles (RPM-CSOSA), which could enhance the cellular internalization and distribution in metastatic lesions by binding to α5β1 integrin. Doxorubicin (DOX) was a potent cytotoxic drug and curcumin (CUR) exerts its anti-cancer effects as chemosensitizer.^386^ Zhao et al.^385^ also found that the therapy of RPM-CSOSA/DOX and RPM-CSOSA/CUR significantly inhibited the progression of liver metastasis in vivo and in vitro.

Endothelin, a coreceptor of transforming growth factor-β, is preferentially expressed in solid tumor angiogenic endothelial cells.^387^ Serum endoglin is a useful marker for monitoring early signs of CRC metastasis.^388^ As an endoglin neutralizing antibody, TRC105 binds to human endoglin with high affinity and is associated with antibody-dependent cell-mediated cytotoxicity.^389^ Targeting endoglin with TRC105 strongly inhibits metastatic spread of breast cancer in vivo.^390^ Similarly, Targeting endoglin using TRC105 decreased metastatic spread of CRC cells to the mouse liver.^391^ In a phase I first-in-human study, TRC105 showed clinical efficacy on preexisting metastases in 2 patients.^392^ Collectively, these results demonstrated that in addition to endothelial cells, targeting endoglin to CAFs may be a potent method to prevent metastases formation, and emphasizes the potential of TRC105 for the treatment of metastatic tumors, not just a classic anti-vascular generate drugs.

The cytoskeleton of the cell and ECM determine the stiffness of the tissue.^393,394^ Highly activated metastasis-associated fibroblasts cause the ECM to stiffen, which in turn increase tissue stiffness. Metastasis stiffness influences the effect of anti-angiogenic therapy on intratumoral blood vessel reduction. Shen et al.^395^ reported that anti-hypertensive drugs targeting the renin-angiotensin system (anti-RAS) in combination with bevacizumab could significantly improve anti-angiogenic efficacy in CRLM. The mechanism is that anti-RAS inhibit fibroblast contraction and ECM deposition, thereby reducing the hardening of liver metastases and enhancing the anti-angiogenic effect of bevacizumab. This research highlights a new mechanism of action for anti-RAS drugs in cancer that could lead to new therapeutics.

The Cyclin-dependent kinase 8 (CDK8) is an early clinical stage drug that targets the overexpression in colon cancer.^396^ From Liang et al.^397^, inhibition of CDK8 almost has no effect on the growth of CRC cells and orthotopic transplanted tumors in subcutaneous, splenic, or cecum, but significantly inhibited the liver metastases of mouse and human colon cancer cells. CDK8 mediated CRLM due to downregulating of MMP inhibitor TIMP3 via TGFβ/SMAD-driven expression of a TIMP3-targeting microRNA, miR-181b, along with promotion of MMP3 in murine or MMP9 in human colon cancer cells via Wnt/β-catenin-driven transcription.^397^ This study illustrated that CDK8-targeting drugs can be used in the treatment of CRLM in the future.

We also would like to point out that the new development in cancer nanotechnology and photodynamic therapy (PDT) may provide potential solutions for CRC diagnosis and treatment.^398^ PDT has been exploited as a promising cancer treatment modality for many years. PDT can destroy cancer cells by generating reactive oxygen species (ROS) when photosensitizers are irradiated with light of suitable wavelength. PDT has the benefit of dual targeting by the drugs themselves and light, so its side-effects are much lower compared with Chemotherapy or radiation therapy. However, in traditional PDT, the light from the applied high-frequency source is scattered and cannot penetrate deep tissue, which limits its clinical application.^399^ Several possible solutions have been proposed to solve this problem, such as inventing new photosensitizers that use near infrared light and X-rays, and developing up-conversion nanoparticles.^400–402^ Among them, X-ray induced PDT has attracted much attention because of its unlimited tissue penetration ability.^398,403–406^ Most recently, Chen et al. reported Cu-Cy, Cu_3_Cl(SR)_2_ (R = CH_2_CH_2_NH_2_),^407,408^ a new type of sensitizer, has strong luminescence^407,409^ and can produce ROS under UV irradiation,^407,410^ X-rays,^411–413^ microwave radiation,^414–418^ and ultrasound.^419^ All these observations reported so far suggest that the Cu-Cy NPs are a new type of sensitizers with potential applications for infection inactivation^420^ and antitumor therapies.^421^ The most recent studies prove that this new Cu-Cy nanoparticle possess anti-tumoral effects and potential molecular mechanisms on B16 melanoma through X-PDT and X-ray induced antitumor immunity,^422^ and most importantly is that X-PDT is exceptionally effective with clinical X-rays used for irradiation in clinic settings.^423^ We believe that these new technologies could provide good solutions for CRC treatment.

## Perspective

CRC is still a common cancer and liver metastasis is the main cause of death in CRC patients. The treatment of advanced CRLM remains a major challenge. Previous studies have determined the main steps of the metastasis process. The role of TME, the dysregulation of NcRNAs, and the activation of various signaling pathways are closely related to liver metastasis. However, due to the lack of adequate experimental models to detect this complex process fully and continuously, the molecular mechanisms involved in the formation of CRLM have still much room for exploration. Detailed molecular mechanisms that mediate CRC metastasis to liver contribute to early detection and prevention. Future work should involve clarifying CRLM, based on the molecular mechanisms and clinical characteristics, which guide clinical precision treatment. At the same time, combined treatment and multidisciplinary cooperation should be established. Intervention at the early stage of liver metastasis, such as the stage of metastasis and colonization, will be more beneficial to improve patient survival. In addition, clinical and experimental data strongly suggest that postoperative tumor recurrence remains a possibility, therefore adjuvant targeted therapy is necessary for patients with postoperative resection. Defining high-risk factors for CRLM, accurate selection of high-risk individuals and minimizing the controllable risk factors are essential for the prevention of CRLM and further reducing mortality of CRC. It is also possible that the new development in cancer nanotechnology like nanoparticle self-lighting PDT may be a new hope for CRC treatment.

## Supplementary information

similarity index report
