# Supplementary material for: Colorectal liver metastasis: molecular mechanism and interventional therapy
Source: Signal Transduct Target Ther. 2022 Mar 4;7:70. doi: 10.1038/s41392-022-00922-2 (PMC8897452; doi:10.1038/s41392-022-00922-2)
Supplement: Supplementary file 1 — similarity index report [file 41392_2022_922_MOESM1_ESM.pdf]

STTT-Alice

By Chen-Alice Chen

WORD COUNT 13575
TIME SUBMITTED 25-JAN-2022 05:44PM
PAPER ID 81813326

Abstract Colorectal cancer (CRC) is one of the most frequently occurring malignancy tumors with a high morbidity additionally, CRC patients may develop liver metastasis, which is the major cause of death. Despite significant advances in diagnostic and therapeutic techniques, the survival rate of colorectal liver metastasis (CRLM) patients remains very low. CRLM, as a complex cascade reaction process involving multiple factors and procedures, has complex and diverse molecular mechanisms. In this review, we summarize the mechanisms/pathophysiology, diagnosis, treatment of CRLM. We also focus on an overview of the recent advances in understanding the molecular basis of CRLM with a special emphasis on tumor microenvironment and promise of newer targeted therapies for CRLM, further improving the prognosis of CRLM patients.

Keywords: CRLM; Molecular mechanisms; Targeted therapy; TME; Exosome; NcRNAs;

Introduction

Colorectal cancer(CRC) is one of the three most common cancers and the fourth most common cause of cancer deaths worldwide.[1] In 2020, 1.9 million cases were newly diagnosed with CRC and 935,000 cases with CRC past away.[2] The global incidence of CRC has been rising with annual increases of 3.2%, beginning with 783,000 cases in 1999 and increasing to 1.8 million in 2020[2-7]; this trend is likely to continue. Accordingly, the global CRC burden is increasing mainly owing to the growth of human development index (HDI)——The incidence rates in developing countries is a quarter of that in developed countries.[2]

Metastasis of CRC remains a major problem after curative treatment and is the critical cause of CRC-related death.[8] The liver is the most common organ of distant metastasis in CRC.[9] Liver metastasis of CRC may be associated with the following factors: the portal vein system directly connecting the colorectal and liver, which is associated with abundant blood supply; location and histological type of primary tumor.[10-12] Curative resection and chemotherapy are the standard methods treatment in patients with colorectal liver metastasis (CRLM),.[13] However, due to factors such as the location and size of the tumor, unresectable disease, presence of extrahepatic disease, or patients' comorbidities, surgery is only applicable in 10–20% of cases, with a five-year survival rate as low as 30%.[14, 15]

Furthermore, those who are not eligible for surgery have an even worse prognosis. Although, significant progress in the development of new chemotherapeutic drugs has been made, CRLM patients that receive fluorouracil and platinum chemotherapy initially, will eventually develop chemotherapy resistance due to inherent or acquired resistance. For CRLM, it is necessary to find more effective targeted therapies. Understanding of the molecular mechanisms underlined in this process could accelerate the achievement of this goal.

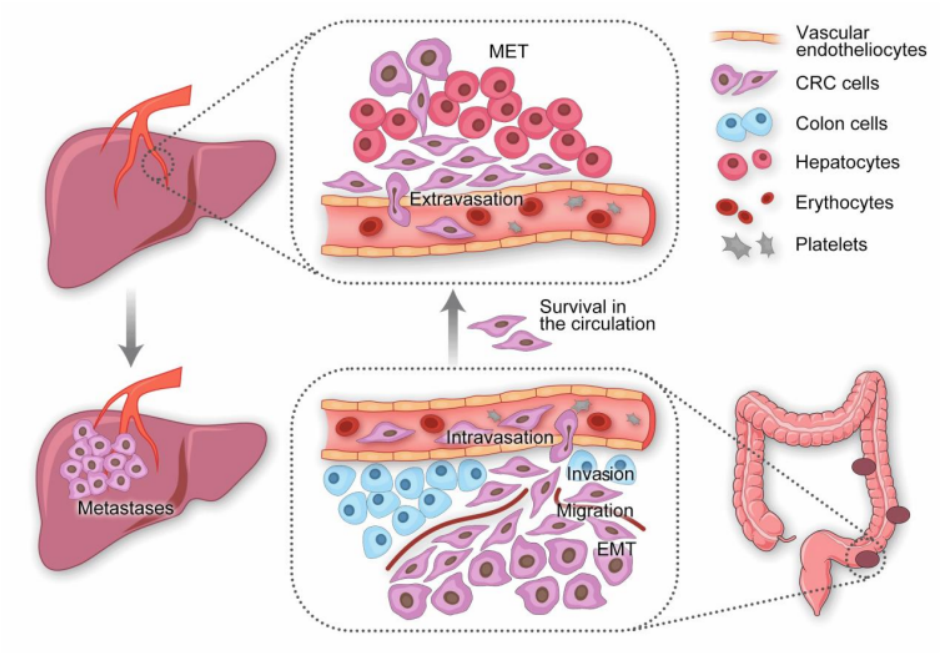

Fig. 1 Schematic showing the liver metastasis cascade of CRC

A small subset of CRC cells acquires a capacity to evade from the primary CRC, in part by morphological changes such as epithelial-to-mesenchymal transition (EMT), migration through the extracellular matrix (ECM), and invasion into the neighboring tissues, intravasation, survival in the circulation, extravasation and finally colonization to distant liver forming more aggressive secondary CRLM.

1. Epidemiology

Nearly 50% of patients with CRC will develop liver metastases during the disease.[16] The incidence of stage IV left colon cancer liver metastases is high.[17, 18] However, once liver metastasis occurred in right colon cancer, the range of liver metastasis was wider than that in left colon cancer.[19] The probability of liver metastasis in CRC is also associated with gender. Males are more likely to be a risk factor of CRC, with higher illness burden and earlier onset age[2, 4, 20] and 25%-50% of these patients develop liver metastasis, of which approximately 30% are diagnosed with CRC at the same time. Notably, ethnicity was likely to be correlated with the incidence of development of CRLM. Research in the United States alone reported that non-Hispanic black patients have the highest incidence of liver metastasis, followed by American Indian/Alaska Native, non-Hispanic white, Hispanic and Asian/Pacific Islanders.[23] That is, the variations in genes might contribute to the CRLM. Prior works have suggested that BRAF, KRAS, NRAS, PI3KCA, TP53, NRAS, CDK12, EBF1 might be risk genes of CRLM.[24-27] Furthermore, the types of gene mutations were also likely to be correlated with the prognosis[27]——the highest cure rates appeared in patients with NOTCH1 and PIK3C2B mutations, and the lowest in those with SMAD3 mutations.

2.Mechanisms/pathophysiology

In 1889, Paget proposed the concepts of "seed" (tumor cells) and "soil" (specific organs) for tumor metastasis.[28] The liver metastatic cascade of CRC is a complex multi-factor and multi-step biological process (Fig. 1), wherein a small subset of CRC cells acquire a capacity to evade from the primary CRC, in part by morphological changes such as EMT, migration through the ECM, invasion into the neighboring tissues, intravasation, survival in the circulation, extravasation and finally, colonization to distant liver forming more aggressive secondary CRLM.[8, 29] On one hand, this process is related to genome abnormalities in the tumor cells themselves, including activation of proto-oncogenes and inactivation of tumor suppressor genes. The occurrence and development of CRLM is especially complex and involves many molecular mechanisms, including non-coding RNAs(ncRNAs), Notch pathway, TGFβ signaling, Tyrosine kinase c-MET signaling， phosphatase of regenerating liver (PRL3), tumor-associated calcium signal transducer 2(Trop-2), L1 cell adhesion molecule (L1CAM)， S100 family proteins S100A4 and S100A8 and other pathways (Table 1). It is also closely related to the tumor microenvironment (TME), which mainly involves various immune cells (macrophages, T

cells, B cell etc.), cytokines, chemokines and exsomes.[30-32] The interaction of internal and external environment jointly initiates and drives the occurrence of CRLM.

2.1 Immune cells

Immune microenvironment plays a pivotal role in CRLM.[33] The liver is an important immune organ of the human body. If its immune killing ability is weakened, it can promote the survival and growth of CRC metastases. Therefore, a suppressive immunologic microenvironment plays tumor-promoting role of CRLM, which is related to tumor associated-macrophages (TAMs) and regulatory T cells (Treg). However, TAMs maintains the immunosuppressive environment by expressing checkpoint ligand programmed death ligand 1 (PDL1), PDL2 and other inhibitory receptors[34], and activates Treg cells by secreting IL-10 and TGF β .[35] TAMs also release a plethora of ECM remodeling factors (plasminogen activation system, matrix metalloproteinases, and kallikrein-related peptidases) and a diverse array of proteolytic enzymes such as matrix metalloproteinases (MMPs) that degrades the ECM proteins. These factors, in turn, enhance migration of tumor cells.[36] In addition, when targeting the CCL2/CCR2 chemokine axis, TAMs infiltration at the metastatic site is reduced and mCRC is sensitized to tumor T cells.[37]

Meanwhile, Treg cells inhibit aberrant immune response against self-antigens and anti-tumor immune response.[38] The ability to inhibit adaptive anti-tumor immune responses via Treg is associated with clinical outcomes of CRLM.[39] The intratumoral Treg could inhibit MMPs expression

and activity with IL-17 producing T cells involved which reduces the probability of postoperative metastasis of CRC.[40] Inhibition of Treg activity may be a therapeutic approach to improve anti-tumor immunity in the future.[39]

The tumor-associated neutrophils (TANs) may also promote tumor growth and metastasis through a variety of mechanisms.[41] Hyperlipidemia can promote neutrophil infiltration, thereby increasing the metastasis of CRC.[42] In the early phase of CRC cell dissemination, neutrophils expressed CCR1 exclusively and MMP9 preferentially, which contribute to the early expansion of cancer.[43] TANs produce large amounts of lysine oxidase-like 4 protein during CRLM resistance anti-angiogenic therapy.[44] TANs could be recruited by loss of SMAD4 promoting chemokine CCL15 expression via the CCL15-CCR1 axis.[45] In addition, neutrophil extracellular traps (NET) trigger the release of HMGB1 and promote the migration and invasion of cancer cells during stress response.[41] NETs could also directly trap CRC cells in liver boosted tumorous proliferation and invasion capacity, which is because of the heightened expression of tumorous interleukin (IL)-8 which is triggered by NETs.[46] Interestingly, overproduced IL-8 in turn activated neutrophils towards NETs formation, thus forming a positive loop enhanced CRC liver metastasis. Therefore, eliminating NET may reduce risks of tumor relapse after surgical stress. Recently, Xia et al.[47] reported neutrophil infiltration and NETs formation were inhibited in tumor tissues with Adeno-associated virus (AAV) -mediated DNase I liver gene transfer treatment. NET-associated carcinoembryonic Ag cell adhesion molecule 1 (CEACAM1) as an essential element for inducing CRC metastatic phenotype, which prompted CEACAM1 as a potential therapeutic target for the prevention of CRC metastasis.

Myeloid-derived suppressor cells (MDSC) are another key factor that regulates the immune response under many pathological conditions, and immune suppressive activity is an intrinsic feature of MDSC. Neutrophils or monocytes that has undergone maturation is not able to transform into potent immune suppressive cells in vitro when simply activating MDSC with hazard associated molecular patterns (DAMPs) and pathogen associated molecular patterns (PAMPs) or pro-inflammatory cytokines.[48] CCR1(+) MDSC are recruited to the microenvironment of disseminated CRC cells by loss of SMAD4 promoting chemokine CCL15 expression via the CCL15-CCR1 axis, and produce metalloproteinases MMP9 and MMP2 to enhance metastatic colonization.[43, 49] S1PR1–activator of

transcription 3 (STAT3) upregulation in CRC cells promotes IL-6, which could activate S1PR1–STAT3 in MDSC in the liver, leading to premetastatic niche formation before CRC cell arrival.[50]

There has been some progress regarding the immune status of CRLM and the effect of tumor immunological characteristics on clinical outcome in CRLM patients. The density of macrophages in CRC is also closely related to patient prognosis.[51] The CRLM T cell number is an independent correlate of long-term survival following liver resection, in particular, CD8+ T-cell infiltration in liver metastasis is associated with better prognosis.[52-54] Moreover, high frequencies of NK and T cells in response to chemotherapy predict OS in CRLM patients.[55]

2.2 Cytokines

Cytokines IL-6 active IL-6 receptor (IL-6R), which induce STAT3 to bind to MIR34A inhibit miR-34A expression, thereby promoting EMT-mediated invasion and metastasis of CRC.[56] Growth differentiation factor 15 (GDF15) in inflammatory microenvironment induces CRC metastasis by regulating EMT genes by activating c-Fos, which may be a new direction for CRLM treatment in the future.[57] Similarly, cytokines IL-33 modulates the TME to potently induce the occurrence of liver metastasis.[58] In addition, the levels of arachidonic acid (AA) and eicosapentaenoic acid (EPA) promoted the development of an inflammatory microenvironment and AA/EPA ratio was elevated levels in patients with metastatic CRC.[59]

2.3 Chemokines

Chemokines, as an important component of TME, play an important role in the entry of tumor cells into the TME.[60] For instance, chemokines are critical for the tumor–stroma interaction, which promote tumor metastasis via chemokines signaling. Chemokines could also act as a bridge between the microenvironment outside the tumor and the tumor itself, and their cognate receptors are expressed by both tumor and stromal cells.[61] In recent years, it has been reported that chemokine ligands and receptors (such as CXCL5, CCL3, CCL4, CXCL2, CXCL3, CXCL8, CCL3L3, CCL4L2, CCL18) have been significantly dysregulated, which implies a potential intercellular communication in the immune microenvironment of CRC. Chemokine ligand-receptor interactions between different

cells further complicate the signal transduction in CRLM (Table 1).

Table 1. Chemokine ligand-receptor interactions between different cells further complicate the signal transduction in CRLM.

Chemokine Signaling	Ligand	Receptor	Function	Refs.
CCL9-CCL15	CRC	CD34(+) Gr-1(-) iMCs	Liver metastasis	[62]
CCL15-CCR1	CRC	CD11b(+), CD33(+),HLA-DR(-)MDSCs	Liver metastasis	[62 49]
CCL2-CCR2	CRC	CD11b(+)Gr-1(+) iMCs	Liver metastasis	[63, 64]
CXCL1-CXCR2	CRC	CXCR2+microvascular endothelial cells	tumor angiogenesis	[65, 66]
CXCL1-CXCR2	CRC	CXCR2+ macrophages	Liver metastasis	[67]
CXCL1-CXCR2	CRC	CXCR2+ neutrophil	Liver metastasis	[67]
CXCL1-CXCR2	TAMs	CXCR2+ MDSCs	Liver metastasis	[68]
CCL2-CCR2	CRC	TAMs	Liver metastasis	[37]

CXCL8-CXCR2	TAMs	CXCR2+ neutrophil	Liver metastasis	[69]

As a member of the CXC chemokine family, CXCL5 is the ligand of CXCR2 and is not only derived from primary tumor cells but is also secreted by immune cells in the TME.[70]

The CXCL5/CXCR2 biological axis promoted tumor angiogenesis by activating the AKT/NF-κB/FOXD1/VEGF-A pathway.[71] Moreover, Zhao et al.[72] found the elevated expression of CXCL5 in CRC induced cell migration by the ERK/Elk-1/Snail signaling pathway and promoted cell invasion through the AKT/GSK3β/β-catenin/MMP7 signaling pathway. They also found that high expression of CXCL5 was a favorable factor to promote the metastasis of CRC cells to the liver in nude mice intrasplenic injection model.

The earlier study confirmed that CCR1+ immature myeloid cells (iMCs) could migrate toward CRC-derived CCL9 and accumulate at tumor invasion front in mice model.[73] Later studies demonstrated CRC cells secrete CC-chemokine ligands CCL9 and CCL15, respectively, and recruit CD34(+) Gr-1(-) iMCs to the metastatic liver.[62] Additionally, CRC-derived CCL2 and Myeloid-derived S100A8/A9 enhanced CD11b(+)Gr-1(+) iMCs recruitment and increased tumor burden via CCL2/CCR2.[63, 64] Also, CRC-derived CXCL1 increased migration of CXCR2+microvascular endothelial cells in vitro and mediated tumor angiogenesis by binding with CXCR2 receptor on endothelial cells in xenograft CRC models.[65, 66] Moreover, CXCL1 is found to recruit CXCR2+ neutrophil and macrophages from the bone marrow to premetastatic liver in CRC nude mice.[67] It was also reported that CCL15 secreted by CRC attracted CCR1(+) MDSCs to the pre-metastatic niche via the CCL15–CCR1 axis，creating conditions conducive to metastasis.[49] TAMs also recruited CXCR2-positive MDSCs to promote liver metastases via producing CXCL1.[68] High expression of CCL4 in CRC could induce the infiltration of TAMs and specifically a pro-tumor macrophage profile (CD163+ cells).[74] CCL2/CCR2 chemokine axis could also facilitate TAMs accumulation in liver metastasis and maintain immunosuppressive TME. In addition, CXCL8 and its receptor (CXCL8-R) promote liver metastasis by promoting angiogenesis and inducing EMT in CRC cells.[75] Elevated

serum CXCL8 levels are associated with poor prognosis in patients with CRC.[45, 69]

In clinical CRC specimens, CX3CR1, the chemoattractant cytokine CX3CL1 receptor, was expressed on TAMs and in a microenvironment lacking CX3CR1, the liver metastasis of CRC cells was significantly inhibited.[76] However, the high expression of CX3CR1 promoted the increase of T cell invasion in tumor tissue, and thus inhibited tumor growth.[77] Low-molecular-weight heparin, a common drug for venous thromboembolism, inhibited the formation of liver metastasis of CRC by disrupting the interaction of CXCR4 and CXCL12.[78] Stromal cell-derived factor 1alpha activated CXCR4-expressing CRC cells, then resulted in a significant increase of cell migration.[79] Moreover, hepatic stellate cells (HSCs) play important role in liver metastasis of CRC cells by the action of SDF-1/CXCR4 axis.[79]

2.4 The emerging roles of exosomes in CRLM

Like chemokine, exosomes also built crosstalk between various cells. Exosomes are lipid bilayer vesicles with a diameter of 30-100 nm.[80] Exosomes are very widespread and are found in almost all body fluids.[81] Cancer patients have more circulating exosomes than non-cancer patients.[82] Exosomes can also mediate colonic epithelial-stromal interactions involved in the regulation of tumor growth and metastatic invasion.[83] Exosomes play a key role in building a supportive microenvironment in the metastatic organs, namely the pre-metastatic niches (PMNs), which include vascular leakiness, inflammation, and immunosuppression.[84] We review the role of exosomes in CRLM development, and the clinical application of exosomes (Fig 2 & 3).

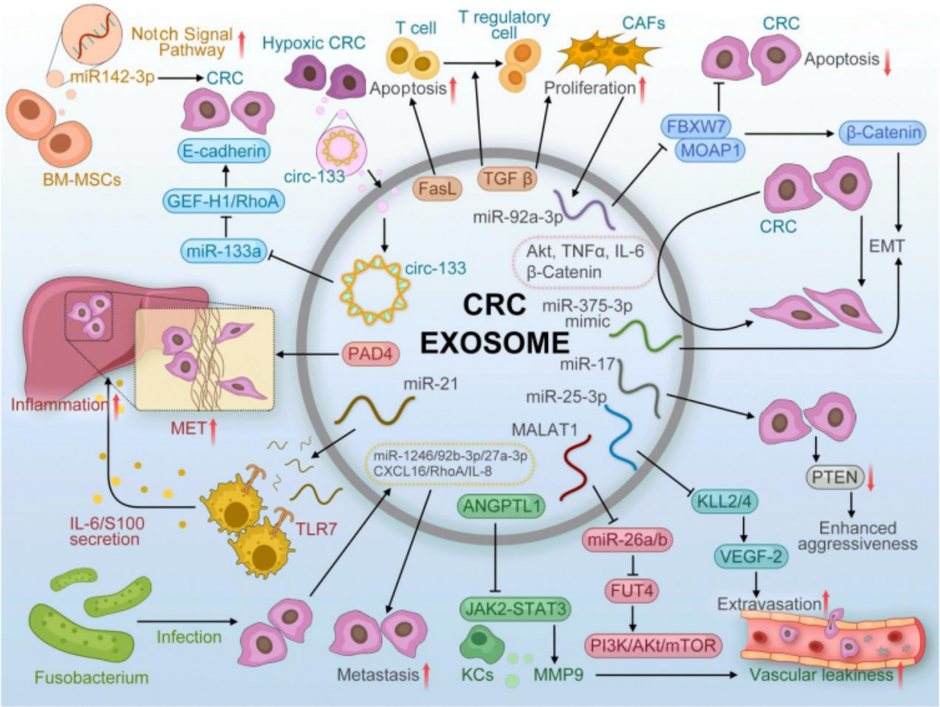

Fig 2. CRC-Derived Exosomes Content and Mechanisms for CRLM

CRC-derived exosomes contain various biomolecules such as protein (PAD4，FasL, TGFβ，ANGPTL1，AKt，TNF-α，β-catenin), RNA (miR-246，miR-21，miR-25-3p，miR-375-3p，miR-92b-3p，miR-27a-3p，miR-17，MALAT1). These biomolecules establish pre-metastatic niches in liver and induce proliferation, inflammation, EMT, invasion, migration and EMT, thereby promoting CRC metastasis.

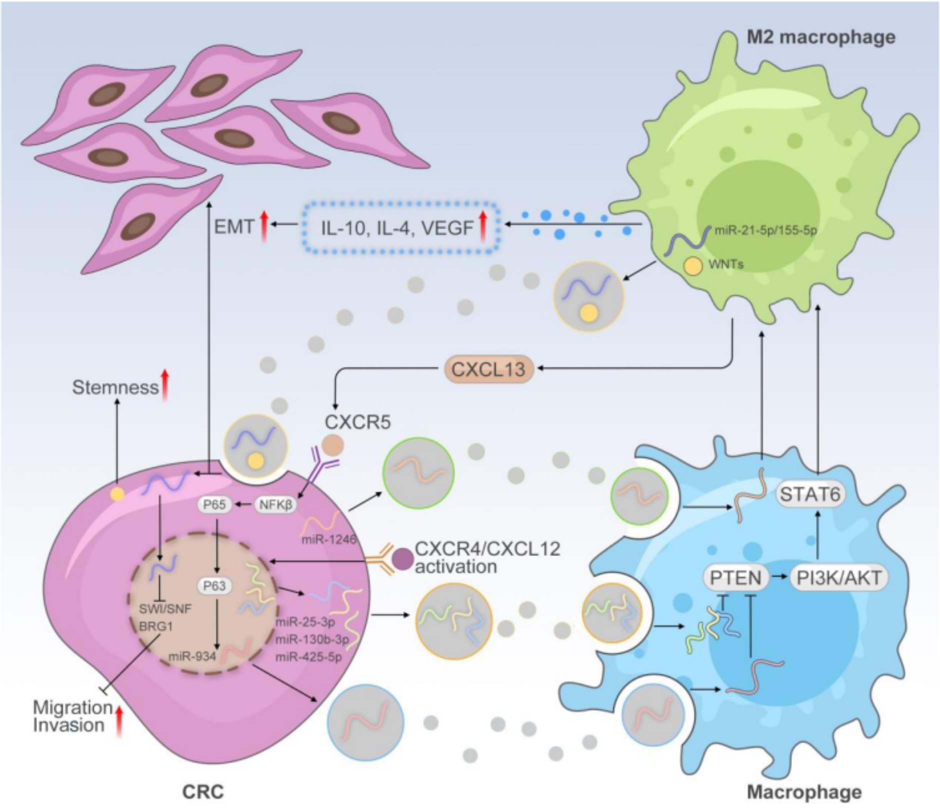

Fig 3. Schematic illustration the exosomal interaction between CRC cells and TAMs that reveals the molecular mechanism of CRLM

The Complicated network of the exosomal interactions among the CRC cells and TAMs promotes metastasis. CRC induces macrophages to M2 macrophages in TME via secreting exosomal miRNA(miR-934,miR-25-3p,miR-130-3p,miR-425-5p and miR-1246). M2 macrophages also promoted CRC EMT and maintained stemness by exosomal miR-21-5p and WNTs. Moreover, CXCR5, IL-10,IL-4 and VEGF were also the medium of communication between CRC cells and TAMs.

2.4.1 The substances in exosomes

The substance in exosomes includes proteins, miRNAs, lncRNAs and mRNAs, which can spread in the circulatory system and play an important role in CRLM. Angiopoietin-like protein 1 (ANGPTL1) , a tumor suppressor, was decreased in CRC tissue.[85] Exosomal ANGPTL1 decrease MMP9

production in Kupffer cell (KCs) by suppressing the JAK2-STAT3 signaling pathway.[86] Tumor cells can transfer EMT inducers, such as IL-6, Akt and tumor necrosis factor-α (TNF-α), through exosomes to induce EMT in neighboring tumor cells.[87]

In addition to protein, miRNAs are also a common substance in exosomes. Circulating exosomal miRNA can be used to assist in the diagnosis or prognosis evaluation of CRC patients. Circulating miRNAs are mainly derived from exosomes miRNA.[88] Therefore, the analysis of circulating exosomal miRNAs can promote the early metaphase diagnosis rate of CRLM and intervention in the metastatic process. Zeng et al.[89] found that exosomal miR-25-3p secreted by CRC cells was transferred to vascular endothelial cells and promoted CRC metastasis by targeting Kruppel-like factor 2 (KLF2) and Kruppel-like factor 4 (KLF4) to increase vascular permeability and promote angiogenesis in animal models. The clinical data further showed that miR-25-3p from circulating CRC-derived exosomal miR-25-3p can be used as a biomarker to predict metastasis. Similarly, CRC-derived exosomes packed with miR-21-5p created a liver proinflammatory phenotype and liver metastasis of CRC via the miR-21-Toll like receptor7(TLR7)-IL-6 axis.[90] Metastasis-associated miR-106b-3p from serum exosomes targeted deleted in liver cancer-1 (DLC-1).[91] Elisabetta et al.[92] reported that exosomal miR-210 can perhaps be reckoned as an EMT signal promoter which maintain the local cancer-growth milieu and influence the adhesion and migration of CRC cells. Moreover, Matsumura et al.[93] showed that six exosomal miRNAs (miR-19a, miR-19b, miR-4437, miR-23a, miR-320a and miR-92a) were related to the liver metastasis. Interestingly, miR-375 mimicThe tumor-derived exosome containing miR-375 mimic that could inhibit the EMT process.[94] According to Fu et al.[95] the high expression of miR-17-5p and miR-92a-3p were consistent with the tumorigenesis and metastasis of CRC by exploring serum exosomal miRNAs levels of normal controls and CRC patients. Interestingly, exosomal miR-1246/92b-3p/27a-3p derived by fusobacterium nucleatum-infected CRC cells facilitate uninfected cells metastasis.[96] Recently, Zhang et al.[97] reported the secretion of colorectal cancer exosome miR-1255b-5p was decreased under hypoxia, thereby promoting human telomerase reverse transcriptase inhibition to enhance EMT and telomerase activity.

Moreover, exosomal miRNAs are secreted by cancer associated fibroblasts (CAFs), then they are

transferred to CRC cells.[98] Mechanistically, miR-92a-3p inhibits mitochondrial apoptosis by activating the Wnt/β-catenin pathway and inhibiting FBXW7 and MOAP1, thereby enhancing stemness, EMT, metastasis and 5-FU/L-OHP resistance of CRC cells.[98] Reducing exosomal miR-92a-3p may contribute to the prediction and treatment of CRLM. CRC cells can also induce CAF via exosomal TGF-β.[99, 100] Moreover, exosomes miR-142-3p secreted by bone marrow-derived mesenchymal stem/stromal cells(BM-MSCs) inhibited Numb expression in CRC cells, thereby increasing the population of CSCs.[101]

MiRNAs in exosomes isolated from CRLM are different from the profile generated from an orthotopic cecum tumor model and naïve colon tissues because higher levels of tumor suppressor miRNAs are encapsulated in the exosomes in more advanced disease.[102]The level of oncogenic miR-21 was much higher in the primary colon tumor tissue and metastatic colon tumor in the liver than in their exosomes. However, the level of tumor suppressive miR-18a and miR-193a in the exosomes is enhanced.[102] The serum levels of exosomal miR-200c and miR-141 were proved to predict CRC patients with poor prognosis.[103] The monitoring of CRC metastasis was monitored in real time based on miR-139-3p content in the plasma of CRC patients.[104] Similarly, in CRC patients with LM, the serum exosomal miR-122 can serve potentially as both a novel diagnostic and prognostic biomarker.[105] A recent study provides a new concept that miR-25-3p, miR-130b-3p， miR-425-5p, miR-193a, let-7g ,miR-106b-3pand miR-934 contained in exosomes could participate in progression and metastasis of CRC.[91, 106-108] Thus, all the evidences point out that exosome-mediated promotion of tumor progression, and understanding these mechanism-based circulating exosomal miRNAs will reveal new avenues for future diagnosis and treatment.

Furthermore, the exosome-derived lncRNAs could play essential roles in tumorigenesis by regulating the TME.[109] Exosomal lncRNAs BCAR4 could be potential candidates to detect CRC.[110] Exosomal MALAT1 regulated FUT4 expression by sponging miR-26a/26b to promote CRC progress.[111] CRC cells secreted exosome cirC-133 into relatively normoxic CRC cells under hypoxic conditions. Then circ-133 adsorbed miR-133a to target GEF-H1/RhoA, as a result of reducing the distribution of E-cadherin on the membrane.[112] Guo et al.[113] reported fusobacterium infection may stimulate CRC cells

to generate CXCL16/RhoA/IL-8 exosomes that are delivered to uninfected cells to promote prometastatic behaviours.

2.4.2 The exosomal interaction between CRC cells and TAMs

There is also a noticeable interaction between CRC cells and TAMs via exosomes. Exosomes secreted by M2 macrophages promoted CRC migration by exosomal transfer of miR-21-5p and miR-155-5p.[114] Mechanistically, these miRNAs mentioned above targeted the core component of the Switch/sucrose non-fermentable (SWI/SNF) complex BRG1. Moreover, M2 macrophages -derived exosomes could induce CRC stem cell activity because of containing WNT.[115] CRC cell-derived exosomal miR-934 induced M2 macrophage polarization via downregulating PTEN expression and activating the PI3K/AKT signaling pathway.[107] Polarized M2 macrophages secreted CXCL13, then activated a CXCL13/CXCR5/NFκB/p65/miR-934 positive feedback loop to induce premetastatic niche formation in CRC cells. Exosomal miRNAs miR-25-3p, miR-130b-3p, miR-425-5p secreted by CRC cells via activation of the CXCL12/CXCR4 axis, could be transferred to macrophages which can then target PTEN and shift the macrophage towards M2 phenotype.[106] Additionally, mutant p53 CRC could reprogram macrophages into M2 macrophages by transferring exosomal miR-1246.[116]

2.4.3 The exosomal interaction between CRC cells and other types of immune cells

CRC-exosomes containing transferring tumor growth factor-β (TGF-β) can activate TGF-β/Smad signaling and inactivate SAPK signaling which induces phenotypic alteration of T cells to Treg-like cells.[117-119] Delivery of Fas-ligand-containing CRC cell exosomes to T cells can induce cell apoptosis.[120] Citrullination generated by peptidylarginine deiminase 4 (PAD4) derived from CRC cells as a driver of liver metastases.[121] In another study, it was reported that mutated KRAS is transferred to recipient cells through exosomes and induces increased IL-8 production, recruiting more neutrophils and further aggravating CRC.[122]

2.5 Key molecules and signaling pathway driving liver metastasis

Various signaling pathways and factors may be involved in the process of CRLM, including

hepatocyte growth factor/c-Met(HGF/c-Met) signaling pathway, phosphatase of regenerating liver (PRL3), Notch pathway, TGFβ signaling, Tyrosine kinase c-MET signaling, tumor-associated calcium signal transducer 2(Trop-2), L1 cell adhesion molecule (L1CAM), MACC1, S100 family proteins and other pathways. There are multiple intersections between these molecular mechanisms driving CRLM.

2.5.1 HGF/c-Met signaling pathway

HGF/c-Met signaling pathway promotes metastasis of cancer cells by regulating a diverse downstream prometastatic effector molecules, then overactivated phosphatidylinositol-3-kinase (PI3K) and mitogen-activated protein kinase (MAPK) signaling(Fig. 4).[123-125] Met, a receptor of hepatocyte growth factor (HGF), has a positive correlation with tumor stages of CRC liver metastasis. High expression of circulating HGF and aberrant activity of cMet were detected in patients with CRC.[126] Inhibition of HGF/ C-MET signaling pathway could decrease the proliferation and invasion of CRLM.[127] By assessing the patient tissue samples, Yao et al.[128] reported higher levels of c-Met expression (mRNA and protein) in CRLM than primary CRC by assessing the patient tissue samples. HGF/c-Met could regulate urokinase plasminogen activator (uPA) and uPAR in vitro CRC migration and invasion models.[129] Ectopic TIMP1 expression provoked pro-metastatic microenvironment in the liver by inducing the HGF/c-Met signaling as well as uPA expression.[130, 131] Inhibition of ADAM-10 was in principle able to prevent shedding of c-Met, which may be one explanation for the increase of cell-associated c-Met in livers with elevated TIMP-1.[130] Indeed, in livers of uPA-ablated mice upregulated TIMP-1 expression did not trigger HGF/c-Met signalling.[131]Moreover, elevated levels of TIMP-1 in the TME could induce metastasis by enhancing HIF-1α-dependent HGF-signaling.[132]

In addition, SOX13 is induced by HGF through JAK2/STAT3 signaling and then upregulated SOX13 transactivates the expression of c-MET by directly binding to its promoters, which contributes to c-MET overexpression in CRC.[133] This feedback loop induces SOX13-mediated CRC migration, invasion, and metastasis. Similar to SOX13, Forkhead box protein C2 (FOXC2) was directly associated with c-MET promoter to increase the transcriptional activity of MET.[134] Blocking KPNB1(a novel gene) expression showed a significant inhibitory role in metastasis both in

vitro and in vivo through interacting with MET proto-oncogene. COX-2/PGE(2) pathway as an important mediator of HGF/Met signaling is closely associated with the survival, proliferation and invasion of CRC cells.[135] HGF/Met signaling is an important regulator of the COX-2/PGE 2 pathway in CRC cells, stimulating PGE 2 synthesis via COX-2 up-regulation and inhibiting PGE 2 degradation via Ras-MAPK/ERK while PI3K/AKT signaling mediate HGF-driven 15-PGDH down-regulation.[136] In fact, IL-6/IL-6R, HGF/c-Met, STAT3, VEGF cytokinetic pathway is a major mechanism of the pro-oncogenic effect induced by hepatic RFA.[137]

The association between C-Met and colorectal cancer was even stronger due to c-MET being identified as a transcriptional target of colon cancer metastasis related gene 1 (MACC1). [138] MACC1 promotes proliferation, invasion, and HGF-induced scattering of CRC cells in cell culture and tumor growth and metastasis in mouse models. In advanced metastatic CRC patients, MACC1 and c-Met were both upregulated.[138] MACC1, a new detectable biomarker in cancer, is also an independent prognostic factor for the recurrence after liver resection of CRC metastasis.[139]

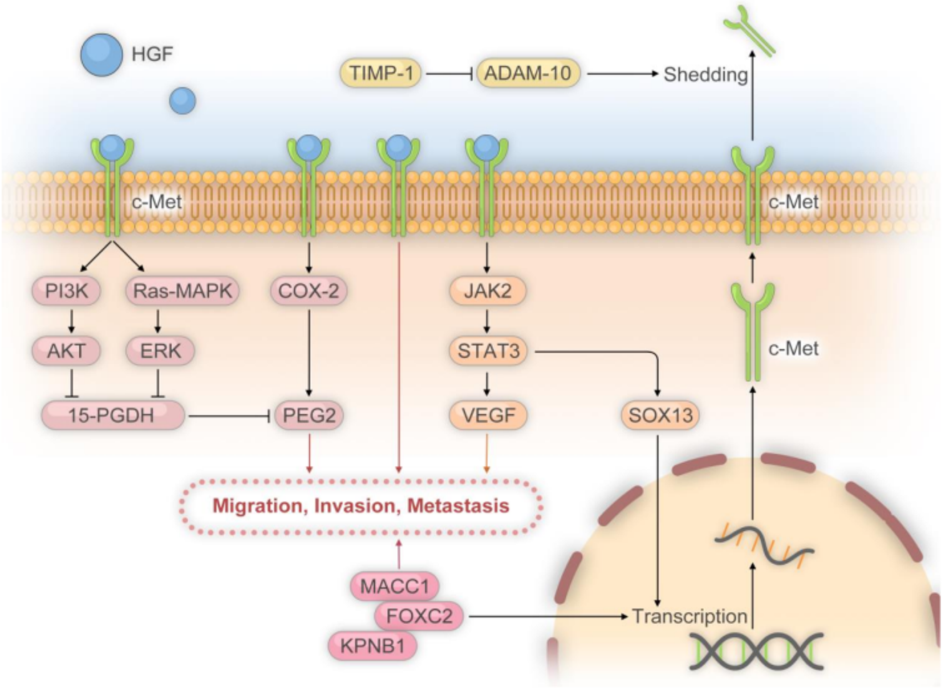

Fig 4. HGF/c-MET signaling pathways and its role in cellular activity

HGF/c-Met signaling promoted metastasis of cancer cells by regulating a diverse downstream prometastatic effector molecules, via Ras-MAPK/ERK， PI3K/AKT signaling, JAK2/STAT3 signaling. MACC1, KPNB1 and FOXC2 could transactivate the expression of c-MET.

2.5.2 CRLM and PRL3

PRL3 has received widespread attention as a potential cause for metastasis (Fig. 5).[140] Since PRL3 transcripts are overexpressed in CRC metastases found in the liver, and PRL3 expression cannot be detected in non-metastatic primary tumors and normal colorectal epithelium. PRL3-induced enhancement of EMT dependent on EGFR activation and PRL3 promoted cell invasion and upregulated MMPS by activating AKT in vitro and in vivo.[141-144] The levels of PRL-3 mRNA expression can be used as biomarkers for increased risk of liver metastasis.[145] The PRL-3 expression did not represent a direct causative mechanism of liver metastasis, but modulated multiple signaling pathways, including PI3K/AKT and MAPK/ERK in various cancer cells. It was reported liver metastasis by PRL-3 is mediated through lymph node metastasis and elevated tumor markers(CEA and CA19-9) in the serum.[146] Overexpression of PRL-3 can also promote the proliferation and invasiveness of CRC cells, mainly by activating STAT3 to increase the expression of miR-17, miR-19a and miR-21.[147] There is evidence that ubiquitin specific protease 4 (USP4) drives CRC invasion and metastasis via binding with and deubiquitinating PRL-3 to stabilize PRL-3.[148] Moreover, PRL-3 improved IL-8 secretion in CRC cells and mediated enhancement of glycolysis, which in turn contributed to the promotion of cancer metastasis.[149] PRL-3 also promoted CRC invasion and metastasis by upregulating chemokine ligand 26 (CCL26) to induce cancer metastasis.[150] Recently, it was shown that PRL-3 promoted cell metastasis via activating MAPK pathways in TAMs to initiate EMT, and the NF-κB pathway activated by PRL-3 contributed to angiogenesis in CRC cells.[151]

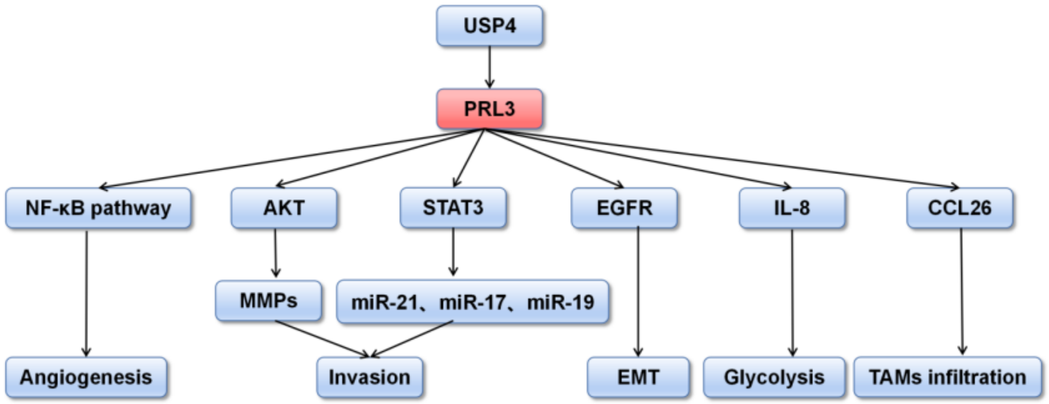

Fig 5. The roles of PRL3 in cellular activity

PRL3 promoted metastasis of cancer cells by regulating a diverse downstream prometastatic effector molecule, via NF-κB pathway, AKT, STAT3, EGFR, IL-8 and CCL26.

2.5.3 CRLM and Notch pathway

Furthermore, there are other signaling pathways that are related to CRLM. The Notch pathway plays a positive role in CRLM and inhibition of Notch signaling interferes with CRLM.[122] The human Notch system includes four Notch receptors (Notch1-4) and five Notch ligands (Jagged1-2, DLL1, DLL3, DLL4).[152] Activation of the Notch pathway is related to the poor prognosis of CRC. Notch1 gene copy number amplification may indicate a decrease in patient survival.[153] The positive expression of Notch3 protein is an unfavorable prognostic factor of disease-free survival (DFS) and overall survival (OS).[154] However, the functions of notch2 and notch4 are opposite to them, their overexpression can block the proliferation, invasion or migration of cancer cells. [155, 156] Meanwhile, multiple Notch signaling pathway related mechanisms play a significant role in metastatic CRC(Fig.6)

The enhanced aggressiveness of colorectal tumors caused by Notch signal activation may be related to EMT. Retroviruses are used to construct colorectal tumor cells that can stably express Notch-1 intracellular domain (NICD-1) in the cytoplasm, and the Notch ligand Jagged-1 and CD44, Slug, Smad-3 and other EMT-related proteins are found to be up-regulated in these CRC cells.[157] Further

treatment of the cells with γ-secretase inhibitor DAPT can effectively inhibit this process. Interestingly, NICD-1 produced by retroviruses is not affected by DAPT, which suggests that this may be caused by a more complicated mechanism-after the activated Notch1 receptor releases NICD, the expression of the ligand Jagged1 is upregulated. Animal experiments have shown that when Notch/TGF-β2 signaling is inhibited, it is accompanied by a decrease in neutrophils in the target organs of metastasis, as well as the gradual accumulation of CD3 +, CD4 + and CD8 + T cells, resulting in a significant reduction in metastasis.[158] In addition, in the EMT-related literature of other types of cancer, it has also been reported that hey-1, an important downstream target gene of the Notch signaling pathway, can regulate the TGF-β/Smad signaling pathway, and the TGF-β-dependent signaling pathway is closely related to the induction of EMT phenotypes.[159] This indicates that the crosstalk of these two signaling pathways may play a more essential role in the process of colorectal tumor metastasis.

Song et al.[160] confirmed that autocrine motor factor receptor (AMFR) and notch1 are the direct target genes of miR-139-5p in colorectal tumors, up-regulation of miR-139-5p promotes the expression of AMFR and NOTCH1 to enhance the migration and metastasis of colorectal cancer. In addition, knockdown of Notch ligand Jagged1 can lead to a decrease in Notch signal transduction activity of CRC cells, thereby reducing the migration of CRC cells. Moreover, in Jagged1 silenced nude mouse xenograft tumors, tumor metastasis markers MMP-2 and MM-9 are also significantly down-regulated.[161] Ligand DLL4 can up-regulate MUSASHI-1 (MSI-1) through a Notch3-mediated mechanism, while MSI-1 inhibits the translation of Notch signaling negative regulator NUMB, and then reduces the degradation of NICD in tumor cells, and promotes the activation of Notch signaling pathway.[162] In addition, Amino-terminal Enhancer of Split (AES) can inhibit the metastasis of colorectal tumors by lowering Notch signal.[163] In the past, there was little knowledge about the regulation of AES expression. The latest research found that it may be affected by the human Casein kinase 1δ/ε (CK1δ/ε), meanwhile CK1δ/ε is closely related to Wnt signal and Hedgehog signal in the occurrence and development of CRC.[164]

Ectopic expression of tRNA-derived fragments (tRF)/miR-1280 reduced cell proliferation and

metastasis by directly inhibiting the Notch signaling via targeting JAG2, leading to the decreased activity of Notch pathway components and Notch signaling.[165] Moreover, the intensity of Notch1 expression was related to depth of invasion, TNM staging and lymph node metastasis of CRC.[166] Inhibition of tumor-derived Tumor-Derived Laminin α5(LAMA5) activated the Notch pathway in tumor endothelial cells, thereby reducing branching angiogenesis.[167]

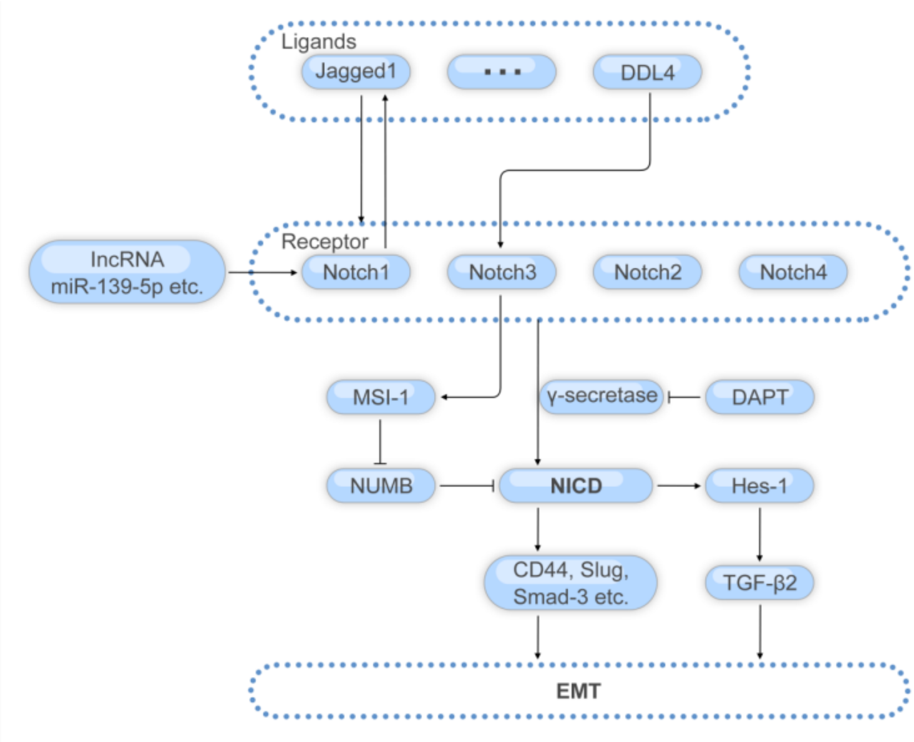

Fig 6. Schematic illustration of Notch pathway regulatory mechanisms.

MSI-1, MUSASHI-1; CK1δ/ε, Casein kinase 1δ/ε ; NICD, Notch intracellular domain; EMT, epithelial-to-mesenchymal transition.

2.5.4 CRLM and TGFβ signaling

The TGF-β superfamily signaling contains more than 30 members, among which the more important ones are TGF-β, Activins, Nodal, Bone Morphogenetic Proteins (BMPs), etc.[168] In mice with progressive liver metastatic disease, blockade of TGFβ signaling rendered tumors susceptible to anti-

programmed cell death 1(PD1) therapy.[169] TGF-β1 could also downregulate the E-cadherin expression and increase the Vimentin expression, inducing EMT to promote the invasion and migration of CRC.[170] Moreover, CRC-derived CXCR4 activated hepatic stellate cells (HSCs) to release SDF-1, resulting in TGF-β1 secretion in CRC cells to promote liver metastasis of CRC.[171] Therefore, TGF-β1 signaling blockage may be an effective clinical strategy for CRCM.

TGF-β plays a dual role in the tumorigenesis process. In the early stage of tumorigenesis, TGF-β plays a role in inhibiting tumors. However, in the later stages, TGF-β can promote EMT and is closely related to tumor invasion and metastasis.[172] In CRC, CMS4 type has a higher degree of TGF-β activation.[173] Studies have shown that TGF-β1 can promote the occurrence of EMT by activating a variety of transcriptional regulators including SNAI1/2, Twist and ZEB1/2, etc.[174] Increased expression of TGF-β1 can increase the metastasis of CRC cells, leading to the secretion of IL-11 by CAFs to trigger the STAT3 signaling pathway.[175]

Equally important, SMAD4 is also an essential molecule in the TGF-β signaling pathway. It has been reported that up to 62% of CRC patients with liver metastases have SMDA4 down-regulation[176], and the loss of SMAD4 has been shown to cause abnormal activation of STAT3 in pancreatic cancer cells, which then makes the expression of E-cadherin decrease, N-cadherin and vimentin increase, leading to the occurrence of EMT.[177] By constructing gene-virus CD55-Smad4 and overexpressing SMAD4, Xiao et al. confirmed that the metastasis and tumor cell stemness of CRC cells can be effectively inhibited by activating the Wnt /β-catenin signaling pathway.[178] In addition, there are a significant amount of studies confirming that some ncRNAs, affect the invasion and metastasis of CRC by regulating the TGF-β/Smad axis. For instance, LINC00941 promotes CRC metastasis via activation of the TGF-β/SMAD2/3 axis[179], and lncRNA ANRIL promotes CRC metastasis through activating the let-7a/TGF- β 1/Smad Axis.[180]

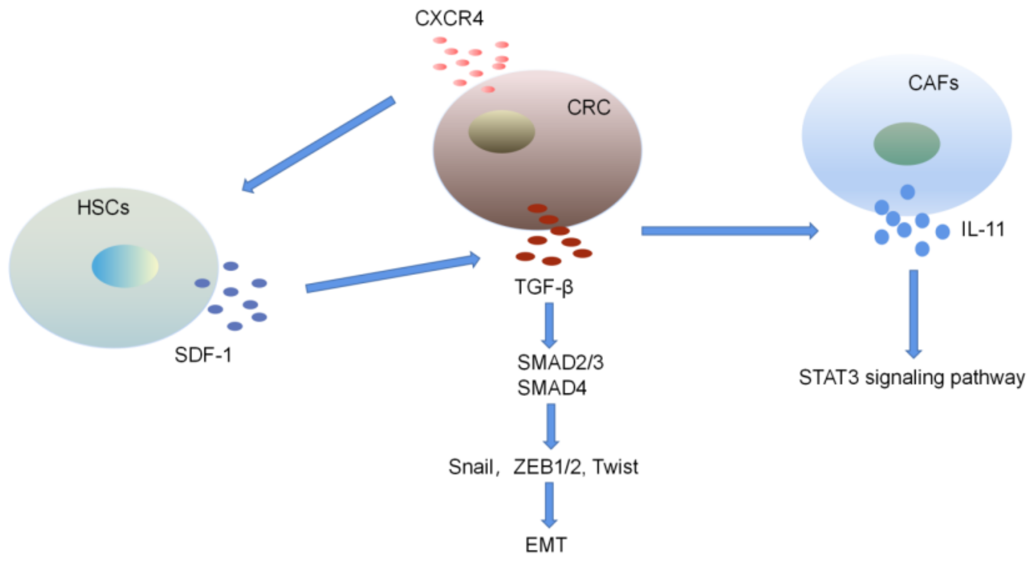

Fig 7. Schematic illustration of TGF- β *pathway regulatory mechanisms.*

CXCR4 secreted by CRC cells promoted sdF-1 secretion by HSCs. SDF-1 in turn acted on CRC and promoted its secretion of TGF- β . TGF- β can not only promote the secretion of IL-11 by CAFs to activate the STAT3 signaling pathway, but also further regulated gene transcription through SMAD in CRC cells and promoted the occurrence of EMT.

2.5.5 Other molecular targets and pathways involved in CRLM

L1CAM is a marker and mediator of metastasis initiating cells, which are required for orthotopic carcinoma propagation, liver metastatic colonization and chemoresistance.[181, 182] L1CAM is induced after loss of epithelial integrity, it then promotes tumor growth and metastasis.[183] The mechanism that epithelial cells enter into an L1CAM high phenotypic state is epithelial dissociation causes transcriptional downregulation of REST, in turn reducing REST occupancy of an L1CAM intronic enhancer and promoting L1CAM expression. Research and development of L1CAM inhibitory molecules-as a treatment of diffuse residual disease and metastatic disease is of great significance.[183] The scattered Lgr5 low and L1CAM high cells in the primary tumor can trigger the distant metastasis of CRC, but will dynamically emerge from the Lgr5 high primary tumor when the epithelial integrity is lost during the self-destructive tumor infiltration process, which is a distant metastatic organ

necessary for the survival and re-growth of diffuse cancer cells. Future studies may elucidate the close relationship between wound healing and metastasis.

Akt2 is one of the three subtypes in the Akt family, as it promotes cell movement/invasion.[184, 185] Akt2 is highly expressed in stage IV CRCs and liver metastases, meanwhile, loss of PTEN and overexpression of Akt2 synergistically promote metastasis.[186] The inducible loss of Akt2 in CRC cells strongly upregulates metastasis Suppressor 1 (MTSS1) at the messenger RNA and protein levels.[187] MTSS1 is a new gene regulated by Akt2, where the inhibition of MTSS1 is a key step for Akt2 to promote metastasis in CRC cells. Moreover, Elevated eukaryotic translation initiation factor 4A (EIF4A) in CRC patients was associated with poorer survival rate, poor response to oxaliplatin and more distant metastasis.[188] The EIF4A inhibitor silvestrol and oxaliplatin have a synergistic effect, and the combined use of the two may represent a new treatment strategy for patients with CRLM. The Trop-2 expression is another necessary for tumorigenesis and invasiveness of CRC cells.[189] Interestingly, ZFP57, an embryonic stem cell-specific transcription factor has been shown to promote liver metastasis of CRC.[190] Tang et al.[191] found phosphoprotein enriched in astrocytes-15 kDa (PEA15) was highly expressed in CRLM patients compared to non-metastatic. PEA15 promote CRC proliferation and the abilities of invasion and migration though activating the ERK/MAPK signaling pathway. PEA15 and ZFP57 may be a potential therapy biomarker for CRLM.

There are various epidermal growth factor-like domain protein 6 (MEGF6) in CRC, which induce EMT via transforming growth factor β (TGFβ)/SMAD signaling pathway to promote the transfer of CRC.[192] Through the analysis of expression profile microarray data, it is concluded that apolipoprotein E (ApoE) expression in normal mucosal tissues, primary CRC and CRLM increased sequentially.[193] The overexpression of ApoE is related to the progression of CRC, especially for stage II and simultaneous liver metastases, resulting in a poor prognosis for CRC patients. Moreover, lgG Fc binding protein (FCGBP) RNA and protein were significantly down-regulated in metastatic lesions, which were associated with the prognosis of CRLM.[194] In addition, two members of the S100 gene family, S100A6 and S100A4, are thought to be involved in the invasion and metastasis of cancer. When cancer cells form a glandular structure again in the center of metastatic nodules, the expression

level of S100A6 decreases.[195, 196] Knockdown of S100A4 restricts metastasis formation in a xenografted mouse model of CRC.[197] Moreover, S100A8, another member of the S100 family is correlated with TIMP-1-induced premetastatic niche in liver.[198]

A new member of the F-box protein family, FBX8, containing F-box and Sec 7 domain, can ubiquitinate and degrade HIF-1α such as CDK4 and C-Myc, downregulating their ability for promotion of angiogenesis, cycle progression and cell proliferation, respectively, thus regulating the CRC liver metastasis dormancy.[199] Moreover, Thrombospondin-1(THBS1) depletion inhibited migration and invasion of CRC cells through attenuating EMT.[200] Up-regulated THBS1 may be significantly correlated with CRLM, which requires further study.

Hypermethylated B4GALT1 was detected in the metastatic CRC case (mCRC) group and had adverse prognostic effects on CRC.[201] Glyco-genes *B4GALT1* may act as an additional novel biomarker for CRLM. Moreover, Ferritin Light Chain (FTL) as an oncogene leads to CRC cell resistance against 5-FU treatment and promoted CRC metastasis by competing with lncRNA Linc00467 for miR-133b binding site.[202] The expression of defensin β 4A (*DEFB4A*) was significantly up-regulated and experimentally proved that DEFB4A gene knockdown proved that DEF4BA promoted cell migration.[203] Sialyltransferase ST6GAL1 can increase the stability of ICAM-1 through sialylation, thereby inhibiting the transfer characteristics of CRC.[204]

Under hypoxic conditions, loss of the tumor suppressor p53 (encoded by TP53) provides cancer cells with a selective advantage, hypoxia which causes resistance to therapy and promotes metastasis in CRC.[205, 206] Recently, Li et al. reported the hypoxia inducible factor 1 alpha subunit (HIF1A) directly repressed the miRNA-34a in p53-defective CRC cells under hypoxia.[207] Conversely, p53 increases expression of miRNA-34a in CRC cells without hypoxia. Inhibition of Protein phosphatase 1 regulatory inhibitor subunit 11 (PPP1R11) by miRNA-34a prevented activation of STAT3 and inhibited the hypoxia-induced EMT and metastasis. Similarly, upregulated PPP1R11 was associated with TP53 mutations and metastasis to the liver. Targeting this pathway may represent a therapeutic

opportunity for managing metastatic disease. The NF-κB/p65 signaling pathway has a critical role in the mediation of GNA13 in CRC which was indicated by a decrease in GNA13-induced migration, invasion, and in addition, CXCL chemokine level increases after the inhibition of NF-κB/p65 signaling pathway with an inhibitor.[66] The novel quinazoline derivative MJ-56 interfered with the NF-κB signaling via impairing PI3K/AKT activation and subsequently reduced the NF-κB-mediated transcription of MMPs.[208]

Chu et al. [209] reported that mutant KRAS transcriptionally activates IGF-IR gene expression through Y-box-binding protein (YB)-1 upregulation via a novel MEK-Sp1-DNMT1-miR-137 pathway in CRC cells. Moreover, suppression of the expression of YB-1 and IGF-IR via inhibition of MEK hampers KRAS-driven colorectal liver metastasis in animal model studies. KRAS driven MEK-Sp1-DNMT1-miR-137-YB-1-IGF-IR signaling pathway, which might provide a mechanistic rationale for the use of a MEK inhibitor as an adjuvant, in combination with standard of care, to prevent the recurrence of colorectal liver metastasis in KRAS mutant CRC patients after receiving liver resection, however, further investigation is needed. Analysis of the TCGA database confirmed the up regulation of retinoblastoma binding protein 4 (RBBP4) in CRC tissues, and the over-expression of RBBP4 is related to nerve infiltration and poor chemotherapy effects.[210]

2.6 NcRNAs in CRLM

NcRNAs include long non-coding RNA (lncRNAs), microRNAs (miRNAs) and circular RNAs (circRNAs), which are emerging as the master regulators of cancer. The dysregulated expression NcRNAs have coding-independent functions in the progression of CRLM. In this section, we discuss some lncRNA and their role in CRLM(Table 2,3), circRNAs in CRLM(Table 4)， miRNAs in the regulation of cascade of CRLM (Table 5).

2.6.1 LncRNAs in CRLM

Recently, Zhang et al.[211] demonstrated that lncRNA LALC recruited DNA methyltransferases (DNMTs) to the LZTS1 promoter by coupling with EZH2 and then modified the expression of

LZTS1 through DNMTs-mediated DNA methylation in CRLM. Methyltransferase like 3 (METTL3) behaves as the 'writer' of m6A promoted m6A-methylation of oncogenic lncRNA XIST to inhibit CRC proliferation and invasion.[212] LncRNA LINC01578 activity was enhanced when nuclear factor kappa B (NF-κB) and Yin Yang 1 (YY1) were directly bound to the LINC01578 promoter.[213] Meanwhile, up-regulated LINC01578 interacted with and recruited EZH2 to NFKBIB promoter and further repressed NFKBIB expression, thereby activating NF-κB signaling. LINC01578 and NF-κB/YY1 formed a positive feedback loop, which promoted CRC metastasis. LncRNA HOTAIR suppressed HNF4α via recruiting SNAIL to promote the migration, invasion and EMT of CRC.[214] Intriguingly, a novel lncRNA, ENSG00000274093.1 binds to histone deacetylase 2 (HDAC2) and may act as a modular scaffold for the HDAC1/HDAC2 and EZH2 complexes, thereby altering EMT in CRC.[215] LncRNA TPT1-AS1 induces angiogenesis and metastasis in CRC via theTPT1-AS1/NF90/VEGFA axis.[216] Additional research have shown that the expression level of LncRNA clmat3 and SNHG15 in CRC with liver metastasis was significantly higher than in those CRC without liver metastasis.[217, 218] Conversely, some lncRNAs play a role of tumor inhibition, for example, low expressed lncRNA SATB2-AS1 inhibited cell metastasis and regulated the immune response of CRC by *cis*-activating SATB2.[219] Moreover, lncRNA MIR22HG， as a tumor suppressor in CRC, competitively interacted with SMAD2 and modulated the activity of TGFβ pathway, thereby inhibited cell survival and tumor metastasis in vitro and in vivo.[220]

LncRNA UICLM acted as a ceRNA for miR-215 to regulate ZEB2 expression, then induced CRC liver metastasis , which may offer a novel prognostic marker and therapeutic target for this disease.[221] LncRNA GAPLINC, HOTAIR, RP11-757G1.5 , LINC00460, MIR4435-2HG, PlncRNA-1, DANCR and TP73-AS1 were proven to be positively associated with the proliferation and liver metastasis of CRC through the GAPLINC/miR-34a/c-MET axis, HOTAIR/miR-214/ST6GAL1 axis, RP11-757G1.5/miR-139-5p/YAP1 axis, LINC00460 /miR-613/sphingosine kinase 1 (SphK1) axis and MIR4435-2HG/miR-206/YAP1 axis, PlncRNA-1/miR-204/ Wnt/β-catenin regulatory network, DANCR/miR-577/HSP27 signaling axis and TP73-AS1/miR-194/TGFαsignaling axis, respectively.[222-229] Furthermore, lncRNA ZEB1-AS1 acted as ceRNA to upregulate p21-activated kinases 2 (PAK2) by sponging miR-455-3p, thus facilitating colon adenocarcinoma cell growth and

metastasis.[230] Zhou et al. [231] reported that SRY-box transcription factor 9 (SOX9) activated the transcription of lncRNA FARSA-AS1. FARSA-AS1 upregulated SOX9 and FARSA via binding to miR-18b-5p directly. Overall, SOX9-FARSA-AS1-SOX9/FARSA loop participated in cell growth, stemness, and metastasis in CRC. In addition, lncRNA HSD17B11-1:1 acted as a sponge for miR-338-3p to upregulate the expression of MACC1 to promote CRC cell proliferation, mobility and invasion *in vitro* and *in vivo*.[232] B3GALT5-AS1, is an antisense lncRNA, located in Chr21q22.2, which was first reported in Wang et al study.[233] They found B3GALT5-AS1 was decreased in CRC contrast to normal colonic epithelium. B3GALT5-AS1 directly bound to the promoter of miR-203, repressed miR-203 expression, upregulated miR-203 targets ZEB2 and SNAI2, and induced EMT, suggesting that down-regulated B3GALT5-AS1 is a biomarker of CRC metastasis and, more importantly, activating B3GALT5-AS1/miR-203/EMT axis may be a potential therapeutic strategy for CRLM.[233] Recently, B3GALT5-AS1 has been reported to be related to tumor node metastasis (TNM) stage and histological differentiation.[234] There are also other lncRNA that regulated CRLM through ceRNA mode, for example MALAT1, SNHG14, SNHG7, H19, and LINC00485. Taken together, these lncRNA could be a therapeutic target and promising biomarkers for prognosis prediction of CRLM.

Table 2 LncRNA in CRLM

LncRNA	Role	Function	Refs.
CLMAT3	Promote	metastasis	217
SNHG15	Promote	metastasis	218
TPT1-AS1	Promote	angiogenesis， metastasis	216
ENSG00000274093.1	Promote	EMT	215
HOTAIR	Promote	migration, invasion，EMT	214
LALC	Promote	metastasis	211

LOC441461	Promote	cell growth and motility	235
SATB2-AS1	Suppress	cell growth and metastasis	219
MIR22HG	Suppress	Metastasis, EMT	220

Table 3 LncRNA/miRNA/mRNA ceRNA network in CRLM.

LncRNA	Role	Function	Shared miRNA	Competitor mRNA	Refs.
H19	Promote	EMT progression	miR-138, miR-200a	Vimentin, ZEB1, ZEB2	236
TP73-AS1	Promote	proliferation, migration, and invasion	miR-194	TGFα	227
MIR4435-2HG	Promote	proliferation, metastasis.	miR-28-5p	YAP1	224
UICLM	Promote	metastasis	miR-215	ZEB2	221
RP11-757G1.5	Promote	proliferation, metastasis	miR-139-5p	YAP1	217
ZEB1-AS1	Promote	cell growth and metastasis	miR-455-3p	PAK2	230
FARSA-AS1	Promote	stemness, metastasis	miR-18b-5p，miR-28-5p	SOX9，FARSA	231

HSD17B11-1:1	Promote	proliferation, mobility, and invasion	miR-338-3p	MACC1	232
SNHG7	Promote	EMT，metastasis	miR-216b	GALNT1	237
SNHG14	Promote	metastasis	miR-186-5p	EZH2	238
DANCR	Promote	proliferation，metastasis	miR-577	HSP27	226
MIR4435-2HG	Promote	proliferation，metastasis	miR-206	YAP1	224
Plncrna-1	Promote	proliferation，metastasis	miR-204	Wnt/p-catenin	225
MALAT1	Promote	metastasis	miR-106b-5p	SLAIN2	239
HOTAIR	Promote	metastasis	miR-214	ST6GAL1	223
GAPLINC	Promote	migration，invasion	miR-34a	c-MET	229
B3GALT5-AS1	Promote	EMT，metastasis	miR-203	ZEB2，SNAI2,	233
LINC00485	Suppress	cell growth，metastasis	miR-581	EDEM1	240

2.6.2 CircRNAs in CRLM

NSUN2 is a circRNA recently identified upregulated in CRLM and N6-methyladenosine modification of circRNA among which circRNA circPPP1R12A activated the Hippo-YAP signaling pathway to promote the growth and metastasis of CRC[241]. NSUN2 was more likely to enter the cytoplasm, then in the cytoplasm increased the stability of HMGA2 mRNA to induce CRC metastasis

progression. [242] Equally noteworthy, circRNA circ_0124554 inhibited the ubiquitination of AKT to promote the early metastasis particularly for the lymph node-negative colorectal cancer patients with synchronous liver metastasis.[243]

CircRNAs serve as a sponge to protect multiple oncogenes from being attacked by miRNAs and among these, global exaltation of circ_0115744 circRNAs circAPLP2, hsa_circ_000984, hsa_circ_0071589 and hsa_circRNA_102209 acted as ceRNA to enhance tumor metastasis via circ_0115744/miR-144/EZH2 axis, circAPLP2 /miR-101-3p /Notch1 axis, hsa_circ_000984/miR-106b/CDK6 axis, hsa_circ_0071589/miR-600/EZH2 axis and hsa_circRNA_102209/miR-761/RIN1 axis, respectively.[244-246] Similarly, circRNA NSD2 promoted metastasis of CRC by sponging miR-199b-5p, which could increase the expression of DDR1 and activate JAG1 signaling.[247] More interestingly, one circRNA contains various binding sites for different miRNAs reveals the complex role of circRNA in cancer malignancy. CircRNA CCDC66 functions as miRNA sponge to reduce the destruction of MYC mRNA by miRNA-33b and miR-93.[248] In addition, Zhi et al.[249] also identified a novel and conserved circRNA hsa_circ_102049 as a promoter of CRC metastasis. Mechanistically, hsa_circ_102049 acted as a sponge of the tumor suppressor miR-761 and miR-192-3p and modified the subcellular localization of the RNA binding protein DGCR8, then regulated the levels of the FRAS1, which synergistically enhanced the adhesion, migration, and invasion abilities of CRC cells. Interestingly, hsa_circ_0001178 sponging miR-382, miR-587 and miR-616 to upregulate ZEB1, which in turn increase hsa_circ_0001178 expression via physically binding to hsa_circ_0001178 promoter region, thereby based on this positive feedback ceRNA axis, consequently facilitated the invasion and metastasis of CRC .[250]

circRNAs also play a inhibition role of CRLM, for example, circRNA Hsa_circ_0009361 acted as an inhibitor for miR-582 and suppressed the CRC cell metastasis.[251] Similarly, circRNA-FNDC3B negatively and directly regulated miR-937-5p to promote the expression level of the tumor-suppressor TIMP3, thereby blocking the metastasis, invasion and angiogenesis of CRC.[252] In addition, circITGA7 inhibited the Ras signaling pathway and promoted the transcription of ITGA7, thereby suppressed the proliferation and metastasis of CRC cells .[253]

In the latest studies, circRNA_0001178, circRNA_0000826 and circRNA hsa_circ_0000826 induced by the hypoxia in CRC were dramatically upregulated in CRLM tissues, which can be a potential biomarker of CRC liver metastasis.[254, 255] Validation studies of specific molecular mechanisms are needed to identify optimal miRNAs and marker panels that can be used in clinical care.

Table 4 CircRNA associated with the development of CRLM

CircRNA	Role	Function	Refs.
Has_circ_0071589	Promote	cell growth, invasion, and migration.	245
hsa_circ_0001178	Promote	invasion， metastasis	250
circ_0124554	Promote	metastasis	243
hsa_circ_102049	Promote	migration，invasion，metastasis	249
Hsa_circ_000984	Promote	metastasis	244
hsa_circRNA_102209	Promote	cell growth ， metastasis	256
CircRNA_0001178	Promote	metastasis	250
CircRNA_0000826	Promote	metastasis	255
Circ-NSD2	Promote	metastasis	247
circAPLP2	Promote	proliferation， metastasis	246
CircPPP1R12A	Promote	proliferation，migration and invasion	241
CircCCDC66	Promote	cell growth ， metastasis	248
CircRNA NSUN2	Promote	metastasis	242

circ_0115744	Promote	metastasis	257
CircITGA7	Suppress	proliferation，metastasis	253
CircRNA-FNDC3B	Suppress	metastasis, invasion, and angiogenesis	252
Hsa_circ_0009361	Suppress	cell growth ，metastasis	251

2.6.3 MiRNAs in CRLM

Recent studies have shown that miRNAs were involved in the context of EMT in CRC. It has been reported that miR-200 family (miR-200a, miR-200b, miR-200c, miR-141, and miR-429) are recognized as regulators of the epithelial phenotype through repression of ZEB1 and ZEB2 mRNA translation [258-260] The miR-200c levels in primary CRC without liver metastasis is lower than the metastasis with primary tumor tissues, which highlight a crucial role for miR-200c in CRC metastasis.[259] The overexpression of miR-429 could play an oncogenic role in the cellular processes of CRC by targeting SOX2 [261]

Additionally，miR-181a and miR-30b were highly expressed in CRLM by promoting EMT through inhibiting Wnt inhibitory factor-1(WIF-1) and inhibiting the SIX1 gene, respectively.[262, 263] Moreover, miR-30a is an important regulator of transmembrane-4-L-six-family protein (TM4SF1), VEGF, and E-cadherin for CRC cell motility and EMT.[264] Manganese superoxide dismutase (MnSOD) was required for down-regulation of epithelial markers and up-regulation of mesenchymal markers in CRC cells, indicating that it promoted the EMT, which was reduced by overexpression of miR-212.[265] Methylated miR-34c-5p significantly suppressed the metastasis of CRC cells via directly modulating the SATB2.[266] Recently, the cytokine IL-6 was found to activate the oncogenic STAT3 transcription factor, which directly repressed the MIR34A gene, while miR-34a directly regulated IL-6R. As a result, the IL-6R/STAT3/miR-34a loop promoted CRC invasion and metastasis.[56] MiR-186-5p affected metastasis and EMT process of CRC cell by inhibition of ZEB1, while miR-17-5p regulated EMT by targeting vimentin.[267, 268]

Furthermore miR-34a/SNAIL loop and miR-200/ZEB1/2 loops, miR-15a/16-1/AP4 feedback loop is also found in primary CRC. The tumor-suppressive miR-15a and miR-16-1, which targeted AP4 3'-UTR, and inhibited CRC cell migration and invasion.[269, 270] Overexpression of miRNA let-7 promoted EMT via targeting HMGA2[271], while Lin28 could inhibit let-7 in conjunction with OCT4, SOX2, and KLF4.[272] MiR-10a suppressed CRC metastasis by regulating the epithelial-to-mesenchymal transition via targeting matrix metalloproteinase 14 (MMP14) and actin gamma 1 (ACTG1).[273] Unlike miR-10a, up-regulated miR-10b in metastatic CRC tissues and cell lines inhibited E-cadherin expression and enhanced cyclin D1, which were partly abrogated after targeting KLF4.[274]

The expression of miR-21 was higher in tumor tissue than in adjacent normal tissue of 156 CRC patients by TaqMan MicroRNA assays.[275] It is reported that transglutaminase 2 (TG2) expression was observed in CRC primary tumors but lost in liver metastases and TG2 inhibited by miR-19 could affect the invasive ability of CRC cells.[276] Moreover, Zhang et al.[147] demonstrated that the overexpression of PRL-3 in CRC cells induced the expression of miR-21, miR-17 and miR-19a by activating signal transducer and STAT3. A positive correlation was observed between PRL-3 and these miRNAs in matched primary colon cancer tissues and metastatic lesions.[147] MiR-21 could significantly reduce Pdcd4-protein amounts and increased invasion.[277] Additionally, Feiersinger et al.[278] have identified that the expression of miR -21 was significantly lower in liver metastases as compared to the primary CRC. The overexpression of miR-200 and miR-141 inhibited apoptosis and induced migration.[279] These indicated that miR-21 might be involved in the initiation and miR-200 and miR-141 exacerbated liver metastasis of CRC.

Interestingly, highly expressed miRNA-20a-5p and miR-298 were positively correlated with CRLM by suppressing drosophila mothers against decapentaplegic protein4(smad4) expression and targeting PTEN, respectively.[280, 281] Inhibition of miR-15b significantly decreased colony formation ability, invasion, and migration of HCT116 cells in vitro and liver metastasis of HCT116 tumors in vivo via increasing metastasis suppressor-1 (MTSS1) and Klotho protein expression.[282 283] Up-regulation of

miR-885-5p had strong tumor-promoting effects through by targeting cytoplasmic polyadenylation element binding protein 2 (cpeb2), von Willebrand factor (vWF) and insulin-like growth factor binding protein 5 (IGFBP5), and its potential role in promoting cell migration, invasion and liver metastasis.[284, 285] The expression of miR-224 increased consistently with tumor burden and the steady state of microsatellites, and can enhance CRC metastasis in vitro and in vivo.[286]

For long, it was known that the patients with CRC have demonstrated a significant evaluated expression of serum miR-497.[287] In the mouse model, Qiu et al.[288] found that a combination of miR-497 and bufalin had a synergistic effect on the inhibition of CRC metastasis. They also found that miR-497 targeted the expression of vascular endothelial growth factor-A (VEGF-A).[289] Notably, miR-497 exerted its oncogenic function by targeting fos-related antigen-1 (Fra-1). [290] Recently, AGAP2-AS1 regulated fibroblast growth factor receptor 1 (FGFR1) expression by sponging miR-497 in the migration and invasion of CRC cells.[291]

However, liver metastasis cells of CRC also downregulate the expression of miR-133a, miR-17-5p, miR-99b-5p, miR-214, miR-26, miR-30e-5p and miR-328-3p that suppress cell migration and invasion. MiR-133a, as a tumor suppressor, inhibited cell proliferation, invasion, and migration by targeting oncogenic EIF4A1.[292] MiR-99b-5p was differently expressed in primary CRC and liver metastasis and functioned as a tumor-suppressive miRNA to affect cell migration by targeting mTOR in metastatic CRC.[293] In an orthotopic mouse model of CRC, Ding et al.[294] found that APOBEC3G enhanced CRC cell migration and invasion via inhibition of miR-29-mediated suppression of MMP2. Downregulation of miR-214 promoted proliferation, migration, and invasion in CRC cell lines via increasing level of fibroblast growth factor receptor 1(FGFR1), which can lead to the occurrence of liver metastasis.[295, 296] Li et al.[297] reported that tumor-suppressive miR-26a and miR-26b inhibited the target gene fucosyltransferase4 (FUT4) expression, resulting in migratory behavior of CRC. Laudato et al.[298] found that miR-30e-5p was a novel effector of P53-induced suppression of migration, invasion, and metastasis by directly targeting both integrin alpha-6 (ITGA6) and integrin beta-1 (ITGB1). In addition, miR-30b-5p functioned as a metastasis suppressor by targeting Rap1b, a Ras family small GTPase that regulates cell adhesion and mobility.[299]

A recent study has shown that low miR-196b-5p expression is significantly associated with metastases and poor outcomes, further proving that miR-196b-5p inhibition led to significantly increased CRC cell migration/invasion and metastases formation in mice via the interaction with HOXB7 and GALNT5.[300] A contemporary study by Luo et al. has demonstrated that miR-432-5p functions as a tumor suppressor to inhibit cell migration and invasion by negatively regulating CXCL5 expression.[301] Interestingly, miR-328-3p may inhibit proliferation and metastasis of CRC cells via suppressing Girdin expression and associated PI3K/Akt signaling pathway[302] MiR-365a-3p to inhibit the metastasis of CRC cells by negatively regulating ADAM10 and inactivating the JAK/STAT signaling pathway.[303] Moreover, miR-487b directly targeted LRP6, a receptor for WNT/β-catenin signaling to inhibit liver metastasis.[304]MiR-143-3p, miR-132 and miR-146a significantly abolished the development of liver metastases by directly targeting ITGA6/ASAP3, anoctamin 1 (ANO1) and c-Met, respectively.[305-307] In addition to control the intravasation, miRNAs also targeted genes; for example, miR-21 regulated Pdcd4 which induced intravasation and metastasis.[277] A positive significant correlation between expression of miR-126 and epidermal growth factor–like domain 7 (EGFL7) were seen in liver metastases, which supported miR-126 may act as a regulator in angiogenesis and intravasation process.[308] Further experiments are needed to explore more detailed forms of connection between miR-126 and EGFL7. Low expression of miR-26b was significantly associated with the invasiveness and metastasis of CRC cells. Hansen et al.[309] reported that miR-31-mediated repression of E-selectin impaired the metastatic potential of CRC cells.

One study showed that miR-103/107 potentiated the colonization of CRC cells at a metastatic site by targeting the known metastasis suppressors death-associated protein kinase (DAPK) and Krüppel-like factor 4 (KLF4) in CRC cells.[310] Similarly, in mice models of CRC, the overexpression of miR-103/107 enhanced local invasion and liver metastasis effects. Another report revealed that miR-483 and miR-551a inhibited liver colonization and metastasis.[311] Metastatic foci in new organs promote angiogenesis due to increased demand for oxygen and nutrients. Therefore, angiogenesis has become a necessary condition for the survival of metastatic foci.

Table 5 MiRNAs in CRLM

MiRNA	Role	Function	Molecular target	Refs.
MiR-15b	Promote	invasion, metastasis	MTSS1, Klotho	[282]
MiR-30b	Promote	EMT, metastasis	SIX1	[263]
MiR-19	Promote	invasion, metastasis	TG2,STAT3	[147, 276]
MiR-17	Promote	metastasis	STAT3	[147]
MiR-21	Promote	invasion, intravasation, metastasis	STAT3, Pdcd4	[147]
MiR-103/107	Promote	colonization,metastasis	DAPK,KLF4	[310]
MiR-181a	Promote	EMT	WIF-1	[262]
MiR-885-5p	Promote	migration,invasion	Cpeb 2	[285]
MiR-20a-5p	Promote	invasion,metastasis	Smade	[280]
MiR-429	Promote	apoptosis	SOX2	[261]
MiR-298	Promote	invasion	PTEN	[281]
Let-7	Promote	EMT, metastasis	HMGA2	[271]
MiR-21	Promote	migration,invasion	Pdcd4	[277]
MiR-497	Promote	metastasis	Fra-1, VEGF-A	[289]
MiR-10b	Promote	EMT, metastasis	KLF4	[274]

MiR-10a	Suppress	EMT, metastasis	MMPI4 , ACTG1	273
MiR-200	Suppress	EMT	ZEB1/2,ETS1,FLT1	260
MiR-212	Suppress	EMT, metastasis	MnSOD	265
MiR-30a	Suppress	EMT	TM4SF1，VEGF，E-cadherin	264
MiR-31	Suppress	metastasis	E-selectin	309
MiR-26a	Suppress	metastasis	FUT4	297
MiR-26b	Suppress	invasion, metastasis	FUT4	297
MiR-551a	Suppress	colonization, metastasis	-	311
MiR-483	Suppress	colonization, metastasis	-	311
MiR-195	Suppress	angiogenesis	VEGF	312
MiR-99b-5p	Suppress	migration	mTOR	293
MiR-29	Suppress	migration,invasion	MMP2	313
MiR-214	Suppress	migration,invasion	FGFR1	295
MiR-30e-5p	Suppress	proliferation, metastasis	ITGA6， ITGB1	298
MiR-196b-5p	Suppress	migration, invasion	HOXB7 , GALNT5	300
MiR-34a	Suppress	migration， invasion ， EMT	IL-6R	56

MiR-365a-3p	Suppress	migration,invasion , EMT	ADAM10	303
MiR-487b	Suppress	metastasis	LRP6	304
MiR-186-5p	Suppress	EMT	ZEBl	267
MiR-15a/16-1	Suppress	EMT	AP4	270
MiR-132	Suppress	invasion	ANO1	306
MiR-17-5p	Suppress	EMT	vimentin	268
MiR-146a	Suppress	metastasis	c-Met	307
MiR-143-3p	Suppress	metastasis	ITGA6 and ASAP3	305
MiR-328-3p	Suppress	metastasis	Girdin	302

2.7 Cancer Stem Cells (CSCs) in the Progression to CRLM

CSCs as seed cells for tumorigenesis can initiate and sustain the growth of tumors. CSCs can be identified by specific markers, including CD133, LGR5,CD44, ALDH1, CD24, CD166, CD29, CD26, and CD51.[314, 315] CRC-CSCs can increase the ability of distant metastasis and colonization.[316, 317] It is thus possible that targeting CSCs may have widespread clinical implications.[318] Among the epithelial cells that form the surface of the intestine, crypt-based columnar cells (Lgr5+ cells) expressing the receptor protein Lgr5 function as stem cells during the maintenance of intestinal homeostasis, and they are also the starting cells for CRC.[319] The intestinal epithelium has significant self-renewal ability, and the rapidly proliferating Lgr5+ cells are usually responsible for the daily production of all types of intestinal epithelial cells.[319] Lgr5− cells can drive wound healing by first transdifferentiating into a stem-like Lgr5+ state.[320] The selective elimination of LGR5+ cells leads to

temporary tumor regression, and other cells exhibit compensatory proliferation, then the reappearance of LGR5+ CSCs drives tumor regeneration.[321] It has been demonstrated that selective Lgr5+ cell ablation restricts primary CRC growth, but does not result in tumor regression, because proliferative Lgr5− cells could continuously attempt to replenish the Lgr5+ CSCs pool, leading to rapid re-initiation of tumor growth upon treatment cessation.[322] Moreover, Fumagalli et al. provided direct evidence most disseminated CRC cells in circulation were Lgr5- and formed liver metastases in which Lgr5+ CSCs appeared.[323] Cell state plasticity is very important to promote CRC metastasis. The signaling pathways responsible for the reproduction of Lgr5+ cells may yield exciting new strategies for treating CRLM. Wang et al.[324] reported prostaglandin E2 (PGE2) induced CSCs and enhanced liver metastasis by activating NF-κB via EP4-MAPK and EP4-PI3K-Akt pathways.

DNA methylation is considered to be a potential epigenetic mechanism to maintain CSCs, and the loss of DNMT can reduce the occurrence of tumors by limiting the CSC pool.[325] Li et al.[326] found that the DNMT inhibitor 5-Aza-2'-deoxycytidine (5-AzaDC) significantly reduced the abundance of colorectal CSC in vitro and inhibited the growth of liver metastatic tumors in vivo. They also found that 5-AzaDC inhibits the expression of active β-catenin and down-regulates the Wnt signaling pathway. A new study points out the mechanism exerted by methylation on endothelin system genes expression can be compromised in CRLM.[327]Notably, CSCs are critical for the formation and maintenance of liver metastasis. Together, our data highlights distinct CSC dependencies for primary versus metastatic tumor growth and suggest that targeting CSCs may be one direction of treatment for CRLM.

2.8 Role of Circulating Tumor Cells (CTCs) in CRLM

Patient-derived CTCs has been shown to bear all the functional attributes of CSCs.[328] The markers expressed by CTCs are similar to the cancer niche, which are conducive to liver metastasis.[329, 330] CD133+ CD44+ CD54+ cellular subpopulation of CTCs has a prognostic value in CRLM, especially in the survival of CRLM who did not receive surgical treatment for metastasis.[331] Patient-derived CTCs lines are tumorigenic in subcutaneous xenografts and are also able to colonize the liver after intrasplenic injection.[328] Drug test by in vitro culture of CTCs may facilitate access to personalized

medicine. TAMs regulated JAK2/STAT3 signaling pathway by secreting IL6, thereby inhibiting miR-506-3p expression and promoting FoxQ1 expression. CTC cells then produced CCL2 to recruit more TAMs. TAMs and CTC both interacted to promote the occurrence of metastasis.[332] These findings suggest targeting strategies against CTC clusters may be effective in the treatment of liver metastases.

2.9 Metabolic factors in CRLM

Tumors are also a metabolic disease, and metabolic changes are closely related to every process of tumor metastasis. The process of transfer requires a large amount of energy supply, and some enzymes and molecules that affect energy metabolism also act as gas stations in the process of transfer. In the extracellular space, creatine kinase brain (CKB) used ATP-catalyzed phosphorylation of the metabolite creatine to form phosphocreatine, which can produce large amounts of ATP into CRC cells as an energy reserve to maintain the energy requirements of CRC cells during anoxia during metastasis.[333] CKB promoted the development of liver metastasis, and targeted inhibition of its activity was also the direction of future treatment of CRLM. In addition to phosphocreatine generating energy for cancer cells, the fatty acid oxidation (FAO) pathway is also an important energy source within cancer cells. In detached CRC cells, under the action of carnitine palmitoyl transferase 1 (CPT1), FAO was greatly activated, thus increasing the ability of cells to metastasize.[334] CRC cells implanted in the liver promoted the metabolism of fructose by upregulating enzyme aldolase B (ALDOB), which provided energy for cell growth in liver metastases.[335] This particular metabolic pathway change was found only in liver metastases and not in other sites of metastasis or primary tumors. All in all, the metabolic pathways of tumors adapt to the environment during metastasis, and there are still many problems to be studied.

3.Diagnosis

CRLM should be identified in patients with a confirmed diagnosis of colorectal cancer. Among them, approximately 10%-15% of patients have synchronous liver metastases——the liver metastasis is

diagnosed at the same time with CRC.[10] The main content of our diagnostic section is centered on synchronous liver metastases, and the assessing approaches of metachronous liver metastases are similar to the former and executed during the follow-up period.

Biomarker testing should be performed routinely for diagnosis of the synchronous liver metastasis. Serum cancer embryo antigen(CEA) could be the routine choice for screening, and carbohydrate antigen 19-9(CA19-9) might be a supplementary indicator without an evaluated CEA value.[336] Detection of CEA and CA19-9 should also be executed at intervals after resection. RAS is also a biomarker predicting the efficacy of anti-epidermal growth factor receptor (EGFR) therapy, which determines the choice of treatment strategy. Thus, RAS testing should be executed in all patients with colorectal cancer, and include NRAS exons 2, 3, 4 and KRAS exons 2,3,4 at least.[337]BRAF testing also could help to select the therapy option and predict the prognosis. In addition to serum tumor marker examination and pathological staging evaluation, imaging examinations such as liver ultrasound and abdominal contrast-enhanced CT should be routinely performed to screen and diagnose liver metastases.[338]

Magnetic resonance imaging (MRI) with liver specific contrast should be used prior to surgery when the liver metastases are resectable. Moreover, MMR / microsatellite instability (MSI) testing, UGT1A1 detection of UGT1A1 protein, human epidermal growth factor receptor 2 (HER2) detection are recommended to provide the basis for clinical decisions on post line treatment in advanced patients. The PET-CT examination is not used as a regular recommendation and may be applied as appropriate when the condition is required. Needle biopsies of liver metastases are rarely required.[339]

After radical colorectal cancer, patients should follow up regularly to understand whether the occurrence of metachronous liver metastases. During colorectal cancer surgery, routine exploration of the liver must be performed to further exclude the possibility of liver metastases. Suspicious liver nodules found during the operation can also be biopsied.[339] Tumor markers such as serum CEA,CA19-9 and, liver ultrasound and chest / abdominal / pelvic enhancement CT scan should be routinely examined to conduct screenings and diagnose liver metastases. Patients with high suspicion in ultrasound or CT imaging should not be diagnosed with liver MRI, and recommended consistency

of imaging methods during follow-up. PET-CT scan is not normally recommended. Electronic colonoscopy should be performed within 1 year after surgery.[340]

4.Treatment

4.1 Surgery

Surgery has become the standard of care for patients with CRLM. When CRLM may be limited to a few liver metastatic foci, surgical resection is the first choice. Patients with liver metastases that cannot be resected initially should also receive surgical treatment when they are transformed into resectable lesions after treatment.[341]For these patients who are suitable for complete surgical resection, they are often accompanied by lymph node infiltration and dissemination of occult micro metastases. Therefore, it is necessary to cooperate with adjuvant radiotherapy and chemotherapy and targeted therapy.

4.2 Radiation therapy

In patients with CRLM, radiation therapy of liver metastasis remains controversial, due to the tolerated dose of whole liver radiation being much lower than the lethal dose required by tumor cells. The use of image guidance technology can make radiotherapy more precise. For patients with liver metastases from CRC with normal liver function, conventional radiotherapy techniques can be used to treat liver metastases.[342, 343]

4.3 Ablation therapy

In cases where liver metastases cannot be surgically removed, appropriate ablation treatments, such as radiofrequency ablation, microwave ablation, and cryotherapy, should be selected on the basis of systemic chemotherapy based on their location, treatment goals, treatment-related complications, and the patient's own conditions to strengthen control of local lesions.[344] However, care should be taken to avoid extrahepatic heat damage and incomplete ablation when performing ablation treatment.[341]

4.4 Systemic Therapy

Patients with CRLM usually receive chemotherapy after surgical resection. It recommends FOLFOX (5-fluorouracil [5-FU] + oxaliplatin + leucovorin [LV]), FOLFIRI (5-FU + irinotecan + LV), XELOX (capecitabine + oxaliplatin), infusional 5-FU/LV or capecitabine, or FOLFOXIRI (5-FU + oxaliplatin + irinotecan + LV) for patients with mCRC who are suitable for intensive therapy according to the current National Comprehensive Cancer Network (NCCN) guidelines.[345] Research data in recent decades has shown that patients with metastatic CRC receive systematic chemotherapy, and their OS duration is extended to nearly 20 months.[345, 346] Currently, patients with initially unresectable CRLM has received hepatic arterial infusion chemotherapy in combination with systemic chemotherapy, which is not widely used.[347]

4.5 Anti-angiogenesis therapy

Tumor increases energy supply through angiogenesis during metastasis, and anti-angiogenesis therapy has become an important therapeutic strategy for CRC. It is of great significance to explore the angiogenesis mechanism of CRLM. The targeted agents for anti-angiogenesis therapy under clinical trials in CRLM are summarized in tables 6. Bevacizumab and Cetuximab are molecularly targeted drugs that have been developed specifically for endothelial growth factor receptor (EGFR). Clinical trials have proven that anti-EGFR monoclonal antibodies panitumumab and cetuximab can effectively inhibit metastatic CRC.[348] Saltz et al.[349] evaluated the efficacy of adding bevacizumab to XELOX or FOLFOX-4 in 1401 CRLM patients. The median duration of PFS was 9.4 months in the bevacizumab group and 8.0 months in the placebo group (P = 0.0023). Moreover, cetuximab added to FOLFOX-4 compared with FOLFOX-4 alone, cetuximab was associated with a clinically significant increase in overall response (61% vs. 37%; P compared with FOLFOX-4 alone, especially In KRAS wild-type tumors, it can reach a level of 0.011), which reduces the risk of disease progression (hazard ratio = 0.57; P = 0.0163). [350] Patients with RAS wt mCRC do not respond to anti-EGFR antibodies, probably due to ineffective inhibition of oncogenic RAS signaling.[351] A recent randomized study conducted by Qin et al.[352] confirmed cetuximab in combination with FOLFOX as an effective standard-of-care first-line treatment regimen for patients with RAS wt mCRC. However, the NORDIC-VII multicenter phase III trial suggested that cetuximab did not add significant benefit

to the fluorouracil, leucovorin, and oxaliplatin (Nordic FLOX) regimen in first-line treatment of mCRC.[353]

It was confirmed by clinical trials that dual chemotherapy (fluoropyrimidine plus oxaliplatin or irinotecan) with targeted drugs that add anti-EGFR antibodies can further increase the response rate to about 60%.[349, 354, 355] Bevacizumab plus XELOX (CapeOX) chemotherapy has no negative impact on intrahepatic immune cells in resectable CRLM patients.[356] Although the survival rate is improved by FOLFOX treatment, chemotherapy still has many disadvantages, such as the destruction of the human immune system, loss of appetite, weight loss, hair loss and so on after chemotherapy. In a recent study, nano codelivery of oxaliplatin and folinic acid (Nano-Folox / 5-Fu) with 5-fluorouracil significantly promoted the blood circulation and tumor accumulation of drugs in the orthotopic CRC mouse model.[357] Nano-Folox / 5-Fu with 5-fluorouracil can not only provide anti-cancer cell toxicity, but also induce immunogenic cell death. In addition, the combination of anti-PDL1 monoclonal antibody with Nano-Folox / 5-FU significantly reduced liver metastasis in mice. These results suggest that the combination strategy based on Nano Folox has some therapeutic significance in CRLM. Moreover, gene set enrichment analysis discovered that the mTOR pathway was activated in patients undergoing oxaliplatin based therapy, which suggested it could be used in combination with cytotoxic chemotherapy and targeted drugs, and may have good therapeutic effects.[358] For patients with initially resectable CRLM, chemotherapy can improve progression-free survival (PFS) but cannot improve overall survival (OS) [359], and further addition of EGFR-targeted antibodies is not beneficial.[360] However, in patients with unresectable CRLM, the use of bevacizumab may further increase the proportion of patients eligible for surgical resection.[361] For patients whose CRLM is initially unsatisfactory or unresectable, targeted anti-angiogenesis drugs plus chemotherapy can also produce a higher remission rate and improve resectable.[362]

TSU68[(Z)-5-[(1,2-dihydro-2-oxo-3H-indol-3-ylidene)methyl]-2,4-dimethyl-1H-pyrrole-3-propanoic acid; SU6668] is a potent antiangiogenic agent. In some preclinical models， TSU68 has previously been shown to have a powerful effect in preventing liver metastasis of CRC.[363, 364] Inhibiting CXCL1 expression in the premetastatic liver was the detailed mechanisms by which

TSU68 suppresses tumor metastases.[67] Anti-angiogenic agents may modulate the premetastatic niche in target organs.

Table 6 Targeted agents for anti-angiogenesis therapy under clinical trials

NCT numbers	Phase	Therapeutic agents	Condition	Result	
NCT01001377	Phase 3 (N=1010)	Panitumumab vs Cetuximab	mCRC (KRAS-WT)	Median OS	10.4 m VS 10 m
NCT01228734	Phase 3 (N=393)	Cetuximab +FOLFOX-4 vs FOLFOX-4	mCRC (RAS-WT) First line	PFS<br>Median OS	9.2m vs 7.4 m<br>20.7m vs 17.8 m
NCT00265850	Phase 3 (2334)	Cetuximab +mFOLFOX6 vs bevacizumab +mFOLFOX6	mCRC (KRAS-WT)	PFS<br>Median OS	10.5m vs 10.5m<br>30m vs 29m
NCT00006479	Phase 3 (364)	Perioperative chemotherapy with FOLFOX4 vs surgery alone	CRLM	Median OS<br>5-year OS	61.3m vs 54.3m<br>51.2% vs 47.8%

NCT22944367	Phase 3 (257)	Cetuximab +chemotherapy vs chemotherapy alone	CRLM	Median PFS<br>Median OS	15.5m vs 22.2m<br>55.4m vs 81m

DFS=disease-free survival. OS=overall survival. FOLFOX4=folinic acid+fluorouracil+oxaliplatin. mFOLFOX6= leucovorin+fluorouracil+ oxaliplatin

4.6 Immunotherapy

In addition to anti-angiogenesis therapy, immunotherapy becomes an attractive therapeutic option as more progress has been made in the exploration of immune checkpoint in several cancer types, mainly including DNA mismatch repair defects(dMMR)/high microsatellite instability (MSI-H) CRC and high tumor infiltrating lymphocyte (TIL) tumors. By contrast, immune checkpoint monotherapy has not demonstrated significant clinical success in patients with strong mismatch repair capacity (pMMR)/low microsatellite instability (MSI-L) CRC. Immune checkpoint drugs target the inhibitory receptors present on T cells, such as PD1, LAG3 and cytotoxic T-lymphocyte associated antigen 4 (CTLA-4).[365] These cells mainly exist at the tumor-stroma interface, while pMMR/MSI-L CRC shows a conventional morphology without obvious infiltrating lymphocyte TIL.[366]

tumor

PD1, a novel member of the immunoglobulin gene superfamily, has two functional ligands: PDL1 and programmed cell death ligand 2(PDL2).[367, 368] PD1/PDL1 signaling pathway can inhibit the activation of T effector cell while inhibiting the production of interferon gamma and the release of interleukin (IL)-2 and other inflammatory cytokines, thereby enabling cancer cells to escape from the host's antitumor immunosurveillance.[369, 370]Masugi et al.[371] reported Tumor PD1 expression was inversely associated with MSI-H in CRC. However, Wyss et al.[372]reported stromal PDL1 might function as a prognostic marker in CRC patients independent of microsatellite-stable (MSS) or with

MSI-L. Masugi et al.[371] found that stromal PDL1 expression was only present in 5% CRC patients, but the studies by Wyss et al.[372] and Taube et al.[373] displayed a stromal PDL1 expression rate of 60.9% and 50%, respectively. However, epithelial PDL1 staining is less common in CRC than in other solid tumors, such as melanoma.[374] While there is an inequality of PDL1 expression between primary tumors and metastases in melanoma patients,[375] PDL1 expression in primary colon tumors largely corresponds to matched liver metastases.[372]Another study revealed the gene networks of EMT, angiogenesis, immune-suppression and T cell exhaustion are the key events closely associated with CRC metastasis and intrinsic anti-PD1 resistance.[376]If anti-PDL1 treatment for metastatic colon cancer is needed, it may not be necessary to analyze PDL1 in liver metastases. The PDL1 and CRLM research is highly debated, therefore PD1/PDL1 signaling pathway may be closely related to CRLM and further exploration is needed.

A small number of patients with MSI-H CRC were sensitive to immune checkpoint blockade with antibodies to PD1/PDL1.[377, 378] The incidence of dMMR in CRLM is also low.[379] Recently, a study has found that tumor mutational burden(TMB) is a predictor of response to immune checkpoint inhibitors in the MSI-H population. Patients with high TMB may respond particularly well to immune checkpoint inhibitors, and these patients can be further selected to receive anti-PD1 monotherapy rather than combination therapy (such as nivolumab) as first-line treatment.[380] Nevertheless, in recent years, there have been new advances in immunotherapy for CRLM. Next, we summarized the latest progress in the clinical development of immune checkpoint blockade therapy for patients with CRLM.

CTLA-4 is predominantly expressed on T cells and inhibits the activation and response of T cells.[381] In a MSS highly aggressive orthotopic mouse model of CRC, dual inhibition of CTLA-4 and PDL1 resulted in the arrest of tumor growth and entirely blocked liver metastasis. Nevertheless, inhibition of CTLA-4 or PDL1 alone only moderately reduced metastatic diffusion of CRC cells.[382] Furthermore, pMMR CRLM differs immunologically from primary CRC in terms of immune infiltration.[383] Zhou et al.[384] focus on TIL regulated by the inhibitory receptor-ligand pathway regulates MMR-proficient CRLM. They found that blocking LAG3 enhances tumor-infiltrating T-

cell responses of MMR-proficient CRLM, and may thus be a new target of immunotherapy for CRLM.

Unlike primary CRC, tumor-infiltrating regulatory T cells (Ti-Treg) in liver metastases effectively inhibit tumor-specific T cell responses by expressing high levels of Treg-related molecule glucocorticoid-induced tumor necrosis factor receptor (GITR) and CTLA-4.[385, 386] It has demonstrated that treating CRLM by inhibiting the inhibitory receptor CTLA4 with antibodies and activating the stimulant receptor GITR with natural ligand of GITR can reduce Ti-Treg-mediated inhibition, thereby restoring effector T cell proliferation and cytokine formation.[387] More importantly, compared with any single treatment, the low-dose combination therapy of the two molecules showed a stronger ability to restore T cell function. Based on gene ontology and Kyoto Encyclopedia of Genes and Genomes pathway analyses, Liu et al.[388] identified some immune genes *CCL20, CCL24* and *CD70* that were associated with CRLM, but needs further experimental validation.

Furthermore, the relationship of gut microbiome and immune system is nowadays rapidly changing and expanding. Host factors, including age, obesity and gut microbiome greatly affect the effectiveness of immunotherapy.[389, 390] Meanwhile, gut microbiome is closely linked to the development and progression of CRC. Lipopolysaccharide (LPS), an important product of intestinal Gram-negative microbiota, was also found to participate in the whole process of CRLM. Systemic inflammation caused by elevated LPS blood levels in the intestinal cavity and portal vein in CRC patients could increase the liver recruitment of cancer cells.[391] LPS enhanced CRLM by stimulating Toll-like receptor 4 signaling and increasing β1 integrin-mediated cell adhesion.[392] LPS was observed to promote the migration capacity of CRC cells by activating the SDF-1 α /CXCR4 axis and EMT.[391] These results revealed trapping LPS may prevent CRLM. Recently, Song et al.[194] reported nanotechnology-based trapping LPS in the orthotopic CRC tumor could promote T-cell infiltration into tumors and promote enhanced immunotherapy.

Antartina, an antitumor agent isolated from Deschampsia antarctica Desv, potently inhibited tumor

growth and liver metastases in an immunocompetent colorectal carcinoma mice model.[393] Antartina induced a potent specific cytotoxic T-cell response against CRC and a long-lasting antitumor immunity. Antartina can induce antitumor immunity against CRLM. Based on the results of one phase III multicenter trial, Keytruda is superior to chemotherapy in the first-line treatment of MSI-H or dMMR CRC, resulting in longer progression-free survival and fewer treatment-related adverse events.[394]

4.7 Other potential therapies

Current chemotherapy for CRLM is still not desirable owing to off-target effect. Therefore, it is necessary to develop new methods to improve target effect or even replace the existing CRLM chemotherapy. Zhao et al.[395] found $\alpha5\beta1$ integrin receptor expression on metastatic cells was higher than that of the original cells from orthotopic tumors. They synthesized RPM-conjugated, $\alpha5\beta1$-targeted micelles (RPM-CSOSA), which could enhance the cellular internalization and distribution in metastatic lesions by binding to $\alpha5\beta1$ integrin. Doxorubicin (DOX) was a potent cytotoxic drug and curcumin (CUR) exerts its anti-cancer effects as chemosensitizer.[396] Zhao et al.[395] also found that the therapy of RPM-CSOSA/DOX and RPM-CSOSA/CUR significantly inhibited the progression of liver metastasis in vivo and in vitro.

Endothelin, a coreceptor of transforming growth factor- β , is preferentially expressed in solid tumor angiogenic endothelial cells.[397] Serum endoglin is a useful marker for monitoring early signs of CRC metastasis.[398] As an endoglin neutralizing antibody, TRC105 binds to human endoglin with high affinity and is associated with antibody-dependent cell-mediated cytotoxicity.[399] Targeting endoglin with TRC105 strongly inhibits metastatic spread of breast cancer in vivo.[400] Similarly, Targeting endoglin using TRC105 decreased metastatic spread of CRC cells to the mouse liver.[401] In a phase I first-in-human study ,TRC105 showed clinical efficacy on preexisting metastases in 2 patients.[402] Collectively, these results demonstrated that in addition to endothelial cells, targeting endoglin to CAFs may be a potent method to prevent metastases formation, and emphasizes the potential of TRC105 for the treatment of metastatic tumors, not just a classic anti-vascular generate drugs.

The cytoskeleton of the cell and ECM determine the stiffness of the tissue.[403, 404] Highly activated metastasis-associated fibroblasts cause the ECM to stiffen, which in turn increase tissue stiffness. Metastasis stiffness influences the effect of anti-angiogenic therapy on intra-tumoral blood vessel reduction. Shen et al.[405] reported that anti-hypertensive drugs targeting the renin-angiotensin system (anti-RAS) in combination with bevacizumab could significantly improve anti-angiogenic efficacy in CRLM. The mechanism is that anti-RAS inhibit fibroblast contraction and ECM deposition, thereby reducing the hardening of liver metastases and enhancing the anti-angiogenic effect of bevacizumab. This research highlights a new mechanism of action for anti-RAS drugs in cancer that could lead to new therapeutics.

The Cyclin-dependent kinase 8 (CDK8) is an early clinical stage drug that targets the overexpression in colon cancer.[406] From Liang et al.[407], inhibition of CDK8 almost has no effect on the growth of CRC cells and orthotopic transplanted tumors in subcutaneous, splenic, or cecum, but significantly inhibited the liver metastases of mouse and human colon cancer cells. CDK8 mediated CRLM due to down-regulating of matrix metalloproteinase (MMP) inhibitor TIMP3 via TGF β /SMAD-driven expression of a TIMP3-targeting microRNA, miR-181b, along with promotion of MMP3 in murine or MMP9 in human colon cancer cells via Wnt/ β -catenin-driven transcription.[407] This study illustrated that CDK8-targeting drugs can be used in the treatment of CRLM in the future.

We also would like to point out that the new development in cancer nanotechnology and photodynamic therapy (PDT) may provide potential solutions for CRC diagnosis and treatment.[408] PDT has been exploited as a promising cancer treatment modality for many years. PDT can destroy cancer cells by generating reactive oxygen species (ROS) when photosensitizers are irradiated with light of suitable wavelength. PDT has the benefit of dual targeting by the drugs themselves and light, so its side-effects are much lower compared with Chemotherapy or radiation therapy. However, in traditional PDT, the light from the applied high-frequency source is scattered and cannot penetrate deep tissue, which limits its clinical application.[409] Several possible solutions have been proposed to solve this problem, such as inventing new photosensitizers that use near infrared light and X-rays,

and developing up-conversion nanoparticles.[410-412] Among them, X-ray induced photodynamic therapy has attracted much attention because of its unlimited tissue penetration ability.[408,413-416] Most recently, Chen et al. reported Cu-Cy, $Cu_3Cl(SR)_2$ (R= $CH_2CH_2NH_2$),[417,418] a new type of sensitizer, has strong luminescence[417,419] and can produce ROS under UV irradiation,[417,420] X-rays,[421-423] microwave radiation,[424-428] and ultrasound.[429] All these observations reported so far suggest that the Cu-Cy NPs are a new type of sensitizers with potential applications for infection inactivation[430] and antitumor therapies.[431] The most recent studies prove that this new Cu-Cy nanoparticle possess anti-tumoral effects and potential molecular mechanisms on B16 melanoma through X-PDT and X-ray induced anti-tumor immunity,[432] and most importantly is that X-PDT is exceptionally effective with clinical X-rays used for irradiation in clinic settings.[433] We believe that these new technologies could provide good solutions for CRC treatment.

Perspective

CRC is still a common cancer and liver metastasis is the main cause of death in CRC patients. The treatment of advanced CRLM remains a major challenge. Previous studies have determined the main steps of the metastasis process. The role of TME, the dysregulation of NcRNAs, and the activation of various signaling pathways are closely related to liver metastasis. However, due to the lack of adequate experimental models to detect this complex process fully and continuously, the molecular mechanisms involved in the formation of CRLM have still much room for exploration. Detailed molecular mechanisms that mediate CRC metastasis to liver contribute to early detection and prevention. Future work should involve clarifying CRLM, based on the molecular mechanisms and clinical characteristics, which guide clinical precision treatment. At the same time, combined treatment and multidisciplinary cooperation should be established. Intervention at the early stage of liver metastasis, such as the stage of metastasis and colonization, will be more beneficial to improve patient survival. In addition, clinical and experimental data strongly suggest that postoperative tumor recurrence remains a possibility, therefore adjuvant targeted therapy is necessary for patients with postoperative resection. Defining high risk factors for CRLM, accurate selection of high-risk

individuals and minimizing the controllable risk factors are essential for the prevention of CRLM and further reducing mortality of CRC. It is also possible that the new development in cancer nanotechnology like nanoparticle self-lighting photodynamic therapy may be a new hope for CRC treatment.

STTT-Alice

ORIGINALITY REPORT

32%
SIMILARITY INDEX

PRIMARY SOURCES

	Source	Words	%
1	www.ncbi.nlm.nih.gov<br>Internet	392 words	3%
2	www.science.gov<br>Internet	160 words	1%
3	www.spandidos-publications.com<br>Internet	102 words	1%
4	Li Ren, Dexiang Zhu, Al B. Benson, Bernard Nordlinger et al. "Shanghai international consensus on diagnosis and comprehensive treatment of colorectal liver metastases (version 2019)", European Journal of Surgical Oncology, 2020<br>Crossref	87 words	1%
5	www.frontiersin.org<br>Internet	82 words	1%
6	www.jacbs.org.tw<br>Internet	82 words	1%
7	www.jove.com<br>Internet	76 words	1%
8	aimlab.cs.uoregon.edu<br>Internet	73 words	< 1%

9	www.dovepress.com<br>Internet	73 words — < 1%
10	Xiao-Yu Zhou, Bing Luo, Ze-Kun Jiang, Ying-Kang Xie, Feng-Cong Wu, Jiong-Qiang Huang, Jing-Song Chen. "Non-coding RNAS and colorectal cancer liver metastasis", Molecular and Cellular Biochemistry, 2020<br>Crossref	71 words — < 1%
11	www.cuilab.cn<br>Internet	70 words — < 1%
12	epub.ub.uni-muenchen.de<br>Internet	66 words — < 1%
13	www.pubfacts.com<br>Internet	66 words — < 1%
14	Ying Shen, Xiaohong Wang, Junyan Lu, Martin Salfenmoser et al. "Reduction of Liver Metastasis Stiffness Improves Response to Bevacizumab in Metastatic Colorectal Cancer", Cancer Cell, 2020<br>Crossref	65 words — < 1%
15	mdpi.com<br>Internet	64 words — < 1%
16	cancerres.aacrjournals.org<br>Internet	63 words — < 1%
17	Shihong Shao, Yun Zhu, Tingting Meng, Yupeng Liu, Yun Hong, Ming Yuan, Hong Yuan, Fuqiang Hu. " Targeting High Expressed α β Integrin in Liver Metastatic Lesions To Resist Metastasis of Colorectal Cancer by RPM Peptide-Modified Chitosan-Stearic Micelles ", Molecular Pharmaceutics, 2018<br>Crossref	61 words — < 1%

18 Jacqueline Wyss, Bastian Dislich, Viktor H. Koelzer, José A. Galván et al. "Stromal PD-1/PD-L1 Expression Predicts Outcome in Colon Cancer Patients", Clinical Colorectal Cancer, 2019 — 59 words — < 1%
Crossref

19 Jie Cui, Yukimatsu Toh, Soohyun Park, Wangsheng Yu et al. "Drug Conjugates of Antagonistic R-Spondin 4 Mutant for Simultaneous Targeting of Leucine-Rich Repeat-Containing G Protein-Coupled Receptors 4/5/6 for Cancer Treatment", Journal of Medicinal Chemistry, 2021 — 54 words — < 1%
Crossref

20 Yunbin Zhang, Jingjing Song, Zhongwei Zhao, Mengxuan Yang, Ming Chen, Chenglong Liu, Jiansong Ji, Di Zhu. "Single-cell transcriptome analysis reveals tumor immune microenvironment heterogenicity and granulocytes enrichment in colorectal cancer liver metastases", Cancer Letters, 2020 — 54 words — < 1%
Crossref

21 www.aging-us.com — 51 words — < 1%
Internet

22 Marie Lafitte, Céline Lecointre, Serge Roche. "Roles of exosomes in metastatic colorectal cancer", American Journal of Physiology-Cell Physiology, 2019 — 50 words — < 1%
Crossref

23 Dongxu Wang, Jie Liu, Tingting Huo, Yaowen Tian, Lei Zhao. "The role of microRNAs in colorectal liver metastasis: Important participants and potential clinical significances", Tumor Biology, 2017 — 49 words — < 1%
Crossref

24	Nermeen M. Omran, Sherif M. El - Sherbini, Osama Hegazy, Ahmed A. Elshaarawy, Roba M. Talaat. "Crosstalk between miR - 215 and epithelial - mesenchymal transition specific markers (E - cadherin and N - cadherin) in different stages of chronic HCV Infection", Journal of Medical Virology, 2019<br>Crossref	47 words — < 1%
25	Wantong Song, Karthik Tiruthani, Ying Wang, Limei Shen et al. "Trapping of Lipopolysaccharide to Promote Immunotherapy against Colorectal Cancer and Attenuate Liver Metastasis", Advanced Materials, 2018<br>Crossref	47 words — < 1%
26	Xiaohui Zhu, Feifei Wang, Xuehui Wu, Zhou Li et al. "FBX8 promotes metastatic dormancy of colorectal cancer in liver", Cell Death & Disease, 2020<br>Crossref	46 words — < 1%
27	scholars.uab.edu<br>Internet	46 words — < 1%
28	pdfs.semanticscholar.org<br>Internet	44 words — < 1%
29	"Textbook of Gastrointestinal Oncology", Springer Science and Business Media LLC, 2019<br>Crossref	43 words — < 1%
30	Yun Teng, Yi Ren, Xin Hu, Jingyao Mu et al. "MVP-mediated exosomal sorting of miR-193a promotes colon cancer progression", Nature Communications, 2017<br>Crossref	43 words — < 1%
31	A. E. Moore, A. Greenhough, H. R. Roberts, D. J. Hicks, H. A. Patsos, A. C. Williams, C. Paraskeva.	42 words — < 1%

"HGF/Met signalling promotes PGE2 biogenesis via regulation of COX-2 and 15-PGDH expression in colorectal cancer cells", Carcinogenesis, 2009
Crossref

32 Charlotte Kopitz, Michael Gerg, Obul Reddy Bandapalli, Dilek Ister et al. "Tissue Inhibitor of Metalloproteinases-1 Promotes Liver Metastasis by Induction of Hepatocyte Growth Factor Signaling", Cancer Research, 2007 — 41 words — < 1%
Crossref

33 ilse.kobv.de — 40 words — < 1%
Internet

34 www.nature.com — 40 words — < 1%
Internet

35 theoncologist.alphamedpress.org — 39 words — < 1%
Internet

36 Kuei-Yang Hsiao, Ya-Chi Lin, Sachin Kumar Gupta, Ning Chang, Laising Yen, H. Sunny Sun, Shaw-Jenq Tsai. "Noncoding Effects of Circular RNA CCDC66 Promote Colon Cancer Growth and Metastasis", Cancer Research, 2017 — 38 words — < 1%
Crossref

37 link.springer.com — 38 words — < 1%
Internet

38 Feng Du, Xiaowei Li, Weibo Feng, Chenyang Qiao et al. "SOX13 promotes colorectal cancer metastasis by transactivating SNAI2 and c-MET", Oncogene, 2020 — 37 words — < 1%
Crossref

39 www.hs-nb.de — 36 words — < 1%
Internet

40 Jianfeng Guo, Zhuo Yu, Manisit Das, Leaf Huang. "Nano Codelivery of Oxaliplatin and Folinic Acid Achieves Synergistic Chemo-Immunotherapy with 5-Fluorouracil for Colorectal Cancer and Liver Metastasis", ACS Nano, 2020 — 34 words — < 1%
Crossref

41 pubmed.ncbi.nlm.nih.gov — 34 words — < 1%
Internet

42 www.degruyter.com — 33 words — < 1%
Internet

43 Mengjing Fan, Ximei Ma, Feifan Wang, Zhuha Zhou, Jing Zhang, Difan Zhou, Yiyang Hong, Yihong Wang, Guanyu Wang, Qinghua Dong. "MicroRNA-30b-5p functions as a metastasis suppressor in colorectal cancer by targeting Rap1b", Cancer Letters, 2020 — 30 words — < 1%
Crossref

44 Xiangqi Meng, Jiangxue Wu, Changchuan Pan, Hui Wang et al. "Genetic and Epigenetic Down-regulation of MicroRNA-212 Promotes Colorectal Tumor Metastasis via Dysregulation of MnSOD", Gastroenterology, 2013 — 30 words — < 1%
Crossref

45 Yet-Ran Chen, Hsueh-Fen Juan, Hsuan-Cheng Huang, Hsin-Hung Huang et al. "Quantitative Proteomic and Genomic Profiling Reveals Metastasis-Related Protein Expression Patterns in Gastric Cancer Cells", Journal of Proteome Research, 2006 — 30 words — < 1%
Crossref

46 spandidos-publications.com — 30 words — < 1%
Internet

47 www.mdpi.com
Internet
30 words — < 1%

48 jhoonline.biomedcentral.com
Internet
29 words — < 1%

49 www.researchgate.net
Internet
29 words — < 1%

50 A.B. Schrock, C. Ouyang, J. Sandhu, E. Sokol et al. "Tumor mutational burden is predictive of response to immune checkpoint inhibitors in MSI-high metastatic colorectal cancer", Annals of Oncology, 2019
Crossref
28 words — < 1%

51 Diana Gulei, Alexandra Iulia Irimie, Roxana Cojocneanu-Petric, Joachim L. Schultze, Ioana Berindan-Neagoe. "Exosomes—Small Players, Big Sound", Bioconjugate Chemistry, 2018
Crossref
28 words — < 1%

52 Lei Shi, Rene Jackstadt, Helge Siemens, Huihui Li, Thomas Kirchner, Heiko Hermeking. "p53-Induced miR-15a/16-1 and AP4 Form a Double-Negative Feedback Loop to Regulate Epithelial–Mesenchymal Transition and Metastasis in Colorectal Cancer", Cancer Research, 2014
Crossref
28 words — < 1%

53 Dmitry I. Gabrilovich. "Myeloid-Derived Suppressor Cells", Cancer Immunology Research, 2017
Crossref
27 words — < 1%

54 Luyu Yang, Lu Liu, Rui Zhang, Jun Hong, Yaping Wang, Jian Wang, Jieliang Zuo, Jubo Zhang, Jinhong Chen, Hankun Hao. "IL-8 mediates a positive loop
27 words — < 1%

connecting increased neutrophil extracellular traps (NETs) and colorectal cancer liver metastasis", Journal of Cancer, 2020
Crossref

---

55 Shulin Huang, Xiaohui Tan, Zhongcheng Huang, Zihua Chen, Paul Lin, Sidney W. Fu. "microRNA biomarkers in colorectal cancer liver metastasis", Journal of Cancer, 2018 — 27 words — < 1%
Crossref

---

56 "“Abstracts of the 78th Annual Meeting of the Japanese Cancer Association; 2019 Sept 26 ‐ 28; Kyoto, Japan” as Cancer Science, Supplement 1, Vol 110 (2019)", Cancer Science, 2019 — 26 words — < 1%
Crossref

---

57 Jingkun Zhao, Baochi Ou, Dingpei Han, Puxiongzhi Wang et al. "Tumor-derived CXCL5 promotes human colorectal cancer metastasis through activation of the ERK/Elk-1/Snail and AKT/GSK3β/β-catenin pathways", Molecular Cancer, 2017 — 26 words — < 1%
Crossref

---

58 "Non-coding RNAs in Colorectal Cancer", Springer Science and Business Media LLC, 2016 — 25 words — < 1%
Crossref

---

59 Jianmin Xu, Jia Fan, Xinyu Qin, Jianqiang Cai, Jin Gu, Shan Wang, Xishan Wang, Suzhan Zhang, Zhongtao Zhang. "Chinese guidelines for the diagnosis and comprehensive treatment of colorectal liver metastases (version 2018)", Journal of Cancer Research and Clinical Oncology, 2018 — 25 words — < 1%
Crossref

---

60 cgp.iiarjournals.org — 25 words — < 1%
Internet

61	repositoriosdigitales.mincyt.gob.ar<br>Internet	25 words — < 1%
62	www.fedarch.org<br>Internet	25 words — < 1%
63	Hao-Xiang Tan, Wei-Zhi Gong, Kai Zhou, Zhi-Gang Xiao et al. "CXCR4/TGF-β1 mediated hepatic stellate cells differentiation into carcinoma-associated fibroblasts and promoted liver metastasis of colon cancer", Cancer Biology & Therapy, 2019<br>Crossref	24 words — < 1%
64	Wei Zhang, Bo Wang, Quan Wang, Zhen Zhang, Zhanlong Shen, Yingjiang Ye, Kewei Jiang, Shan Wang. "Lnc-HSD17B11-1:1 Functions as a Competing Endogenous RNA to Promote Colorectal Cancer Progression by Sponging miR-338-3p to Upregulate MACC1", Frontiers in Genetics, 2020<br>Crossref	24 words — < 1%
65	Yujia Xia, Jiayi He, Hongji Zhang, Han Wang et al. "AAV ‐ mediated gene transfer of DNase I in the liver of mice with colorectal cancer reduces liver metastasis and restores local innate and adaptive immune response", Molecular Oncology, 2020<br>Crossref	24 words — < 1%
66	Boyang Xu, Ziqi Peng, Guanyu Yan, Ningning Wang, Moye Chen, Xue Yao, Mingjun Sun, Yue An. "Establishment and Validation of a Genetic Label Associated With M2 Macrophage Infiltration to Predict Survival in Patients With Colon Cancer and to Assist in Immunotherapy", Frontiers in Genetics, 2021<br>Crossref	23 words — < 1%

67 Wen-Ze Qiu, Hai-Bo Zhang, Wei-Xiong Xia, Liang-Ru Ke et al. "The CXCL5/CXCR2 axis contributes to the epithelial-mesenchymal transition of nasopharyngeal carcinoma cells by activating ERK/GSK-3β/snail signalling", Journal of Experimental & Clinical Cancer Research, 2018 — 23 words — < 1%
Crossref

68 www.cancerindex.org — 23 words — < 1%
Internet

69 Lixin Ma, Haiquan Qiao, Changjun He, Qian Yang, Chun Hei Antonio Cheung, Jagat R. Kanwar, Xueying Sun. "Modulating the interaction of CXCR4 and CXCL12 by low-molecular-weight heparin inhibits hepatic metastasis of colon cancer", Investigational New Drugs, 2010 — 22 words — < 1%
Crossref

70 E Agarwal, C M Robb, L M Smith, M G Brattain, J Wang, J D Black, S Chowdhury. "Role of Akt2 in regulation of metastasis suppressor 1 expression and colorectal cancer metastasis", Oncogene, 2017 — 21 words — < 1%
Crossref

71 Fangfang Fu, Weiqin Jiang, Linfu Zhou, Zhi Chen. "Circulating Exosomal miR-17-5p and miR-92a-3p Predict Pathologic Stage and Grade of Colorectal Cancer", Translational Oncology, 2018 — 21 words — < 1%
Crossref

72 Madelon Paauwe, Mark J.A. Schoonderwoerd, Roxan F.C.P. Helderman, Tom J. Harryvan et al. "Endoglin Expression on Cancer-Associated Fibroblasts Regulates Invasion and Stimulates Colorectal Cancer Metastasis", Clinical Cancer Research, 2018 — 21 words — < 1%
Crossref

73 Kai Jiang, Haiyan Chen, Yimin Fang, Liubo Chen et al. "Exosomal ANGPTL1 attenuates colorectal cancer liver metastasis by regulating Kupffer cell secretion pattern and impeding MMP9 induced vascular leakiness", Journal of Experimental & Clinical Cancer Research, 2021 — 20 words — < 1%
Crossref

74 Xuetong Rong, Haiyi Liu, Hongmei Yu, Jian Zhao, Jie Wang, Yusheng Wang. "Efficacy of Apatinib Combined with FOLFIRI in the First-Line Treatment of Patients with Metastatic Colorectal Cancer", Research Square Platform LLC, 2021 — 20 words — < 1%
Crossref Posted Content

75 encyclopedia.pub — 20 words — < 1%
Internet

76 Soudeh Ghafouri-Fard, Bashdar Mahmud Hussen, Elham Badrlou, Atefe Abak, Mohammad Taheri. "MicroRNAs as important contributors in the pathogenesis of colorectal cancer", Biomedicine & Pharmacotherapy, 2021 — 19 words — < 1%
Crossref

77 Wei Feng, Wei Zong, Yi Li, Xianjuan Shen, Xiaopeng Cui, Shaoqing Ju. "Abnormally expressed long noncoding RNA B3GALT5 ‐ AS1 may serve as a biomarker for the diagnostic and prognostic of gastric cancer", Journal of Cellular Biochemistry, 2019 — 19 words — < 1%
Crossref

78 Liye Wang, Kwang Bog Cho, Yan Li, Gabriel Tao, Zuoxu Xie, Bin Guo. "Long Noncoding RNA (lncRNA)-Mediated Competing Endogenous RNA Networks Provide Novel Potential Biomarkers and Therapeutic Targets for Colorectal Cancer", International Journal of Molecular Sciences, 2019 — 18 words — < 1%
Crossref

79	fedorakg.kg.ac.rs<br>Internet	18 words — < 1%
80	journals.lww.com<br>Internet	18 words — < 1%
81	Jian‐feng Yao, Xiao‐jun Li, Li‐kun Yan, Sai He et al. "Role of HGF/c‐Met in the treatment of colorectal cancer with liver metastasis", Journal of Biochemical and Molecular Toxicology, 2019<br>Crossref	17 words — < 1%
82	Kai Jiang, Haiyan Chen, Yimin Fang, Liubo Chen et al. "Exosomal ANGPTL1 Attenuates CRC Liver Metastasis by Regulating Kupffer Cell Secretion Pattern and Impeding MMP9 induced vascular leakiness.", Research Square, 2020<br>Crossref Posted Content	17 words — < 1%
83	Kenji Kawada, Suguru Hasegawa, Teppei Murakami, Yoshiro Itatani et al. "Molecular mechanisms of liver metastasis", International Journal of Clinical Oncology, 2011<br>Crossref	17 words — < 1%
84	Kinan Drak Alsibai, Didier Meseure. "Tumor microenvironment and noncoding RNAs as co-drivers of epithelial-mesenchymal transition and cancer metastasis", Developmental Dynamics, 2017<br>Crossref	17 words — < 1%
85	www.cancerbio.net<br>Internet	17 words — < 1%
86	www.tandfonline.com<br>Internet	17 words — < 1%

87 "Virus Infection and Tumorigenesis", Springer Science and Business Media LLC, 2019 — 16 words — < 1%
Crossref

88 Dingzhi Wang, Lingchen Fu, Haiyan Sun, Lixia Guo, Raymond N. DuBois. "Prostaglandin E2 Promotes Colorectal Cancer Stem Cell Expansion and Metastasis in Mice", Gastroenterology, 2015 — 16 words — < 1%
Crossref

89 Guoying Zhou, Lisanne Noordam, Dave Sprengers, Michail Doukas et al. "Blockade of LAG3 enhances responses of tumor-infiltrating T cells in mismatch repair-proficient liver metastases of colorectal cancer", OncoImmunology, 2018 — 16 words — < 1%
Crossref

90 Yong Zhang, Karen E. Parrish, David R. Tortolani, Michael A. Poss et al. "Long-Acting Tumor-Activated Prodrug of a TGFβR Inhibitor", Journal of Medicinal Chemistry, 2021 — 16 words — < 1%
Crossref

91 Yoshinaga Okugawa, William M. Grady, Ajay Goel. "Epigenetic Alterations in Colorectal Cancer: Emerging Biomarkers", Gastroenterology, 2015 — 16 words — < 1%
Crossref

92 Yue Xie, Jing Zhao, Yanling Liang, Min Chen, Yihong Luo, Xiaobing Cui, Bo Jiang, Liang Peng, Xinying Wang. "MicroRNA-10b controls the metastasis and proliferation of colorectal cancer cells by regulating Krüppel-like factor 4", Artificial Cells, Nanomedicine, and Biotechnology, 2019 — 16 words — < 1%
Crossref

93 ebin.pub
Internet

16 words — < 1%

94 eprints.whiterose.ac.uk
Internet
16 words — < 1%

95 www.preprints.org
Internet
16 words — < 1%

96 www.researchsquare.com
Internet
16 words — < 1%

97 Liang, Jiaxin. "The Role of CDK8 in Metastatic Growth of Colon Cancer.", University of South Carolina, 2019
ProQuest
15 words — < 1%

98 Matteo Donadon, Kelly Hudspeth, Matteo Cimino, Luca Di Tommaso et al. "Increased Infiltration of Natural Killer and T Cells in Colorectal Liver Metastases Improves Patient Overall Survival", Journal of Gastrointestinal Surgery, 2017
Crossref
15 words — < 1%

99 Www.semanticscholar.org
Internet
15 words — < 1%

100 www.karger.com
Internet
15 words — < 1%

101 www.labome.org
Internet
15 words — < 1%

102 www.oncotarget.com
Internet
15 words — < 1%

103 www.thno.org
Internet

15 words — < 1%

104 Florian Schrötzlmair, Charlotte Kopitz, Birgit Halbgewachs, Fei Lu et al. "Tissue inhibitor of metalloproteinases-1-induced scattered liver metastasis is mediated by host-derived urokinase-type plasminogen activator", Journal of Cellular and Molecular Medicine, 2010
Crossref
14 words — < 1%

105 Haixing Liao, Muneeb Ahmed, Aurelia Markezana, Guohua Zeng, Matthias Stechele, Eithan Galun, S. Nahum Goldberg. "Thermal Ablation Induces Transitory Metastatic Growth by Means of the STAT3/c-Met Molecular Pathway in an Intrahepatic Colorectal Cancer Mouse Model", Radiology, 2020
Crossref
14 words — < 1%

106 Shuan S. Huang, Jung S. Huang. "TGF-β control of cell proliferation", Journal of Cellular Biochemistry, 2005
Crossref
14 words — < 1%

107 erc.endocrinology-journals.org
Internet
14 words — < 1%

108 www.deepdyve.com
Internet
14 words — < 1%

109 www.idexlab.com
Internet
14 words — < 1%

110 Hang Yin, Xiaoyuan Wang, Xue Zhang, Yan Wang et al. "Integrated analysis of long noncoding RNA associated-competing endogenous RNA as prognostic biomarkers in clear cell renal carcinoma", Cancer Science, 2018
Crossref
13 words — < 1%

111 Jiao Zheng, Min Yang, Jianghua Shao, Yanju Miao, Jiahuai Han, Jie Du. "Chemokine receptor CX3CR1 contributes to macrophage survival in tumor metastasis", Molecular Cancer, 2013 — 13 words — < 1%

Crossref

---

112 Karuna Ganesh, Harihar Basnet, Yasemin Kaygusuz, Ashley M. Laughney et al. "L1CAM defines the regenerative origin of metastasis-initiating cells in colorectal cancer", Nature Cancer, 2020 — 13 words — < 1%

Crossref

---

113 Laura Bracci, Francesco Lozupone, Isabella Parolini. "The role of exosomes in colorectal cancer disease progression and response to therapy", Cytokine & Growth Factor Reviews, 2020 — 13 words — < 1%

Crossref

---

114 M. Yamamoto, H. Kikuchi, M. Ohta, T. Kawabata, Y. Hiramatsu, K. Kondo, M. Baba, K. Kamiya, T. Tanaka, M. Kitagawa, H. Konno. "TSU68 Prevents Liver Metastasis of Colon Cancer Xenografts by Modulating the Premetastatic Niche", Cancer Research, 2008 — 13 words — < 1%

Crossref

---

115 Y. R. Park, S. L. Kim, M. R. Lee, S. Y. Seo, J. H. Lee, S. H. Kim, I. H. Kim, S. O. Lee, S. T. Lee, Sang Wook Kim. "MicroRNA-30a-5p (miR-30a) regulates cell motility and EMT by directly targeting oncogenic TM4SF1 in colorectal cancer", Journal of Cancer Research and Clinical Oncology, 2017 — 13 words — < 1%

Crossref

---

116 Youxiang Ding, Kun Hao, Zhaohe Li, Rong Ma, You Zhou, Zhou Zhou, Mian Wei, Yan Liao, Yao Dai, Yue Yang, Xiaobo Zhang, Li Zhao. "c‐Fos separation from Lamin A/C by GDF15 promotes colon cancer invasion and — 13 words — < 1%

metastasis in inflammatory microenvironment", Journal of Cellular Physiology, 2019
Crossref

117 en.wikipedia.org
Internet
13 words — < 1%

118 sure.sunderland.ac.uk
Internet
13 words — < 1%

119 Chao Fang, Chuanwen Fan, Cun Wang, Qiaorong Huang et al. " Prognostic value of CD133 CD54 CD44 circulating tumor cells in colorectal cancer with liver metastasis ", Cancer Medicine, 2017
Crossref
12 words — < 1%

120 Margarita Neganova, Junqi Liu, Yulia Aleksandrova, Sergey Klochkov, Ruitai Fan. "Therapeutic Influence on Important Targets Associated with Chronic Inflammation and Oxidative Stress in Cancer Treatment", Cancers, 2021
Crossref
12 words — < 1%

121 Mu Xu, Xueni Xu, Bei Pan, Xiaoxiang Chen et al. "LncRNA SATB2-AS1 inhibits tumor metastasis and affects the tumor immune cell microenvironment in colorectal cancer by regulating SATB2", Molecular Cancer, 2019
Crossref
12 words — < 1%

122 Seeliger, C, K Karpinski, AT Haug, H Vester, A Schmitt, JS Bauer, and M van Griensven. "Five Freely Circulating miRNAs and Bone Tissue miRNAs are Associated with Osteoporotic Fractures", Journal of Bone and Mineral Research, 2014.
Crossref
12 words — < 1%

123 Sian Chen, Xian Shen. "Long noncoding RNAs: functions and mechanisms in colon cancer", Molecular Cancer, 2020 — 12 words — < 1%
Crossref

124 Taicheng Zhou, Lili Wu, Ning Ma, Fuxin Tang, Zhuomin Yu, Zhipeng Jiang, Yingru Li, Zhen Zong, Kunpeng Hu. "SOX9-activated FARSA-AS1 predetermines cell growth, stemness, and metastasis in colorectal cancer through upregulating FARSA and SOX9", Cell Death & Disease, 2020 — 12 words — < 1%
Crossref

125 Tracey A. Martin, Malcolm D. Mason, Wen G. Jiang. "Hepatocyte Growth Factor Signaling in Cancer Metastasis", Current Signal Transduction Therapy, 2011 — 12 words — < 1%
Crossref

126 Zengyao Li, Jing Liu, Hang Chen, Ye Zhang, Haoze Shi, Longchang Huang, Jianxin Tao, Renhui Shen, Tong Wang. "Ferritin Light Chain (FTL) competes with long noncoding RNA Linc00467 for miR-133b binding site to regulate chemoresistance and metastasis of colorectal cancer", Carcinogenesis, 2019 — 12 words — < 1%
Crossref

127 cyberleninka.org — 12 words — < 1%
Internet

128 jeccr.biomedcentral.com — 12 words — < 1%
Internet

129 www.childtrends.org — 12 words — < 1%
Internet

130 Maximilian Zeidler, Kai K. Kummer, Clemens L. Schöpf, Theodora Kalpachidou, Georg Kern, M. Zameel Cader, Michaela Kress. "NOCICEPTRA: Gene and — 11 words — < 1%

microRNA signatures and their trajectories characterizing human iPSC-derived nociceptor maturation", Cold Spring Harbor Laboratory, 2021
Crossref Posted Content

131 Pedro Baleia. "8th International conference on Tumor Microenvironment, Lisbon, Portugal June 10-14, 2018", Cancer Microenvironment, 2018
Crossref
11 words — < 1%

132 Roshan, R.. "MicroRNAs: novel therapeutic targets in neurodegenerative diseases", Drug Discovery Today, 200912
Crossref
11 words — < 1%

133 X. Liu, D. Xu, Z. Liu, Y. Li, C. Zhang, Y. Gong, Y. Jiang, B. Xing. "THBS1 facilitates colorectal liver metastasis through enhancing epithelial–mesenchymal transition", Clinical and Translational Oncology, 2020
Crossref
11 words — < 1%

134 Yingkuan Shao, Ting Chen, Xi Zheng, Sheng Yang et al. "Colorectal cancer-derived small extracellular vesicles establish an inflammatory premetastatic niche in liver metastasis", Carcinogenesis, 2018
Crossref
11 words — < 1%

135 gbcrc.bioinfo-minzhao.org
Internet
11 words — < 1%

136 pesquisa.bvsalud.org
Internet
11 words — < 1%

137 test.mayocliniclabs.com
Internet
11 words — < 1%

138 www.wjgnet.com
Internet
11 words — < 1%

139 Bebernitz, Christina Elizabeth. "Interleukin 12 and Interleukin 18 Combination Improves Activation of Adoptively Transferred T Cells in Solid Tumors", Weill Medical College of Cornell University, 2021 — 10 words — < 1%
ProQuest

140 Dong Wang, Xiaohui Wang, Mahan Si, Juan Yang, Shiyue Sun, Haochen Wu, Shuxiang Cui, Xianjun Qu, Xinfeng Yu. "Exosome-encapsulated miRNAs contribute to CXCL12/CXCR4-induced liver metastasis of colorectal cancer by enhancing M2 polarization of macrophages", Cancer Letters, 2020 — 10 words — < 1%
Crossref

141 Nicola Huxley, Louise Crathorne, Jo Varley-Campbell, Irina Tikhonova et al. "The clinical effectiveness and cost-effectiveness of cetuximab (review of technology appraisal no. 176) and panitumumab (partial review of technology appraisal no. 240) for previously untreated metastatic colorectal cancer: a systematic review and economic evaluation", Health Technology Assessment, 2017 — 10 words — < 1%
Crossref

142 biotm.cis.udel.edu — 10 words — < 1%
Internet

143 effiloop.com — 10 words — < 1%
Internet

144 open.library.ubc.ca — 10 words — < 1%
Internet

145 patents.google.com — 10 words — < 1%
Internet

146 Aspros, Kirsten Grace Mueller. "Estrogen Receptor Beta Elicits Anti-cancer Effects in Triple Negative Breast Cancer Through Suppression of NFkB Signaling.", College of Medicine - Mayo Clinic, 2020 — 9 words — < 1%
ProQuest

147 Erika Martinelli, Stefania Napolitano, Davide Ciardiello, Fortunato Ciardiello, Teresa Troiani. "Optimization of the Development of Old and New EGFR and MAP Kinase Inhibitors for Colorectal Cancer", Current Colorectal Cancer Reports, 2014 — 9 words — < 1%
Crossref

148 Fang Sun, Jin-Zhi Wang, Ji-Jun Luo, Yu-Qin Wang, Qin Pan. "Exosomes in the Oncobiology, Diagnosis, and Therapy of Hepatic Carcinoma: A New Player of an Old Game", BioMed Research International, 2018 — 9 words — < 1%
Crossref

149 Inflammation and Immunity in Cancer, 2015. — 9 words — < 1%
Crossref

150 Ivana Samaržija. "Wnt Signaling Pathway Is among the Drivers of Liver Metastasis", Livers, 2021 — 9 words — < 1%
Crossref

151 MicroRNA Targeted Cancer Therapy, 2014. — 9 words — < 1%
Crossref

152 Nguyen, Linh T., and Pamela S. Ohashi. "Clinical blockade of PD1 and LAG3 — potential mechanisms of action", Nature Reviews Immunology, 2014. — 9 words — < 1%
Crossref

153 Wen G. Jiang. "Cell adhesion molecules in the formation of liver metastasis", Journal of Hepato-Biliary-Pancreatic Surgery, 1998 — 9 words — < 1%

Crossref

154 ascopubs.org
Internet
9 words — < 1%

155 cancerjournal.net
Internet
9 words — < 1%

156 elifesciences.org
Internet
9 words — < 1%

157 www.univadis.co.uk
Internet
9 words — < 1%

158 "81st Annual Meeting of the American Thyroid Association MEETING ABSTRACTS & AGENDA", Thyroid, 10/2011
Crossref
8 words — < 1%

159 "E-Poster Presentations - A4) Lower GI", Journal of Gastroenterology and Hepatology, 2017
Crossref
8 words — < 1%

160 Advances in Delivery Science and Technology, 2014.
Crossref
8 words — < 1%

161 Amin Saberinia, Amin Alinezhad, Fatemeh Jafari, Setareh Soltany, Reza Akhavan Sigari. "Oncogenic miRNAs and target therapies in colorectal cancer", Clinica Chimica Acta, 2020
Crossref
8 words — < 1%

162 Changhong He, Xia Wang, Meichun Du, Yanjun Dong. "LncRNA MSC-AS1 Promotes Colorectal Cancer Progression by Regulating miR-325/TRIM14 Axis", Journal of Oncology, 2021
Crossref
8 words — < 1%

163 Federica Zoratto, L. Rossi, E. Giordani, M. Strudel, A. Papa, S. Tomao. "From conventional chemotherapy to targeted therapy: use of monoclonal antibodies (moAbs) in gastrointestinal (GI) tumors", Tumor Biology, 2014 8 words — < 1%
Crossref

164 Guinn, Daphne Allyn. "Alterations and Mutations in Bruton's Tyrosine Kinase Affect the Transcriptional Profile and Phenotype of Chronic Lymphocytic Leukemia Cells", The Ohio State University, 2021 8 words — < 1%
ProQuest

165 Laura A. Tesmer. "Th17 cells in human disease", Immunological Reviews, 06/2008 8 words — < 1%
Crossref

166 M. Osaki, F. Takeshita, T. Ochiya. "MicroRNAs as biomarkers and therapeutic drugs in human cancer", Biomarkers, 2009 8 words — < 1%
Crossref

167 Mariko Shimokawa, Yuki Ohta, Shingo Nishikori, Mami Matano et al. "Visualization and targeting of LGR5+ human colon cancer stem cells", Nature, 2017 8 words — < 1%
Crossref

168 Songhe Guo, Jun Chen, Fangfang Chen, Qiuyao Zeng, Wan-Li Liu, Ge Zhang. " Exosomes derived from -infected colorectal cancer cells facilitate tumour metastasis by selectively carrying miR-1246/92b-3p/27a-3p and CXCL16 ", Gut, 2020 8 words — < 1%
Crossref

169 Suresh Kumar Gulla, Rajesh Kotcherlakota, Sahithi Nimushakavi, Narendra Varma Nimmu et al. "Au- 8 words — < 1%

CGKRK Nanoconjugates for Combating Cancer through T-Cell-Driven Therapeutic RNA Interference", ACS Omega, 2018
Crossref

---

170 Tang, Ying, Ze-Min Ling, Rao Fu, Ying-Qin Li, Xiao Cheng, Fa-Huan Song, Hao-Xuan Luo, and Li-Hua Zhou. "Time-specific microRNA changes during spinal motoneuron degeneration in adult rats following unilateral brachial plexus root avulsion: ipsilateral vs. contralateral changes", BMC Neuroscience, 2014.
Crossref

8 words — < 1%

---

171 Zhengshuo Li, Xiaoyue Zhang, Can Liu, Jian Ma. "Non-immune Cell Components in the Gastrointestinal Tumor Microenvironment Influencing Tumor Immunotherapy", Frontiers in Cell and Developmental Biology, 2021
Crossref

8 words — < 1%

---

172 Zhongxi Lyu, Yongming Guo, Yinan Gong, Wen Fan et al. "The Role of Neuroglial Crosstalk and Synaptic Plasticity-Mediated Central Sensitization in Acupuncture Analgesia", Neural Plasticity, 2021
Crossref

8 words — < 1%

---

173 cslide.ctimeetingtech.com
Internet

8 words — < 1%

---

174 docero.net
Internet

8 words — < 1%

---

175 hal.archives-ouvertes.fr
Internet

8 words — < 1%

---

176 research.kindai.ac.jp
Internet

8 words — < 1%

177 www.jneurosci.org
Internet
8 words — < 1%

178 Brian Belyea, Julie Grondin Kephart, Jordan Blum, David G. Kirsch, Corinne M. Linardic. "Embryonic Signaling Pathways and Rhabdomyosarcoma: Contributions to Cancer Development and Opportunities for Therapeutic Targeting", Sarcoma, 2012
Crossref
7 words — < 1%

179 Chunfeng Ren, Zhenmin Zhang, Shunhua Wang, Weitao Zhu, Peiguo Zheng, Wanhai Wang. "Circular RNA hsa_circ_0001178 facilitates the invasion and metastasis of colorectal cancer through upregulating ZEB1 via sponging multiple miRNAs", Biological Chemistry, 2020
Crossref
7 words — < 1%

180 Gianluca Lopez, Francesca Boggio, Stefano Ferrero, Nicola Fusco, Alessandro Del Gobbo. "Molecular and Immunohistochemical Markers with Prognostic and Predictive Significance in Liver Metastases from Colorectal Carcinoma", International Journal of Molecular Sciences, 2018
Crossref
7 words — < 1%

181 Huihui Li, Matjaz Rokavec, Longchang Jiang, David Horst, Heiko Hermeking. "Antagonistic Effects of p53 and HIF1A on microRNA-34a Regulation of PPP1R11 and STAT3 and Hypoxia-induced Epithelial to Mesenchymal Transition in Colorectal Cancer Cells", Gastroenterology, 2017
Crossref
7 words — < 1%

182 Mariana Malvicini, Ana Gutierrez-Moraga, Marcelo M. Rodriguez, Sofia Gomez-Bustillo et al. " A Tricin Derivative from Desv. Inhibits Colorectal Carcinoma Growth and Liver Metastasis through the Induction of a Specific Immune Response ", Molecular Cancer Therapeutics, 2018
Crossref
7 words — < 1%

183 Simoneau, Eve B.. "Colorectal Cancer Liver Metastasis Progression after Portal Vein Embolization.", McGill University (Canada), 2021 — 7 words — < 1%
ProQuest

184 Tonini, Giuseppe, Marco Imperatori, Bruno Vincenzi, Anna Frezza, and Daniele Santini. "Rechallenge therapy and treatment holiday: different strategies in management of metastatic colorectal cancer", Journal of Experimental & Clinical Cancer Research, 2013. — 7 words — < 1%
Crossref

185 Yoshinori Kato, Hiraku Onishi, Yoshiharu Machida. "Lactosaminated and intact N-succinyl-chitosans as drug carriers in liver metastasis", International Journal of Pharmaceutics, 2001 — 7 words — < 1%
Crossref

186 Zhang, Z, Z Li, Y Li, and A Zang. "MicroRNA and signaling pathways in gastric cancer", Cancer Gene Therapy, 2014. — 7 words — < 1%
Crossref

187 Amir Mehrgou, Shima Ebadollahi, Khaled Seidi, Mohammad Hosein Ayoubi-Joshaghani et al. "Roles of miRNAs in Colorectal Cancer: Therapeutic Implications and Clinical Opportunities", Advanced Pharmaceutical Bulletin, 2020 — 6 words — < 1%
Crossref

188 Bingqing Huang, Huipeng Yang, Xixi Cheng, Dan Wang et al. "tRF/miR-1280 Suppresses Stem Cell–like Cells and Metastasis in Colorectal Cancer", Cancer Research, 2017 — 6 words — < 1%
Crossref

189 Cancer Metastasis – Biology and Treatment, 2010.

Crossref

6 words — < 1%

190 H.-Y. Chen, Y.-M. Lin, H.-C. Chung, Y.-D. Lang et al. "miR-103/107 Promote Metastasis of Colorectal Cancer by Targeting the Metastasis Suppressors DAPK and KLF4", Cancer Research, 2012
Crossref

6 words — < 1%

191 Jing Chen, Qiuming Liu, Jiangang Xiao, Jianzhong Du. "EpCAM-Antibody-Labeled Noncytotoxic Polymer Vesicles for Cancer Stem Cells-Targeted Delivery of Anticancer Drug and siRNA", Biomacromolecules, 2015
Crossref

6 words — < 1%

192 Mahdi Hussain Al Bandar, Nam Kyu Kim. "Current status and future perspectives on treatment of liver metastasis in colorectal cancer", Oncology Reports, 2017
Crossref

6 words — < 1%

193 Maochao Luo, Xingyue Yang, Hai-Ning Chen, Edouard C. Nice, Canhua Huang. "Drug resistance in colorectal cancer: An epigenetic overview", Biochimica et Biophysica Acta (BBA) - Reviews on Cancer, 2021
Crossref

6 words — < 1%

194 Phanourios Tamamis, Christodoulos A. Floudas. "Elucidating a Key Component of Cancer Metastasis: CXCL12 (SDF-1α) Binding to CXCR4", Journal of Chemical Information and Modeling, 2014
Crossref

6 words — < 1%

195 Trung Vu, Pran Datta. "Regulation of EMT in Colorectal Cancer: A Culprit in Metastasis", Cancers, 2017
Crossref

6 words — < 1%

196 Ulrich H. Weidle, Fabian Birzele, Achim Krüger. "Molecular targets and pathways involved in liver metastasis of colorectal cancer", Clinical & Experimental Metastasis, 2015 — 6 words — < 1%
Crossref

EXCLUDE QUOTES	OFF	EXCLUDE MATCHES	OFF
EXCLUDE BIBLIOGRAPHY	OFF		
